# Altered white matter microstructure in 22q11.2 deletion syndrome: a multisite diffusion tensor imaging study

**DOI:** 10.1038/s41380-019-0450-0

**Published:** 2019-07-29

**Authors:** Julio E. Villalón-Reina, Kenia Martínez, Xiaoping Qu, Christopher R. K. Ching, Talia M. Nir, Deydeep Kothapalli, Conor Corbin, Daqiang Sun, Amy Lin, Jennifer K. Forsyth, Leila Kushan, Ariana Vajdi, Maria Jalbrzikowski, Laura Hansen, Rachel K. Jonas, Therese van Amelsvoort, Geor Bakker, Wendy R. Kates, Kevin M. Antshel, Wanda Fremont, Linda E. Campbell, Kathryn L. McCabe, Eileen Daly, Maria Gudbrandsen, Clodagh M. Murphy, Declan Murphy, Michael Craig, Beverly Emanuel, Donna M. McDonald-McGinn, Jacob A.S. Vorstman, Ania M. Fiksinski, Sanne Koops, Kosha Ruparel, David Roalf, Raquel E. Gur, J. Eric Schmitt, Tony J. Simon, Naomi J. Goodrich-Hunsaker, Courtney A. Durdle, Joanne L. Doherty, Adam C. Cunningham, Marianne van den Bree, David E. J. Linden, Michael Owen, Hayley Moss, Sinead Kelly, Gary Donohoe, Kieran C. Murphy, Celso Arango, Neda Jahanshad, Paul M. Thompson, Carrie E. Bearden

**Affiliations:** 1 Imaging Genetics Center, Mark and Mary Stevens Neuroimaging & Informatics Institute, Keck School of Medicine of the University of Southern California, Marina del Rey, CA USA; 2 Department of Child and Adolescent Psychiatry, Hospital General Universitario Gregorio Marañón, Universidad Complutense, School of Medicine, IiSGM, Madrid, Spain; 3 Centro de Investigación Biomédica en Red de Salud Mental (CIBERSAM), Madrid, Spain; 4 Universidad Europea de Madrid, Madrid, Spain; 5 Department of Psychiatry and Biobehavioral Sciences, Semel Institute for Neuroscience and Human Behavior, University of California at Los Angeles, Los Angeles, CA USA; 6 Department of Mental Health, Veterans Affairs Greater Los Angeles Healthcare System, Los Angeles, CA USA; 7 Department of Psychology, University of California at Los Angeles, Los Angeles, CA USA; 8 Department of Psychiatry, University of Pittsburgh, Pittsburgh, PA USA; 9 Department of Psychiatry & Neuropsychology, Maastricht University, Maastricht, Netherlands; 10 Department of Psychiatry and Behavioral Sciences, State University of New York, Upstate Medical University, Syracuse, NY USA; 11 Department of Psychology, Syracuse University, Syracuse, NY USA; 12 Priority Research Centre GrowUpWell, University of Newcastle, Newcastle, Australia; 13 School of Psychology, University of Newcastle, Newcastle, Australia; 14 UC Davis MIND Institute and Department of Psychiatry and Behavioral Sciences, Davis, CA USA; 15 Sackler Institute for Translational Neurodevelopment and Department of Forensic and Neurodevelopmental Sciences, King’s College London, Institute of Psychiatry, Psychology & Neuroscience, London, UK; 16 Behavioural and Developmental Psychiatry Clinical Academic Group, Behavioural Genetics Clinic, National Adult Autism and ADHD Service, South London and Maudsley Foundation NHS Trust, London, UK; 17 National Autism Unit, Bethlem Royal Hospital, Bethlem, UK; 18 Division of Human Genetics, Children’s Hospital of Philadelphia, Perelman School of Medicine, University of Pennsylvania, Philadelphia, PA USA; 19 Department of Psychiatry, Brain Center Rudolf Magnus, University Medical Center Utrecht, Utrecht, The Netherlands; 20 Program in Genetics and Genome Biology, The Hospital for Sick Children, Toronto, ON Canada; 21 Department of Psychiatry, University of Toronto, Toronto, ON Canada; 22 Department of Psychiatry, Rudolf Magnus Institute of Neuroscience, University Medical Center Utrecht, Utrecht, The Netherlands; 23 Clinical Genetics Research Program, Centre for Addiction and Mental Health, Toronto, ON Canada; 24 The Dalglish Family 22q Clinic for 22q11.2 Deletion Syndrome, Toronto General Hospital, University Health Network, Toronto, ON Canada; 25 Department of Psychiatry, University of Pennsylvania, Philadelphia, PA USA; 26 Department of Psychiatry, Perelman School of Medicine, University of Pennsylvania and Children’s Hospital of Philadelphia, Philadelphia, PA USA; 27 Departments of Radiology and Psychiatry, University of Pennsylvania, Philadelphia, PA USA; 28 Brigham Young University, Provo, UT USA; 29 Department of Neurology, University of Utah, Salt Lake City, UT USA; 30 MRC Centre for Neuropsychiatric Genetics and Genomics, Division of Psychological Medicine and Clinical Neurosciences, Cardiff University, Cardiff, Wales UK; 31 The Cardiff University Brain Research Imaging Centre (CUBRIC), Cardiff University, Cardiff, Wales UK; 32 Department of Psychiatry, Beth Israel Deaconess Medical Center, Harvard Medical School, Boston, MA USA; 33 Centre for Neuroimaging and Cognitive Genomics (NICOG), Clinical Neuroimaging Laboratory, NCBES Galway Neuroscience Centre, National University of Ireland Galway, Galway, Ireland; 34 Department of Psychiatry, Royal College of Surgeons in Ireland, Dublin, Ireland; 35 Departments of Neurology, Psychiatry, Radiology, Engineering, Pediatrics and Ophthalmology, University of Southern California, Los Angeles, CA USA

**Keywords:** Schizophrenia, Neuroscience, Genetics, Diagnostic markers

## Abstract

22q11.2 deletion syndrome (22q11DS)—a neurodevelopmental condition caused by a hemizygous deletion on chromosome 22—is associated with an elevated risk of psychosis and other developmental brain disorders. Prior single-site diffusion magnetic resonance imaging (dMRI) studies have reported altered white matter (WM) microstructure in 22q11DS, but small samples and variable methods have led to contradictory results. Here we present the largest study ever conducted of dMRI-derived measures of WM microstructure in 22q11DS (334 22q11.2 deletion carriers and 260 healthy age- and sex-matched controls; age range 6–52 years). Using harmonization protocols developed by the ENIGMA-DTI working group, we identified widespread reductions in mean, axial and radial diffusivities in 22q11DS, most pronounced in regions with major cortico-cortical and cortico-thalamic fibers: the corona radiata, corpus callosum, superior longitudinal fasciculus, posterior thalamic radiations, and sagittal stratum (Cohen’s *d*’s ranging from −0.9 to −1.3). Only the posterior limb of the internal capsule (IC), comprised primarily of corticofugal fibers, showed higher axial diffusivity in 22q11DS. 22q11DS patients showed higher mean fractional anisotropy (FA) in callosal and projection fibers (IC and corona radiata) relative to controls, but lower FA than controls in regions with predominantly association fibers. Psychotic illness in 22q11DS was associated with more substantial diffusivity reductions in multiple regions. Overall, these findings indicate large effects of the 22q11.2 deletion on WM microstructure, especially in major cortico-cortical connections. Taken together with findings from animal models, this pattern of abnormalities may reflect disrupted neurogenesis of projection neurons in outer cortical layers.

## Introduction

22q11.2 deletion syndrome (22q11DS; also known as Velocardiofacial or DiGeorge syndrome) results from a recurrent 1.5–3 megabase (Mb) microdeletion on the long arm of chromosome 22. It is the most common chromosomal microdeletion syndrome, with a prevalence of 1 per 3000 to 4000 live births [1, 2]. 22q11DS is associated with a range of characteristic abnormalities, including cardiac defects, craniofacial anomalies, and intellectual disability [1, 3]. Particularly, it increases the risk for psychotic illness around 25-fold relative to the general population [2, 4–6]. The deletion is also associated with elevated rates of other developmental neuropsychiatric disorders [5], but the increased risk for psychosis in 22q11DS may be the most specific association, as it greatly exceeds the roughly threefold increased risk of psychosis associated with general developmental delay [7, 8]. Notably, mouse models of the 22q11.2 deletion show fewer neural progenitors of projection neurons in cortical layers 2/3, which leads to altered connectivity between cortical association areas [9]. Hence, 22q11DS is a compelling model to study genetic causes and neural mechanisms underlying disorders of cortical circuit development, such as schizophrenia.

WM microstructural properties can be quantified noninvasively using diffusion magnetic resonance imaging (dMRI). Fractional anisotropy (FA), a widely used measure of white matter (WM) microstructural organization, is derived from a common dMRI reconstruction method, diffusion tensor imaging (DTI), and may reflect the coherence and density of fiber tracts in a voxel. Other DTI indices, axial diffusivity (AD) and radial diffusivity (RD), are also altered in a range of brain diseases [10]. For example, lower AD can reflect axonal damage and degeneration [11], or smaller axonal diameter [12]. RD is associated with inter-axonal spacing (i.e., extracellular space) [12]; in animal models, demyelination and dysmyelination can lead to abnormally high RD [13–15]. Mean diffusivity (MD) is a generalized measure of the surface-to-volume ratio of cellular membranes [16].

Disturbances in WM microstructural organization have been frequently reported in 22q11DS; however, studies to date have been relatively small, with highly variable findings. While many studies reported lower FA in 22q11DS compared to healthy controls (HC) in major WM tracts, including commissural, association and projection fibers [17–22], several others reported higher overall FA [23–25], or mixed findings across tracts [26–31]. Most studies reported consistent decreases in DTI-derived diffusivity measures (i.e., MD, RD, and AD), although some report mixed results [20] or higher WM diffusivity in 22q11DS [21, 22]. Supplementary Table S1 summarizes prior findings. These contrasting reports have hindered conclusions regarding the nature of WM microstructural abnormalities in 22q11DS.

Contrasting findings in prior studies may also be due to different analytical techniques, ranging from tract-based spatial statistics (TBSS [32]) to voxel-wise analyses and tractometry. This technical variability makes it difficult to apply traditional meta-analytic approaches that attempt to combine summary statistics from prior publications.

WM differences associated with psychosis are of interest in 22q11DS. Psychotic symptoms in 22q11DS have been associated with higher FA and lower WM diffusivities, but not always in the same regions across studies [22, 25, 30, 31, 33, 34]. In addition, there is variability in deletion breakpoints; 85–90% of individuals with the deletion have a ~3 Mb (A–D) deletion, containing 46 protein-coding genes, whereas ~10% of cases have a nested 1.5 Mb (A–B) deletion [1]. WM differences in 22q11DS may be due in part to variable deletion size, as deletion size impacts cortical surface area [35].

To address these uncertainties and determine factors that affect WM abnormalities in 22q11DS, the 22q11DS Working Group of the Enhancing Neuroimaging Genetics through Meta-Analysis Consortium (ENIGMA-22q11DS) performed a coordinated analysis of the raw dMRI data from ten independent studies, and meta-analyzed group differences and their modulators. We addressed these questions:

1. Are there consistent group differences in WM microstructure between 22q11.2 deletion carriers and demographically matched HC?
2. Are there differential age effects between groups, suggesting altered WM development in 22q11DS?
3. Do 22q11DS participants with a psychotic disorder show more severe WM alterations, and do these differences overlap with those found in idiopathic schizophrenia?
4. Does deletion size impact DTI indices?
5. Is WM microstructure related to cognitive abilities, in 22q11DS and in HC?

## Methods

### Participants

dMRI data were contributed from ten previously collected studies to be analyzed as part of the ENIGMA-22q11DS working group. This analysis included 594 participants: 334 with 22q11DS (mean age: 16.88 ± 6.43, 153 females) and 260 HC (mean age: 16.55 ± 8.01, 123 females). Demographic characteristics are shown in Table 1 and Supplemental Table S2a, b. Psychotropic medication status at the time of scanning is included in Supplementary Table S2c. Individual study details are in Supplemental Table S3. Institutional review boards at participating institutions approved all study procedures, and material transfer agreements approved any sharing of de-identified imaging data. A written informed consent was obtained from all study participants or a legal guardian.

**Table 1:** Demographic information of study participants

	Healthy controls (HC)				22q11.2 DS				
Site	*N*	*N* (% by sex)	Mean age (SD)	Mean IQ (SD)	*N*	*N* (% by sex)	Mean age (SD)	Mean IQ (SD)	Group differences
**UPenn**	49	30 (61.2%) M; 19 (38.8%) F	17.31 (3.22)	–	43	26 (60%) M; 17 (40%) F	17.49 (3.13)	77.16 (10.96)	Age: *t* *=* 0.27 (*p* = 0.79) Sex: *X*^*2*^ = 0.01 (*p* = 0.94) IQ: NA
**UCLA**	32	16 (50%) M; 16 (50%) F	12.59 (5.62)	111.97 (21.69)	49	25 (51%) M; 24 (49%) F	14.69 (5.59)	76.55 (12.61)	Age: *t* = 1.60 (*p* = 0.11) Sex: *X*^*2*^ = 0.02 (*p* = 0.89) IQ: *t* = −9.11 (*p* < 0.0005)
**SUNY Upstate**	11	5 (45.45%) M; 6 (54.5%) F	21.12 (2.01)	87.77 (16.25)	34	19 (55.8%) M; 15 (44.11%) F	20.85 (1.86)	78.31 (13.72)	Age: *t* = −0.43 (*p* = 0.67) Sex: *X*^*2*^ = 0.49 (*p* = 0.48) IQ: *t* = −1.99 (*p* = 0.06)
**University of Newcastle**	17	8 (47.1%) M; 9 (52.9%) F	17.06 (3.01)	106.63 (17.58)	16	6 (37.5%) M; 10 (62.5%) F	16.63 (2.75)	72.63 (13.45)	Age: *t* = −0.43 (*p* = 0.67) Sex: *X*^2^ = 0.31 (*p* = 0.58) IQ: *t* = −6.14 (*p* < .0005)
**Maastricht University**	36	23 (63.8%) M; 13 (36.2%) F	29.97 (10.05)	105.13 (14.13)	24	11 (45.9%) M; 13 (54.1%) F	30.05 (7.86)	74.42 (9.76)	Age: *t* = 0.04 (*p* = 0.97) Sex: *X*^*2*^ = 1.91 (*p* = 0.17) IQ: *t* = −8.14 (*p* < .0005)
**Institute of Psychiatry London**	24	10 (41.7) % M; 14 (58.3%) F	18.36 (6.73)	115.92 (15.03)	24	13 (54.1%) M; 11 (45.9%) F	18.04 (6.88)	84.46 (14.15)	Age: *t* = −0.16 (*p* = 0.87) Sex: *X*^*2*^ = 0.75 (*p* = 0.39) IQ: *t* = −7.47 (*p* < .0005)
**UC Davis #1**	36	19 (52.7%) M; 17 (47.3%) F	10.22 (2.38)	116.06 (10.69)	31	16 (51.6%) M; 15 (48.4%) F	10.86 (2.14)	73.60 (14.41)	Age: *t* = 1.14 (*p* = 0.26) Sex: *X*^*2* = ^0.01 (*p* = 0.92) IQ: *t* = −13.23 (*p* < .0005)
**UC Davis #2**	41	20 (48.8%) M; 21 (51.2%) F	11.05 (2.33)	115.16 (15.75)	46	21 (45.7%) M; 25 (54.3%) F	11.64 (2.53)	74.76 (13.83)	Age: *t* = 1.11 (*p* = 027) Sex: *X*^*2*^ = 0.09 (*p* = 0.77) IQ: *t* = −12.45 (*p* < .0005)
**Cardiff**	14	6 (42.9%) M; 8 (57.1%) F	14.46 (1.79)	105.25 (9.55)	13	6 (46.2%) M; 7 (53.8%) F	16.03 (4.63)	80.92 (19.30)	Age: *t* = 1.18 (*p* = 0.25) Sex: *X*^*2*^ = 0.03 (*p* = 0.86) IQ: *t* = −3.91 (*p* = 0.001)
**Utrecht**	–	–	–	–	54	38 (70.3%) M; 16 (29.7%) F	17.52 (4.22)	69.24 (7.66)	NA
**Total**	260	137 (52.6%) M; 123 (47.3%) F	16.55 (8.01)	111.62 (16.16)	334	181 (54.1%) M; 153 (45.8%) F	16.88 (6.43)	75.14 (12.79)	Age: *t* = 0.55 (*p* *=* 0.57) Sex: *X*^*2*^ = 0.04 (*p* = 0.5) IQ: *t* = 25.9 (*p* = 4.0e−79)

Demographic information of study participants, per site. (1) University of Pennsylvania/Children’s Hospital of Philadelphia (PA, USA); (2) University of California Los Angeles (CA, USA); (3) State University New York Upstate (NY, USA); (4) University of Newcastle (NSW, Australia); (5) Maastricht University (Netherlands); (6) Institute of Psychiatry (London, UK); (7) University of California Davis #1 (CA, USA); (8) University of California Davis #2 (CA, USA); (9) Cardiff Univ. (WAL, UK); (10) Utrecht Univ. (The Netherlands)

*SD*  standard deviation, *M*  Male, *F*  Female, *HC*  healthy controls

### Measurements of sample-specific phenotype characteristics

All sites conducted structured diagnostic interviews at the time of scanning to determine lifetime psychiatric diagnoses. Wechsler IQ assessments were used to assess cognitive function (Supplemental Table S3, Supplemental Methods 1).

Across sites, deletion size was determined using multiplex ligation-dependent probe amplification (MLPA) [36]. The large sample size here uniquely allowed for the comparison of effects of the two most frequent deletion types, the longer A–D vs the shorter A–B deletion, on DTI measures. From cases with available MLPA data, 206 subjects had the A–D deletion (89.9%), and 15 (6.5%) subjects had the A–B deletion (see Supplemental Table S3).

### Image acquisition and processing

**Fig. 1:**
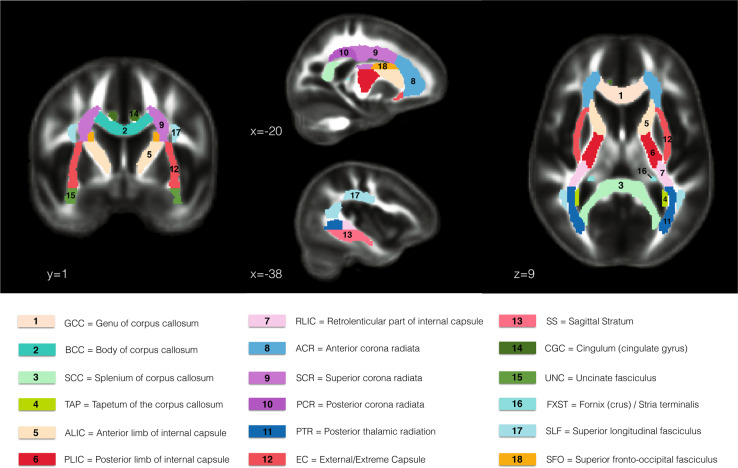
Depiction of the 18 regions of interest (ROIs) of the Johns Hopkins University (JHU-ICBM) white matter atlas [39] that were analyzed in the present study

Acquisition parameters of dMRI and T1-weighted MRI scans for each site are shown in Supplemental Tables S4 and S5. All raw data were preprocessed in an identical fashion at a single site (Supplemental Methods 2). FA, MD, RD, and AD maps were skeletonized as described in the ENIGMA-DTI protocol [37, 38], based on the TBSS method [32], ensuring that all data are normalized to the ENIGMA-DTI template. Mean values were calculated for each DTI measure along the skeleton within each ROI defined by the Johns Hopkins University WM atlas (JHU-ICBM-DTI-81) distributed by FSL [37, 39]. For all analyses, we used the mean of the right and left values for bilateral ROIs, for each measure; we included the mean of all WM JHU-ICBM ROIs and we excluded the corticospinal tract, midsagittal fornix region and the hippocampal portion of the cingulum bundle as these ROIs are difficult to reliably register, or were subject to artifacts in cohorts in this study [40]. The ROIs included are shown in Fig. 1.

### Statistical analyses

#### Effects of 22q11DS and age on DTI-derived measures

Group differences between 22q11DS and HC were investigated using two analytic approaches: a *meta-analysis*, which runs statistical comparisons for each site separately and combines the summary statistics across sites, and a *mega-analysis*, in which data are harmonized and pooled from individual subjects, and statistical analysis is run on the full group. The meta-analysis included 540 subjects: 278 22q11DS probands (mean age: 16.76 ± 6.78, 138 females) and 260 HC (mean age: 16.55 ± 8.01, 123 females) from nine independent datasets derived from eight sites (Table 1). Because Utrecht included only 22q11DS cases, it was not included in the case-control analyses. For each site, linear regressions were run, in which the mean DTI measure for each ROI was the dependent variable, diagnosis was the predictor of interest, and age, [age-mean(age)]^2^ and sex were included as covariates. Given that DTI-derived measures tend to peak between 11 and 20 years for commissural and association fibers and in the early twenties for projection fibers [41, 42], we included both the linear and quadratic effects of age in the model. The quadratic age term was centered to avoid collinearities with the linear age term. In addition, because females and males show different trajectories of DTI measures across development [43], sex was accounted for in the model. Cohen’s *d* effect sizes for diagnosis were computed. Subsequently, an inverse-variance weighted mixed-effect meta-analysis [44] to combine individual site effect sizes, as in [40].

A pooled, or mega-analytic, approach was also conducted. As multiple factors can affect the distribution of DTI measures [45–47], additional harmonization of DTI measures can be advantageous when conducting studies pooling dMRI data from different protocols. We used the COMBAT algorithm [48] to harmonize data across sites for each DTI measure (FA, MD, RD, and AD) for each WM ROI. This algorithm uses an empirical Bayes framework to estimate additive and multiplicative site effects. It has been used previously for harmonization of multisite DTI data, and has been shown to perform better than several other methods for modeling and removing inter-site variability [48]. Next, group differences were assessed using the same model tested in the meta-analysis. Finally, the diagnosis-by-age interaction effect term was included in the mega-analytic model to test whether effects of age differed in 22q11DS probands relative to HC.

We used the Benjamini & Hochberg method to control for the family wise error rate [49]. The percentage of tolerated false positives was 5% (*q* < 0.05). Critical *p*-values were calculated for each set of models, specifically: (1) meta-analysis; (2) mega-analysis; and (3) mega-analysis including diagnosis-by-age interaction. Effect sizes were derived as explained in Supplemental Methods 3.

In addition, given previous findings of nonlinear trajectories of DTI-derived measures with respect to age in healthy individuals (5–82 years) [50], we fit a Poisson nonlinear model for age for each group separately (HC and 22q11DS) for each WM ROI and for each DTI-derived measure, to further investigate age effects. We used the previously harmonized data (see above COMBAT harmonization), to reduce site effects. We measured the age of peak FA and age of minimum MD, RD, and AD as in Lebel et al. [50] and compared both groups using a two-tailed *t*-test for means with outlier removal (*α* = 0.05). Thereafter, we calculated the percent change of each DTI measure for each ROI from age 6 (minimum age in both groups) to peak/minimum, and from peak/minimum to age 46 and 52 (maximum age for 22q11DS and HC, respectively). We compared the percent changes of each DTI measure for all ROIs between 22q11DS and HC groups by using Yuen’s method with bootstrap-t for trimmed means (*α* = 0.05) [51].

#### Influence of psychotic disorder, deletion size, and IQ on DTI measures

To assess potential differences in WM architecture as a function of clinical and genetic variability, we examined the effects of psychotic illness (35 with psychotic disorder vs 191 without psychosis) and deletion size (206 AD vs 15 AB) on DTI measures, within individuals with 22q11DS. In addition, given that IQ is a group-associated variable, we examined partial correlations with IQ within the 22q11DS (*N* = 304) and HC groups (*N* = 102) separately. For these analyses the DTI measures for each ROI were included as dependent variables. Age, [age-mean(age)]^2^ and sex were included as covariates. FDR correction was performed as specified above (Section “Effects of 22q11DS and age on DTI-derived measures”).

 There is a strong association between age and psychosis onset [5], and there was a significant difference in mean age between 22q11DS cases with and without psychosis (see Supplementary Table S2b). To assess the effect of psychosis within the 22q11DS group, we used a local nonparametric ANCOVA method [51] covarying for age (see Supplemental Methods 4). This approach allowed for a controlled test within age subgroups.

Next, in order to determine whether the microstructural differences observed in 22q11DS-associated psychosis overlap with those seen in idiopathic schizophrenia, we compared our results for 22q11DS cases with and without psychosis to schizophrenia case-control results from the ENIGMA-Schizophrenia DTI Working Group [40], analyzed using the same protocols as in our study.

## Results

### Group differences across sites

**Fig. 2:**
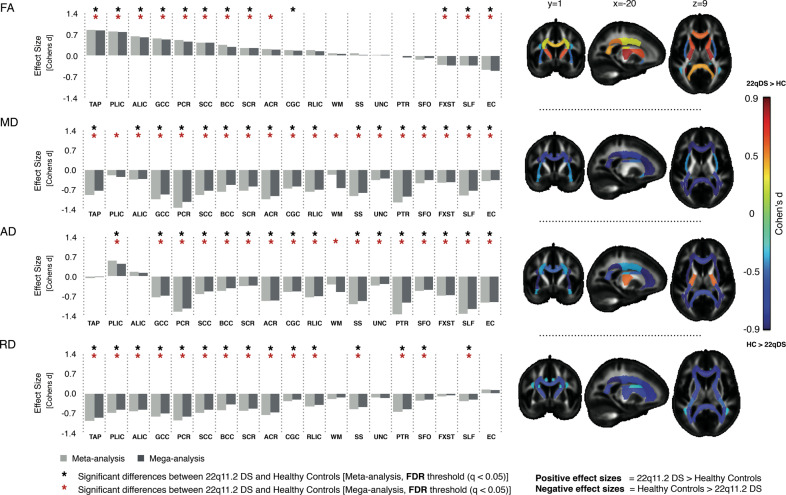
Results of meta- and mega-analyses including nine independent datasets from the ENIGMA-22q11DS working group. The bar graphs on the left side are organized based on the effect sizes for FA (positive to negative, from left to right). The brain maps on the right side are organized by rows, each one corresponding to respective bar graph on the left. These show the JHU-ICBM atlas white matter ROIs that passed multiple comparisons correction after meta-analysis. The model tested was: DTI-ROI-measure = *ß*_0_ *+* *ß*_1_Diagnosis *+* *ß*_2_Sex *+* *ß*_*3*_Age *+* *ß*_4_Age^2^_centered_. WM: Average of all white matter JHU-ICBM ROIs

We first investigated whether there were consistent group differences in WM microstructure between 22q11.2 deletion carriers and HC, using a standardized processing pipeline. Equally important is to determine whether harmonization of the data would allow pooled analyses for further investigation of modulatory factors (psychosis, deletion size, and IQ). Figure 2 shows group differences for 22q11DS cases vs HC, from the meta-analysis and mega-analysis: results were nearly identical, with similar effect sizes. Effect sizes for each site are shown in Supplementary Fig. 1. Most ROIs that significantly differed between 22q11DS and HC showed lower diffusivity values (MD, AD, and RD) in 22q11DS subjects, but a mixed pattern for FA. Significantly higher FA in 22q11DS cases relative to HC was observed in the tapetum (TAP), genu (GCC), body and splenium of the corpus callosum (BCC/SCC), the anterior and posterior limb of the internal capsule (ALIC/PLIC), and posterior and superior corona radiata (PCR/SCR), with moderate to large effect sizes (*d* ~ 0.3–0.8), for both analyses. In contrast, ROIs in association fibers—the superior longitudinal fasciculus (SLF), fornix/stria terminalis (FXST), and external/extreme capsules (EC)—showed significantly lower FA in 22q11DS relative to HC (Supplementary Tables S6 and S7).

22q11DS subjects had significantly lower MD than HC in almost all ROIs investigated, with greatest effects (*d* ~ 1.0) in the PCR and posterior thalamic radiation (PTR); both contain mostly thalamo-cortical/cortico-thalamic and corticofugal fibers from posterior brain areas. For all 18 ROIs, MD was lower in 22q11DS, as was AD, for 15 of the 18 ROIs. Only the PLIC showed significantly higher AD in 22q11DS relative to HC. For RD, all ROIs showing significant differences (15 of 18 ROIs) were lower in 22q11DS than HC, with largest effects (*d* ~ 0.7) in the corpus callosum and PCR (Supplementary Tables S6 and S7).

### Age-associated effects

Given the wide age range (6–52 years), we wanted to determine whether the development of WM appears delayed or altered in 22q11DS. As shown in Supplementary Table S6, there were highly significant linear effects of age for all indices for the majority of ROIs. FA was positively associated with age, while the opposite pattern was found for diffusivity values (MD, AD, and RD). There were also significant quadratic effects for almost all ROIs for FA, MD, and RD. AD showed fewer significant quadratic effects, in both the meta- and mega-analyses. However, no significant age-by-diagnosis interactions were observed (Supplementary Table S8). Given the sparse representation of older adults, we also performed a mega-analysis with a subsample of subjects under 30 years old to explore potential age-by-diagnosis effects, which yielded similar results (Supplementary Table S9).

We also investigated Poisson regression models to further evaluate effects of age on WM development. These models did not provide a substantially better fit to the data than the linear regression model used above, as determined by the residual standard error of the fits (see Supplementary Tables S10–S12). As such, we retained the linear regression models for our primary analyses, but report the additional trajectory information obtained from the Poisson models below.

Scatterplots for the nonlinear Poisson fits of age per ROI for each DTI-derived measure are displayed in Supplementary Figs. S2–S5. There were fewer ROIs with significant peak/minimum estimates in the 22q11DS group, across all DTI indices (see Supplementary Tables S13 and S14). Generally, those ROIs without significant peak/minimum estimates have linear rather than exponential growth and decay trajectories. When comparing the mean age of peak FA (across ROIs) between HC and 22q11DS, average peak FA was significantly older in 22q11DS. We found a significantly older mean age at minimum RD in 22q11DS, but no differences in mean ages at minimum MD and AD. The mean percent change of FA after its peak and mean percent change of  RD and MD after their minima were also significantly greater in HC vs 22q11DS, with no differences in AD (Supplementary Table S15).

### Influence of psychosis

**Fig. 3:**
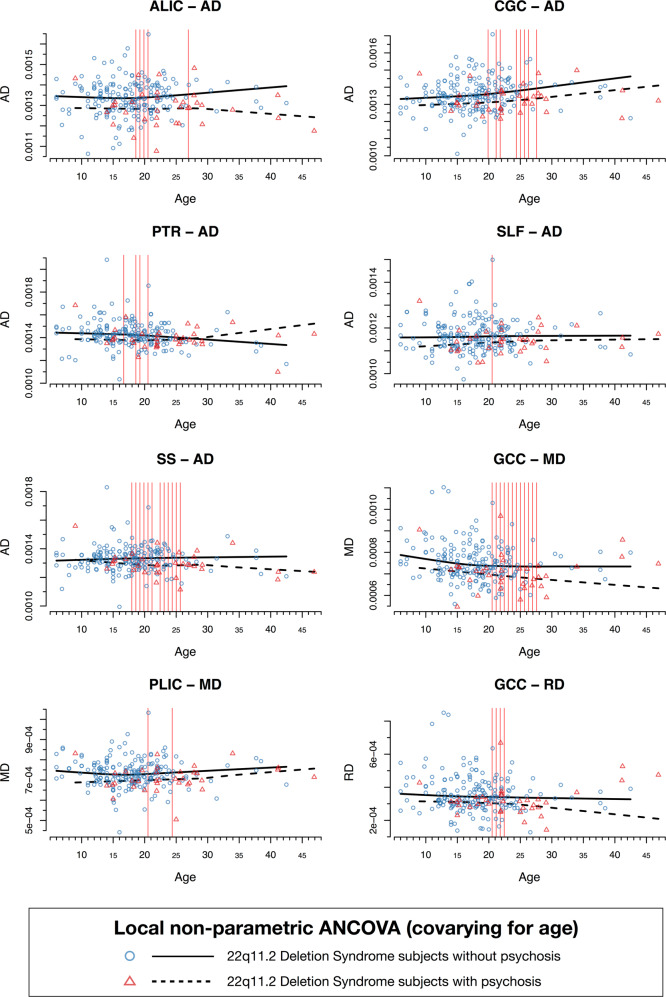
Results from the local nonparametric ANCOVA analysis comparing 22q11DS subjects with psychotic disorder (*N* = 35) vs those with no lifetime history of psychotic symptoms (*N* = 191). Shown here are the results for DTI indices that significantly differed between 22q-Psychosis vs 22q-No Psychosis: AD in the ALIC, CGC, PTR, SLF, and SS, RD in GCC, and MD in the GCC and PLIC. All analyses were performed on 25 design points corresponding to different age bands. Vertical red lines correspond to the ages at which these DTI measures (AD, MD, and RD) significantly differed between subjects with 22q11DS with and without psychosis (Supplementary Table S16)

Are the deletion-related WM changes more severe in those with psychotic disorder? Relative to 22q11DS subjects without psychosis, 22q11DS subjects with psychotic disorder showed overall lower diffusivity values, with significantly lower AD in the ALIC and PTR, both predominantly containing thalamic radiation fibers, in the cingulum of the cingulate gyrus (CGC) and the SLF, which mostly contain fronto-parietal and fronto-temporal association fibers, and the sagittal stratum (SS), which contains both posterior thalamic projection and temporal association fibers. 22q11DS-Psychosis was also associated with significantly lower RD and MD in the GCC, which contains callosal fibers, and significantly lower MD in the PLIC, where the superior thalamic radiation and cortico-pontine fibers are the major constituents. These differences were seen primarily between ages 20 and 26 for most ROIs; some ROIs (ALIC, PTR, and SS) showed differences by age 16–17 (Fig. 3 and Supplementary Table S16**)**. Overall, these findings confirm that WM differences detected by DTI diffusivity measures are more severe in 22q11DS patients with psychotic disorder, and are particularly evident in young adulthood.

### Comparison of WM microstructure in 22q11DS-psychosis to idiopathic schizophrenia

**Fig. 4:**
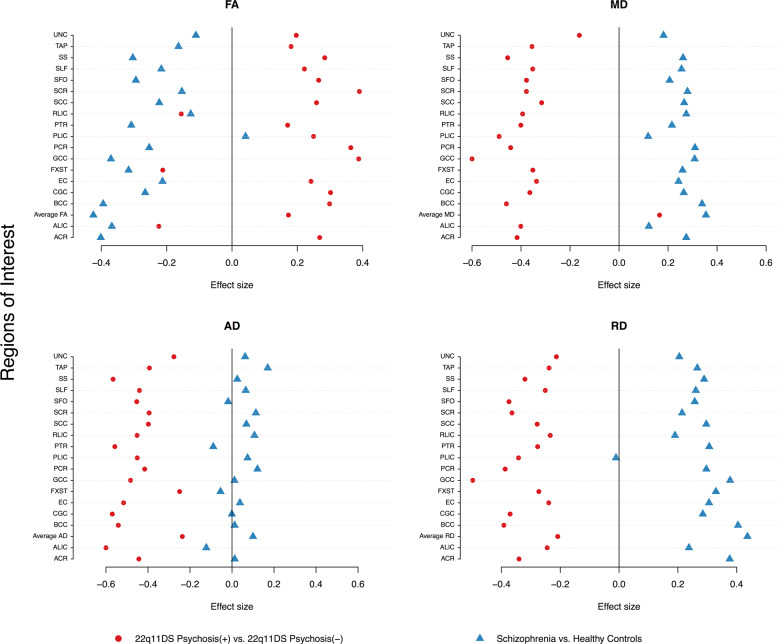
Comparison of effect sizes in this study, to those from the ENIGMA-Schizophrenia DTI Working Group using similar methods (2359 healthy controls vs 1963 schizophrenia patients from 29 independent studies; Kelly et al. [40]; blue triangles) to 22q11DS probands with and without psychosis (red circles). Positive effect sizes: 22q-Psychosis > 22q-No psychosis OR schizophrenia patients > healthy controls. Negative effect sizes: 22q-No psychosis > 22q-psychosis OR healthy controls > schizophrenia patients. We note, as stated in the ENIGMA-DTI protocol [38], that the IFO and UNC in the original JHU atlas from FSL, were later renamed the UNC and TAP, respectively. Here we matched the ENIGMA-Schizophrenia results with the updated atlas

Next, we compared our results for 22q11DS cases with and without psychosis to schizophrenia case-control results (2359 HC vs 1963 schizophrenia patients) [40], plotted together for visualization purposes (Fig. 4). Effects for 22q11DS cases with and without psychosis differed markedly from those observed for idiopathic schizophrenia relative to HC. Specifically, while patients with 22q11DS-psychosis tended toward higher FA and lower diffusivity values compared to 22q11DS individuals without psychosis, patients with idiopathic schizophrenia showed overall lower FA across tracts and increased diffusivity values relative to HC, particularly for MD and RD.

### Influence of deletion type and IQ

Does the extent of the deletion affect WM microstructure? Subjects with the large A–D deletion showed a trend toward lower AD in the anterior corona radiata and EC, and higher FA in the TAP; however, there were no statistically significant differences in relation to deletion size, after multiple comparisons correction (see Supplementary Fig. S6 and Supplementary Table S17).

In addition, regarding relationships between DTI indices and cognitive abilities, HC showed trends toward positive correlations of MD, RD, and AD in multiple ROIs with IQ, and a trend toward a negative correlation of FA with IQ in the TAP. Within 22q11DS cases, findings were similar, but higher IQ was associated with significantly higher AD in the PTR, which contains mainly posterior cortico-thalamic and thalamo-cortical fibers. There was also a trend toward higher AD in the average WM, genu of the CC, and SS being associated with higher IQ in 22q11DS (Supplementary Fig. S7, Supplementary Table S18). While these relationships were not significant when corrected for multiple comparisons, the overall pattern of findings suggests that relationships between WM microstructure and cognition need further investigation in 22q11DS relative to typically developing controls.

## Discussion

This is the largest study to date of WM microstructure in 22q11DS (334 22q11DS cases and 260 HC), assessed by DTI. Our analysis pipeline [37, 40] allowed for coordinated prospective meta- and mega-analyses of the data across sites, unlike traditional meta-analyses that combine statistical results from the literature. This approach addresses, for the first time, issues of low power due to small sample sizes and variable analysis protocols that contribute to heterogeneity and lack of clarity in DTI studies to date.

In contrast to findings in many neuropsychiatric disorders [40, 52], our findings revealed overall lower DTI diffusivities (AD, RD, and MD) in 22q11DS compared to HC, with regionally varying directions of effect for FA. Higher FA, lower RD and AD (and consequently, lower MD) appear to be the hallmark of microstructural alterations in the major WM tracts in 22q11DS, especially in the commissural fibers of the corpus callosum. While this may suggest greater myelination [13], we must be cautious in applying this interpretation to our findings, given that dMRI cannot directly index the degree of myelination [53]. Anisotropy does not only depend on the presence of myelin in the WM, as it has been demonstrated in unmyelinated tracts [54] and is also sensitive to axonal density. RD is sensitive to axonal density and amount of extracellular space, and AD to axonal diameter and organization [12, 55]. Moreover, since axonal density and myelination are correlated [54, 56], it is not possible to disentangle one from another when interpreting FA and RD differences between populations. We postulate that the observed group differences may result from an increase in the cumulative cellular membrane circumference [57] in 22q11DS (attributable to differences in axon composition, myelination and/or reactive astrocytes), which hinders diffusion perpendicularly to the WM tracts, hence increasing anisotropy and decreasing RD.

Our findings of higher FA in 22q11DS relative to controls in ROIs in commissural tracts (TAP, GCC, BCC, and SCC), no detectable differences in ROIS where projection fibers predominate (RLIC, SS, PTR, and SFO), and lower FA in ROIs in long association tracts (EC, SLF, FXST) are consistent with findings in the mouse model of 22q11DS [9]. Specifically, this study found that proliferation of basal, but not apical progenitors is disrupted, and subsequently the frequency of projection neurons in layers 2/3, but not layers 5/6, is altered. Commissural and long association fibers originate primarily from projection neurons, i.e., pyramidal neurons in the outer layers 2/3, whereas corticofugal and cortico-thalamic projection fibers tend to originate from pyramidal cells in cortical layers 5/6. Moreover, our results suggest that the nature of WM disruptions may differ between callosal and long association fibers in 22q11DS, but advanced microstructural MRI techniques may be necessary to disentangle these differences. As such, these cross-species findings collectively suggest a potential neurobiological model in which haploinsufficiency at the 22q11.2 locus leads to disruptions of specific aspects of early brain development, and subsequent changes in neural circuitry that likely elevate risk for neuropsychiatric disorders in 22q11DS patients.

We speculate that our findings may be related to three types of histopathological alterations in the WM of 22q11DS patients, all of which could reduce diffusivity. First, a recent neuropathology study of a 3-month-old infant with 22q11DS reported decreased neuronal frequencies in outer cortical layers and increased neuronal frequencies in deeper cortical layers [9]. This is closely related to findings in the *LgDel* 22q11.2 mouse model mentioned above [9]. Pyramidal neurons of cortical layers 2/3 generate a substantial portion of the cortico-cortical axonal projections between association areas [58]. These axons are present in most of the WM ROIs included in this study. Consequently, target-to-origin signaling between cortical association areas (cortico-cortical projections) may be disrupted in 22q11DS, affecting the necessary cues to initiate proper axonal differentiation [59, 60], ultimately affecting the development of a typical distribution of axonal diameters [61–63], and therefore altering RD and AD in WM bundles. Moreover, the PLIC was the only ROI showing higher AD in 22q11DS. AD has been associated with axonal diameter changes and axonal tortuosity in rats [12, 55]. PLIC is the only ROI in this study that contains mostly corticofugal fibers, which primarily derive from cortical layers 5/6 [39, 58], suggesting that the axonal size distribution within fiber bundles originating in the deeper cortical layers may differ from those originating in the outer cortical layers [61, 63]. Further studies of animal models and postmortem human brain tissue may shed light on this.

Second, DTI abnormalities may also reflect gliotic changes secondary to microvascular insults. Postmortem findings in 22q11DS adults indicate both deep WM gliosis associated with cerebrovascular changes [64]. Gliosis—occurring as brain reacts to microvascular injuries—has been associated with increased anisotropy in a mouse brain injury model [65]. Third, DTI measures may be affected by ectopic neurons in WM that may result from neuronal migration defects during early development [66]. These have been reported in both neuropathologic [64, 67, 68] and neuroimaging studies of 22q11DS patients [69, 70]. While we did not detect any heterotopias in our cohort, subtle microscopic ones may be detected only via histology.

The age trajectories of FA, MD, RD, and AD, as well as peak and minimum age estimates of our control sample, were similar to those reported previously [50]. However, 22q11DS patients showed an older mean age of both peak FA and minimum RD; correspondingly, they also showed smaller percent changes for FA and MD after peak and minimum ages, respectively. As noted above, these findings may indicate a delay in maturation secondary to altered axonal diameters and organization in the deep WM, which could be precursors of a delayed myelination process. Conversely, a smaller percent change after maturation (indicated by peak FA and minimum RD) may be indicative of underlying organizational changes in WM that abnormally hinder diffusion and may result from gliotic changes, as has been reported in adult post-mortem 22q11DS brain tissue [64]. Nevertheless, despite the harmonization protocol interpretive caution is warranted because the age distribution was variable across sites and data points were rather sparse in the older age ranges.

Consistent with some single-site studies suggesting inverse correlations between psychotic symptom severity in 22q11DS and diffusivity in the CC and long association tracts [25, 29–31, 33], we found lower RD and MD in those with psychosis in the genu of the CC, and lower AD in long association tracts such as the SLF and CGC. Interestingly, significantly lower AD was found in ROIs with predominantly cortico-thalamic and thalamo-cortical fibers such as the ALIC, SS and the PTR. A previous single-site tractometry study found significant associations between higher FA and lower RD in the ALIC with positive prodromal symptoms [29]. Future studies should prospectively investigate the role of the major thalamic projection tracts in the emergence and progression of psychotic symptoms in 22q11DS.

Notably, WM microstructural alterations in 22q11DS with psychosis showed a largely opposite pattern from those seen in idiopathic schizophrenia, involving primarily FA being higher (rather than lower), and lower (rather than higher) diffusivity measures. A previous single site study of 22q11DS and youth at clinical high risk for psychosis reported this directionally opposite pattern as well [24]. This is in contrast to findings for cortical gray matter, in which 22q11DS patients with psychosis showed highly significant overlap with idiopathic schizophrenia, in terms of prominent cortical thinning in fronto-temporal regions [35]. Thus, our findings suggest that patterns of neuroanatomic overlap in 22q11DS-associated vs. idiopathic psychosis markedly differ for gray and WM, and suggest that different WM phenotypes may lead to similar downstream clinical outcomes. Our findings of altered AD in 22q11DS, more extreme in those with psychosis, may indicate altered axonal diameter and increased tortuosity of WM tracts [12, 55]. Numerous smaller, tortuous axons in key connections between cortical association areas may lead to altered WM maturation, structural dysconnectivity and possibly psychosis. In idiopathic schizophrenia, WM degeneration (demyelination and loss of axons with larger diameters) may also lead to disrupted axonal morphology that similarly results in structural dysconnectivity between cortical association areas.

We did not find consistent effects of deletion size on WM architecture, and found little evidence that the relationship between WM microstructure and IQ differed between 22q11DS cases and HC. Sample size was quite limited for the A–B deletion type, and imaging protocols varied across sites, which may have affected our results. In addition, given highly variable psychotropic medications and medical comorbidities in 22q11DS patients, their effects could not be systematically investigated here. Previously, in a sample including many of the same participants as in the current analysis, we found that psychotropic medication was not significantly associated with cortical thickness or cortical surface area in 22q11DS patients [35]. Additionally, prior studies of patients with idiopathic schizophrenia found that WM changes detected by DTI were not attributable to antipsychotic medication [40, 71].

Future studies with multishell acquisitions and novel biophysical models may resolve the contribution of the intra- and extra-axonal volume fractions and axonal diameters to these abnormalities [72, 73]. Quantitative magnetization transfer [74] and perfusion MRI acquisitions [75] may help clarify any myelin abnormalities or underlying brain microvascular pathology in 22q11DS.

Collectively, our findings indicate large effects of the 22q11.2 deletion on WM microstructure. Diffusivity was more consistently affected than FA. In animal models, disruptions to predominantly cortico-cortical and cortico-thalamic/thalamo-cortical connections in 22q11DS may be attributable to disrupted early neurogenesis. Future translational studies will help to determine the neurobiological underpinnings of these alterations.

## Supplementary information

Supplementary Methods

Supplementary Tables

Supplementary Figures
