# Supplementary material for: Altered white matter microstructure in 22q11.2 deletion syndrome: a multisite diffusion tensor imaging study
Source: Mol Psychiatry. 2019 Jul 29;25(11):2818–31. doi: 10.1038/s41380-019-0450-0 (PMC6986984; doi:10.1038/s41380-019-0450-0)
Supplement: Supplementary file 1 — Supplementary Methods [file 41380_2019_450_MOESM1_ESM.pdf]

Supplemental Methods Section 1. Study Participant Ascertainment and Assessment Procedures

Across sites, all cases received a molecularly confirmed diagnosis of 22q11.2 deletion. All 22q11DS subjects included in the psychotic disorder group had a DSM schizophrenia spectrum psychotic disorder diagnosis (schizophrenia, schizoaffective disorder, or psychosis not otherwise specified), as determined via structured diagnostic interview conducted by a trained mental health professional at each site, and supplemented by collateral information and medical records (see **Supplementary Table S3** for details regarding study instruments and study inclusion/exclusion criteria). A cross-site reliability procedure was also undertaken, in which two investigators with clinical expertise independently reviewed a subset of representative cases from each site. References in **Supplementary Table S3** provide additional detail regarding clinical characteristics of each study sample.

Supplemental Methods Section 2. Imaging data preprocessing

We denoised all dMRI images with the LPCA tool (1) and all volumes were skullstriped using FSL's BET tool (2). Eddy correction was performed with FSL's *eddy_correct* tool on all sites but Utrecht. T1-weighted images were bias field corrected with ANTs' N4, denoised with the non-local means algorithm (3,4) and skull-striped with Freesurfer (5). Subsequently, the EPI (echo-planar imaging) distortion correction was performed by non-linearly aligning the non-diffusion sensitized volumes (b=0 s/mm^2) to the subjects' corresponding preprocessed T1-weighted image. The non-linear registration was performed with ANTs (6). The deformation fields were applied to all the diffusion sensitized volumes. For the scans from Utrecht, eddy and EPI distortion corrections were performed with FSL's TOPUP and EDDY tools (7). Thereafter, we computed DTI-FA maps which were used to register each subject linearly and nonlinearly to the ENIGMA DTI-FA common template (8). After corroborating the correct alignment of each subject's FA to the ENIGMA DTI-FA atlas, we concatenated the *eddy_correct* linear transformations with the linear transformations and nonlinear deformations to the ENIGMA DTI-FA template. This joint transformation and deformation field was applied to the skullstripped and denoised dMRI. By doing this we ensured that the original dMRI images were interpolated only once to the ENIGMA DTI template. With the dMRI in the ENIGMA template space we calculated the diffusion tensor with a nonlinear fitting and outlier detection for robust estimation (9) by using the DIPY package (10). We computed four scalar maps from the fitted tensors: Fractional Anisotropy (FA), Mean Diffusivity (MD), Radial Diffusivity (RD and Axial Diffusivity (AD). The code for the ENIGMA-DTI protocol is freely available here: http://enigma.ini.usc.edu/ongoing/dti-working-group/.

Supplemental Methods Section 3. Statistical Analyses and Reporting of effect sizes.

As noted in the Methods (2.4), covariates included age, [age-mean(age)]^2 and sex. For three sites (UCLA, Newcastle and Cardiff), an additional term for scanner type was included, as two

scanners were used with the identical acquisition, and a random effect model was performed to take this covariate into account.

Effect sizes for dichotomous variables (diagnosis and deletion type) were computed by converting *t*-values from the multiple linear regressions to *Cohen's d* statistics according to the formula:

$$Cohen's\ d = \frac{2t}{\sqrt{df}}$$

Where *df* is the number of degrees of freedom. For continuous variables (age, IQ), effect sizes were estimated by converting *t*-values from the multiple linear regressions to partial correlations according to the formula:

$$r = \sqrt{\frac{t^2}{t^2 + Res{-}df}},$$

where Res-*df* denotes the residual degrees of freedom.
All statistical analyses were performed with the core statistical R packages for linear regression: https://www.rdocumentation.org/collaborators/name/R-core%20R-core@R-project.org

Supplemental Methods Section 4. Local Nonparametric ANCOVA Statistical Analysis

The local nonlinear ANCOVA makes no parametric assumptions about how two variables are related and is robust to heteroscedasticity (11). It approximates a regression line for each group using a running interval smoother and compares both groups at specific design points. In this case, it selects a specific age as a design point and compares the trimmed means of the DTI-by-ROI measure at all points close to the selected age (proximity calculated by the median absolute deviation (MAD)). If both groups have more than 12 subjects (based on the degrees of freedom required), the regression lines are comparable and a confidence interval is computed followed by a t-test. Dunnett's T3 method is used to control the family-wise error (12). We elected to use this approach for our comparison of 22q11DS cases with and without psychotic disorder, as this method was considered more robust to differences in sample size and mean age between groups (mean 22q11DS+Psychosis=23.87 years; mean 22qDS-No Psychosis= 17.99 years; t=-4.14, p=0.00016). Additionally, age variances were significantly different (F = 0.51899, p=0.006) and non-normal (kurtosis=4.09 and 5.64; skewness=0.93 and 0.94, respectively) which prohibited an ordinary least squares linear regression analysis. An explanatory measure of effect size is also derived from this analysis (13) and reported in **Supplementary Table S10** and **Figure 4.** The code used here for the ANCOVA analysis is freely available at: https://dornsife.usc.edu/labs/rwilcox/software/

Supplementary methods references

1. Manjón JV, Coupé P, Concha L, Buades A, Collins DL, Robles M. Diffusion weighted image denoising using overcomplete local PCA. PLoS One. 2013 Sep 3;8(9):e73021.

2. Smith SM. Fast robust automated brain extraction. Hum Brain Mapp. 2002 Nov;17(3):143–55.

3. Tustison NJ, Avants BB, Cook PA, Zheng Y, Egan A, Yushkevich PA, et al. N4ITK: improved N3 bias correction. IEEE Trans Med Imaging. 2010 Jun;29(6):1310–20.

4. Coupe P, Yger P, Prima S, Hellier P, Kervrann C, Barillot C. An optimized blockwise nonlocal means denoising filter for 3-D magnetic resonance images. IEEE Trans Med Imaging. 2008 Apr;27(4):425–41.

5. Ségonne F, Dale AM, Busa E, Glessner M, Salat D, Hahn HK, et al. A hybrid approach to the skull stripping problem in MRI. Neuroimage. 2004 Jul;22(3):1060–75.

6. Avants BB, Epstein CL, Grossman M, Gee JC. Symmetric diffeomorphic image registration with cross-correlation: evaluating automated labeling of elderly and neurodegenerative brain. Med Image Anal. 2008 Feb;12(1):26–41.

7. Andersson JLR, Sotiropoulos SN. An integrated approach to correction for off-resonance effects and subject movement in diffusion MR imaging. Neuroimage. 2016 Jan 15;125:1063–78.

8. Jahanshad N, Kochunov PV, Sprooten E, Mandl RC, Nichols TE, Almasy L, et al. Multi-site genetic analysis of diffusion images and voxelwise heritability analysis: a pilot project of the ENIGMA-DTI working group. Neuroimage. 2013 Nov 1;81:455–69.

9. Chang L-C, Jones DK, Pierpaoli C. RESTORE: robust estimation of tensors by outlier rejection. Magn Reson Med. 2005 May;53(5):1088–95.

10. DIPY — dipy 0.14.0 documentation [Internet]. [cited 2018 Jul 31]. Available from: http://nipy.org/dipy/

11. Wilcox R. Chapter 11 - More Regression Methods. In: Wilcox R, editor. Introduction to Robust Estimation and Hypothesis Testing (Fourth Edition). Academic Press; 2017. p. 585–691.

12. Dunnett CW. Pairwise Multiple Comparisons in the Unequal Variance Case. J Am Stat Assoc. 1980 Dec;75(372):796–800.

13. Wilcox RR, Tian TS. Measuring effect size: a robust heteroscedastic approach for two or more groups. J Appl Stat. 2011 Jul 1;38(7):1359–68.
