# Supplementary material for: Altered white matter microstructure in 22q11.2 deletion syndrome: a multisite diffusion tensor imaging study
Source: Mol Psychiatry. 2019 Jul 29;25(11):2818–31. doi: 10.1038/s41380-019-0450-0 (PMC6986984; doi:10.1038/s41380-019-0450-0)
Supplement: Supplementary file 2 — Supplementary Tables [file 41380_2019_450_MOESM2_ESM.pdf]

Supplementary tables

Supplementary Table S1. Previous published DTI studies on 22q11DS. Summary of previous published DTI studies comparing probands with 22q11DS and healthy controls.

Author (Year)	MRI Magnet Strength	N (22q11DS / Healthy Controls)	22q11DS Age [mean (SD)]	Healthy Age [mean (SD)]	FA effects	MD effects	AD effects	RD effects	Brief Study Findings
Barnea-Goraly et al. 2003	1.5T	38 (19/19)	12.2 (3.9)	14.4 (4.2)	Mixed	NA^	NA	NA	Lower FA in Frontal, Temporal and Parietal lobes. Higher FA in Occipital Lobes (Splenium)
Simon et al. 2005	1.5T	36 (18/18)	9.8 (1.4)	10.4 (1.9)	Mixed	NA	NA	NA	Lower FA in Corpus Callosum; Higher FA in Cingulate gyrus & Parietal lobe and Corpus Callosum
Simon et al.* 2008	1.5T	36 (18/18)	9.8 (1.4)	10.4 (1.9)	22q>HC	HC>22q	HC>22q	HC>22q	Higuer FA and lower RD in Fronto-occipital fasciculus nd Superior Longitudinal Fasciculus; Lower RD in Parietal lobe, Tapetum, Optic Radiation, Forceps Major; *Reanalysis of Simon et al. 2005
Sundram et al. 2008	1.5T	23 (11/12)	12 (2.2)	13 (2.5)	Mixed	NA	NA	NA	Lower FA in PLIC, superior Corona Radiata, Tapetum, PTR, Arcuate fasciculus; Higher FA in Genu of Corpus Callosum, ALIC, anterior Corona Radiata. Only lower FA with IQ included as covariate. Negative correlation between psychotic symptoms and FA.
da Silva Alves et al. 2011	3T	70 (27/31)	29.8 (7.6)	32.4 (9.7)	HC>22q*	NA	NA	NA	Lower FA throughout cortex (frontal, parietal, parahippocampal) in full sample and SZ+ subsample; Lower FA in parietal cortex in SZ- sample; *Mixed FA results w/o IQ as covariate with increased FA in Cyngulate gyrus. Negative correlation between pshychotic symptoms and FA
Kikinis et al. 2012	1.5T	18 (9/9)	27.3 (7.1)	27.2 (6.9)	HC>22q	NA	HC>22q	No significant results	Lower FA and lower AD in Parietal lobe: intersection of IFO, SFL, ILF, Cingulum and ATR.
Radoeva et al. 2012	1.5T	49 (33/16)	17.7 (1.8)	18 (1.7)	HC>22q	NA	HC>22q	HC>22q	Lower FA in Uncinate Fasciculus. Higher AD in SCP, PTR, ACR, SCR, PCR, CGC, SLF, IFO, SS, EC, RLIC. RD changes in Corona Radiata. ^Unaffected siblings only in comparison sample.
Villalon-Reina et al. 2013	3T	39 (19/20)	10.7 (1.8)	10.1 (2.2)	HC>22q	Mixed	Mixed	Mixed	Lower FA in Superior Temporal gyri & Superior Corona Radiata. *Female only sample; Other genetic disorders included

Author (Year)	MRI Magnet Strength	N (22q11DS / Healthy Controls)	22q11DS Age [mean (SD)]	Healthy Age [mean (SD)]	FA effects	MD effects	AD effects	RD effects	Brief Study Findings
Perlstein et al.[a] 2014	1.5T	99 (52/47)	18 (2.2)	18.1 (1.6)	Mixed	NA	HC>22q	HC>22q	Lower FA, AD in Fornix; Higher FA, lower RD in ALIC. Lower AD, RD in Uncinate. [a]Follow-up study to Radioeva, 2012 - Healthy group= HC+unaffected siblings
Jalbrzikowski et al. 2014	3T	65* (36/29)	16.3 (4.3)	15.5 (3.8)	Mixed	NA	HC>22q	HC>22q	Higher FA, lower AD and RD throughout white matter (whole-brain analysis). Lower FA in Cingulate Gyrus proximal to Hippocampus (ROI-analysis). *Multi-scanner study
Deng et al. 2015	3T	81* (43/38)	10.83 (1.9)	10 (2.3)	HC>22q	22q>HC	No significant results	HC>22q	Lower FA in Fornix; Fornix was only ROI reported. *Tractography study of the Fornix - some 22q data failed tract estimation
Kates et al.° 2015	1.5T	97 (51/46)	18 (2.3)	18 (1.6)	Mixed	NA	HC>22q	HC>22q	Lower FA in anterior Cingulum (CB), higher FA in posterior CB; Lower RD and AD in CB. Lower FA in CB in 22q not treated with antipsychotic or mood stabilizers; °Follow-up study to Radioeva 2012 and Perlstein, 2014 - healthy group=HC+unaffected siblings; *Tractography study of the Cingulum.
Kikinis et al.^ 2016	1.5T	97 (50/47)	18.1 (2.3)	18 (1.6)	No significant results	HC>22q	HC>22q	HC>22q	Lower MD, AD, RD in Corpus Callosum, SLF, Corona Radiata. Lower AD in high risk for psychosis 22q patients. ^Follow-up study to Radioeva 2012 and Perlstein, 2014 and Kates, 2015 - healthy group=HC+unaffected siblings;
Bakker et al.* 2016	3T	54 (21/33)	26.2 (3.6)	26.2 (5.5)	22q>HC	HC>22q	HC>22q	HC>22q	Higher FA in Corpus Callosum, FOF, and Thalamic Radiations. Lower AD, RD, MD in Corpus Callosum, Thalamic Radiations, SLF, ILF, FOF. *Study also compared 22qDS to non-22q psychosis ultra-high-risk subjects.
Olszewski et al. 2017	3T	87 (57/30*)	20.8 (2.29)	20.9 (1.46)	22q>HC	NA	No significant results	HC>22q	Higher FA and lower RD in IFO, Cingulum, ATR. Lower RD in ILF. Prodromal symptoms and psychosis related to higher FA and lower RD in IFO. *12 subjects were unaffected siblings.
Roalf 2017 et al. 2017	3T	78 (39/39)	19.8 (4.25)	19.9 (1.73)	HC>22q	HC>22q	HC>22q	22q>HC	Lower FA in CGC and CGH. Lower MD in ILF. Lower AD in Forceps major, CGC, IFO, ILF, SLF. Higuer RD in CGH.

Supplementary Table S2a. Demographics of 22q11DS probands. Demographics of 22q11DS probands: deletion size and psychosis diagnosis. AD = typical deletion type A-D; AB = deletion type A-B; Other = Other atypical deletions.

Site	Deletion Type	Psychotic Disorders
**UPenn**	36 AD; 4 AB; 2 Other	4 Yes; 39 No
**UCLA**	40 AD; 4 AB; 4 Other	5 Yes; 44 No
**SUNY Upstate**	17 AD; 1 AB	2 Yes; 32 No
**University of Newcastle**	9 AD	—
**Maastricht University**	13 AD; 4 AB	10 Yes; 14 No
**Institute of Psychiatry London**	10 AD; 1 AB	3 Yes; 19 No
**UC Davis #2**	40 AD; 1 Other	—
**UC Davis #1**	10 AD	—
**Cardiff University**	5 AD	—
**Utrecht University**	26 AD; 1 AB; 1 Other	11 Yes; 43 No
**Total**	206 (89.9%) AD;<br>15 (6.5%) AB;<br>8 (3.5%) Other	35 (15.5%) Yes;<br>191 (84.5%) No

Table S2b. Demographic information of the 22q11DS participants with and without psychosis. Age distribution for both groups is non-normal (Shapiro-Wilk test). Test statistic and p-values are shown in the table. A percentile bootstrap test was used to estimate the group differences of age. 95% confidence intervals (CI) are shown (reject H_0 if CI contains zero). SD = Standard deviation.

	22q11.2 DS with Psychosis	22q11.2 DS without Psychosis	Group differences
**Number of participants**	35 (15.5%)	191 (84.5%)	Total participants: 226
**Age (mean, SD)**	mean: 23.87; SD: 8.02<br>W = 0.92<br>p = 0.026	mean:17.99; SD: 5.78<br>W = 0.94<br>p = 1.004e-06	Non-equal means:<br>95% CI =<br>(-8.711487 -3.069191)<br>Equal variances:<br>95% CI =<br>(-82.57228 22.51956)
**Sex**	15 (43%) F;<br>20 (57%) M	81 (42%) F;<br>110 (58%) M	X^2 = 5.11 (p = 0.023)

Supplementary Table S2c. Psychotropic Medications of the 22q11DS Participants across Sites at the time of data acquisition.

Site	Typical Antipsychotic	Atypical Antipsychotic	Anti-convulsant	Psycho-stimulant	Anti-depressant	Lithium
**UCLA**	3	2	3	10	19	0
**Davis_1**	0	1	1	6	1	1
**Davis_2**	0	2	1	11	3	0
**IoP**	0	4	0	0	4	0
**Maastricht**	0	10	1	2	5	2
**Newcastle**	0	2	1	1	2	0
**SUNY**	0	5	1	3	8	0
**UPenn**	2	6	2	6	15	0
**Utrecht**	1	5	2	5	2	1
**Total**	**6**	**37**	**12**	**44**	**59**	**4**

Supplementary Table S3. Clinical characteristics of study participants. Clinical characteristics of 22q11DS and control participants, across sites: inclusion/exclusion criteria and instruments for diagnosing psychotic disorder and psychotic symptom severity, by site. References indicate representative publications for each study sample.

Site	Study Inclusion and Exclusion criteria	Instrument for psychiatric diagnosis / Rating psychotic symptoms severity	Instrument for IQ Assessment	Citations
UCLA (1,2)	Inclusion Criteria 22q11DS and Controls:<br>1) no significant abuse of drugs or alcohol during the last 6 months or prior abuse/ dependence likely to lead to central nervous system impairment;<br>2) between 5 and 50 years of age;<br>3) sufficient acculturation and fluency in the English language to avoid invalidating research measures<br><br>Inclusion Criteria 22q11DS Only:<br>1) Confirmed diagnosis of 22q11.2 microdeletion (by FISH or microarray)<br>2) no evidence of a comorbid neurological disorder (e.g., uncontrolled epilepsy, encephalitis);<br>3) Verbal IQ >= 60 in order to complete the Structured Interview for Prodromal Syndromes<br><br>Inclusion Criteria Controls Only:<br>1) no evidence of current or past significant psychopathology;<br>2) self- and parent report of no prior treatment for psychiatric disorder;<br>3) no evidence of traumatic brain injury, or other neurological disorder or impairment;<br>4) no history of significant medical complications likely to affect cognitive functioning (e.g., Type I diabetes, cancer, neural tube defects, etc.)<br>5) no first-degree relative has been diagnosed with or treated for psychotic disorder; and<br>8) sex, age, race, and parental educational level comparable to that of the patient participants<br>6) verbal IQ >= 70	SCID[1] interview (over 10)<br>C-DISC[2] (18 & under)<br>SIPS[3]<br>BPRS[4]	WASI [57]	Ho et al., 2012[14]<br>Jalbrzikowski et al., 2012[15], 2013[16], 2014[17]<br>Jonas et al., 2015[18]<br>Schreiner et al., 2013[19], 2017[20]

Site	Study Inclusion and Exclusion criteria	Instrument for psychiatric diagnosis / Rating psychotic symptoms severity	Instrument for IQ Assessment	Citations
State University of New York at Upstate (SUNY)	Inclusion Criteria 22q11DS and Controls:<br>1) Between the ages of 9 and 15 years of age at the 1st timepoint, or 12 and 18 years if they entered the study at the 2nd timepoint.<br>2) No orthodontia or paramagnetic implants.<br>3) Birthweight Over 2500 grams<br>4) No traumatic brain injury or loss of consciousness for > 15 minutes<br>5) No fetal exposure to drugs or alcohol<br><br>Inclusion Criteria 22q11DS Only:<br>Confirmed diagnosis of 22q11.2 microdeletion (by FISH or microarray)<br><br>Exclusion Criteria Controls Only:<br>1) History of severe psychiatric disorder in self or 1st degree relatives<br>2) Placement in a gifted or special education classroom<br>3) Seizure or other neurological / genetic disorder	SCID[1]<br>SIPS[3]<br>BPRS[4]<br>K-SADS-PL[5]	WISC-III [58]	Radoeva et al., 2014[21], Antshel et al., 2013[22], Radoeva et al., 2012[23], Kunwar et al.,2012[24], Kates et al., 2011[25], Kates et al., 2011[26], Coman et al., 2010[27], Roizen et al., 2010[28], Antshel et al., 2010[29], Antshel et al., 2008[30], Kates et al., 2007[31], Antshel et al., 2007[32], Kates et al., 2007[33], Antshel et al., 2007[34], Aneja et al., 2007[35], Antshel et al., 2006[36], Kates et al., 2006[37]
UC Davis	Inclusion Criteria 22q11DS Only:<br>Confirmed diagnosis of 22q11.2 microdeletion (by FISH or microarray)<br><br>Exclusion Criteria 22q11DS cases and Controls:<br>1) Brain infarct, CNS infection, head injury, other focal neurologic abnormality<br>2) Current or past use of antipsychotic medications<br>3) Existing diagnosis of psychosis	SCID[1]<br>SIPS[3]	WISC-IV [59]<br>WASI [57]	Scott et al., 2016[38],<br>Deng et al., 2015[39],<br>Stephenson et al., 2014[40]
University of Pennsylvania/ Children's Hospital of Philadelphia	Inclusion Criteria 22q11DS and Controls:<br>1) Age ≥8<br>2) Ability to provide informed consent/ assent<br>3) English proficiency<br>4) Ambulatory and stable medical status<br>5) Estimated IQ >70<br><br>Inclusion Criteria 22q11DS Only:<br>Confirmed diagnosis of 22q11.2 microdeletion (by FISH or microarray)<br><br>Exclusion Criteria 22q11DS and Controls:<br>1) Pervasive developmental disorder per medical records or mental retardation (IQ<70)<br>2) Medical or neurological disorders that may affect brain function (e.g., uncontrolled seizures, head trauma, CNS tumor, and infection) or visual performance (e.g., blindness).	K-SADS-PL[5]<br>SIPS[3]	WISC [59]<br>WAIS [60]	Yi et al., 2013 [41],<br>Niarchou et al., 2017[42],<br>Tang et al., 2017[43],<br>Tang et al., 2017[44]

Site	Study Inclusion and Exclusion criteria	Instrument for psychiatric diagnosis / Rating psychotic symptoms severity	Instrument for IQ Assessment	Citations
Utrecht University Medical Center, Netherlands	Inclusion Criteria 22q11DS:<br>1) Confirmed diagnosis of 22q11.2 deletion<br>2) age >=12<br>3) VIQ >55	K-SADS-PL[5]	Dutch version of:<br>WISC-III [58]<br>or<br>WISC-R [61];<br>WAIS –III [60]	Fiksinski et al., 2017[45]
Kings College (Institute of Psychiatry), London	Inclusion Criteria 22q11DS and Controls:<br>1) no significant abuse of drugs or alcohol during the last 6 months or prior abuse/ dependence likely to lead to central nervous system impairment;<br>2) between 5 and 50 years of age;<br>3) sufficient acculturation and fluency in the English language to avoid invalidating research measures<br><br>Inclusion Criteria 22q11DS Only:<br>1) Confirmed diagnosis of 22q11.2 microdeletion by FISH test<br>2) no evidence of a comorbid neurological disorder (e.g., uncontrolled epilepsy, encephalitis);<br>3) Verbal IQ >= 60 in order to complete the Structured Interview for Prodromal Syndromes<br><br>Inclusion Criteria Controls Only:<br>1) no evidence of current or past significant psychopathology or psychiatric disorders;<br>2) no evidence of traumatic brain injury, or other neurological disorder or impairment;<br>3) no history of significant medical complications likely to affect cognitive functioning (e.g., Type I diabetes, cancer, neural tube defects, etc.)<br>4) no first-degree relative has been diagnosed with or treated for psychotic disorder; and<br>5) sex, age, race, and parental educational level comparable to that of the patient participants<br>6) verbal IQ >= 70	SCID[1]<br>C-DISC[2]<br>SIPS[3]<br>BPRS[4]	WASI [57]	In preparation
Maastricht	Inclusion Criteria 22q11DS and Controls:<br>1) age >18 years<br><br>Inclusion Criteria 22q11DS Only:<br>Confirmed diagnosis of 22q11.2 microdeletion (by FISH or microarray)<br><br>Exclusion Criteria 22q11DS and Controls:<br>Present substance use or history of abuse or dependency, neurological affliction, or pregnancy.	PANSS[6]<br>MINI[7]<br>PAS-ADD[8]	Dutch version of<br>WAIS –III [60]	Bakker et al., 2016[46]<br>Da Silva Alves et al., 2011[47]

Site	Study Inclusion and Exclusion criteria	Instrument for psychiatric diagnosis / Rating psychotic symptoms severity	Instrument for IQ Assessment	Citations
Cardiff	Inclusion Criteria 22q11DS and Controls:<br>1) Age ≥ 10<br><br>Inclusion Criteria 22q11DS Only:<br>Confirmed diagnosis of 22q11.2 microdeletion (by FISH or microarray)<br><br>Exclusion Criteria 22q11.2DS and Controls: Contraindications for MRI scanning (e.g. MRI incompatible implants or prostheses)	CAPA [9]<br>SIPS[3]<br><br>Adults only: SIPS[3]<br>PANSS6<br>PAS-ADD[8]<br>SCID-II [10]<br>SAPS[11]<br>SANS[12]<br>SPI-A[13]	WASI [57]	Monks et al., 2014[48],<br>Niarchou et al., 2014[49], 2015[50],<br>Chawner et al., In press[51]
Newcastle	Inclusion Criteria 22q11DS and Control:<br>1) English language fluency<br><br>Inclusion Criteria 22q11DS Only:<br>Confirmed diagnosis of 22q11.2 microdeletion (by FISH or microarray)<br><br>Exclusion Criteria 22q11DS and Controls: Clinically detectable medical disorder known to affect brain structure (e.g., hypertension), or a history of head injury.<br><br>Exclusion Criteria Controls Only:<br>1) The presence of a genetic disorder, mental health problems, a history of severe head injury, seizure disorder, or other ocular, neurological or major medical problems that could influence task performance.	K-SADS-PL[5]<br>SCID[1]	WISC-III [58]<br>WASI [57]	Campbell et al., 2010[52], 2015[53],<br>McCabe et al., 2012[54],<br>2013[55],2014[56]

Supplementary Table S4. DMRI acquisition parameters. DMRI sequence acquisition parameters by site. Although some sites (UCLA, Newcastle, Cardiff, Utrecht) used two scanners to acquire data, acquisition parameters were identical across scanners for each site.

Site	In-plane Resolution (acquisition)	In-plane Resolution (upsampled)	FOV (mm)	Slice Thickness (mm)	In-Plane Voxel Size (acquisition) (mm)	In-Plane Voxel Size (upsampled) (mm)	Number of Slices	No. of Diffusion Directions	b-value (s/mm^2)	Field Strength	Scanner Type	TR (ms)	TE (ms)
**Cardiff**	96 x 96	256 x 256	230	2.4	2.39 x 2.39	1.79 x 1.79	60	30	1000	3T	GE Signa	18750	92.6
**Maastricht**	112 x 112	256 x 256	230	3	2.05 x 2.05	0.89 x 0.89	38	32	1000	3T	Phillips Intera	4834	94
**SUNY**	128 x 128	--	256	2	2 x 2	--	73	64	900	3T	Siemens Tim Trio	10000	87
**UCLA**	96 x 96	--	190	2	1.97 x 1.97	--	50	64	1000	3T	Siemens Tim Trio	7100	93
**UPenn**	128 x 128	--	240	2	1.87 x 1.87	--	70	64	1000	3T	Siemens Tim Trio	8100	82
**Davis_1**	128 x 128	--	220	3	1.71 x 1.71	--	40	12	1000	3T	Siemens Tim Trio	6700	99
**Davis_2**	128 x 128	256 x 256	230	1.8	1.79 x 1.79	0.89 x 0.89	64	60	700	3T	Siemens Tim Trio	6600	70
**IoP**	96 x 96	256 x 256	210	2	2.18 x 2.18	0.82 x 0.82	52	64	1000	3T	GE Signa	8000	95.6
**Newcastle**	104 x 104	--	250	2.4	2.40 x 2.40	--	65	64	1000	1.5T	Siemens Avanto	8400	88
**Utrecht**	128 x 99	128 x 128	240	2	1.87 x 2.4	1.87 x 1.87	75	30	1000	3T	Phillips Ingenia	7011	68

Supplementary Table S5. T1-weighted acquisition parameters. Acquisition parameters of the T1-weighted scans, by site.

Site	Scanner vendor and type	Sequence	Field Strength	Acquisition Direction	# of Slices	Slice Thickness (mm)	Voxel Size (mm3)	TI (ms)	TE (ms)	TR (ms)	Flip Angle
**UCLA 1**	Siemens Tim Trio	MPRAGE	3T	sagittal	160	1.2	1.0x1.0x1.2	900	2.86	2300	9
**UCLA 2**	Siemens Tim Trio	MPRAGE	3T	sagittal	160	1.2	1.0x1.0x1.2	900	2.91	2300	9
**SUNY**	Siemens Tim Trio	MPRAGE_SAG_BWM	3T	sagittal	176	1	1.0x1.0x1.0	1100	3.31	2530	7
**UC Davis 1**	Siemens Tim Trio	MPRAGE_SAGMagdeburgIPAT	3T	axial	160	1	1.0x1.0x1.0	1100	2.93	1820	12
**UC Davis 2**	Siemens Tim Trio	MPRAGE_0.9mm iso w/flow comp	3T	sagittal	192	0.9	0.9x0.9x0.9	1100	4.37	2200	7
**University of Penn**	Siemens	MPRAGE	3T	axial	160	1	0.937x 0.937x1.0	1100	3.51	1810	9
**Utrecht**	Philips Achieva, since March 2016 Philips Ingenia	3D T1TFE	3T	axial	160	1	0.875x 0.875x1.0	821.92	4.6	9960	8
**IoP**	GE	MPRAGE	3T	sagittal	166	1.2	1.0x1.0x1.0	650	2.9	6900	8
**Maastricht**	Philips Intera	MPRAGE	3T	axial	120	1.2	1.17×1.17×1.20	807.89	4.6	9800	8
**Cardiff**	GE	3D FSPGR	3T	oblique-axial	172	1	1.0x1.0x1.0	450	3	7900	20
**Cardiff**	Siemens Prisma	MPRAGE	3T	sagittal	176	1	1.0x1.0x1.0	850	3.06	2300	9
**Newcastle**	Siemens Avanto	MPRAGE	1.5T	sagittal	176	1	0.98x0.98x1.0	1100	4.3	1980	15

Supplementary Table S6. Meta-analysis results: 22q11DS vs. Controls. Cohen's d effect sizes for Diagnosis and Sex and partial correlation r-values of Age and [Age-mean(Age)]2 for each ROI, by DTI measure. The model was tested on each 22q11DS case-control dataset (excluding Utrecht), and effect sizes and correlations were further meta-analyzed. The model tested was:

$DTI\text{-}ROI\text{-}measure=\beta_0+\beta_1 Diagnosis+\beta_2 Sex+\beta_3 Age+\beta_4 Age^2_{centered}$.

Blue-shadowed cells indicate a statistically significant result that passed the False Discovery Rate threshold at a q-value of 0.05.

JHU-ROI	FA								
	Diagnosis			Sex		Age		[Age-mean(Age)]²	
	d	p	st.error	d	p	r	p	r	p
ACR	0.228	0.133	0.151	0.002	0.986	0.179	0.004	0.181	0.004
ALIC	0.642	1.04E-12	0.090	0.030	0.816	0.332	1.06E-07	0.177	0.019
Average WM	0.094	0.287	0.088	0.429	0.001	0.331	0.001	0.243	3.58E-04
BCC	0.372	2.69E-05	0.089	0.181	0.159	0.230	0.003	0.247	0.001
CGC	0.199	0.036	0.095	0.410	0.002	0.310	0.001	0.230	1.68E-04
EC	-0.466	1.10E-04	0.121	-0.331	0.010	0.305	1.92E-06	0.176	0.005
FXST	-0.300	0.006	0.110	0.598	5.42E-06	0.180	0.004	0.123	0.055
GCC	0.582	4.19E-09	0.099	0.182	0.158	0.139	0.029	0.167	0.008
UNC	0.034	0.698	0.088	-0.189	0.142	0.206	0.001	0.168	0.007
PCR	0.520	5.83E-09	0.089	0.507	1.04E-04	0.317	1.78E-05	0.162	0.010
PLIC	0.809	1.93E-15	0.102	0.379	0.003	0.150	0.017	0.185	0.034
PTR	-0.008	0.943	0.116	0.085	0.507	0.237	0.001	0.152	0.016
RLIC	0.195	0.065	0.105	0.326	0.012	0.298	1.16E-06	0.112	0.081
SCC	0.440	2.18E-04	0.119	-0.074	0.566	0.181	0.004	0.120	0.060
SCR	0.263	0.003	0.088	0.513	8.49E-05	0.203	0.004	0.115	0.074
SFO	-0.117	0.513	0.180	0.133	0.301	0.221	0.001	0.184	0.003
SLF	-0.324	2.42E-04	0.088	0.113	0.379	0.316	0.002	0.178	0.005
SS	0.084	0.488	0.121	0.300	0.020	0.328	2.28E-07	0.140	0.027
TAP	0.864	6.00E-21	0.092	0.943	3.97E-12	0.262	6.03E-05	0.175	0.005

JHU-ROI	MD								
	Diagnosis			Sex		Age		[Age-mean(Age)]²	
	d	p	st.error	d	p	r	p	r	p
ACR	-1.025	7.80E-28	0.094	0.517	7.68E-05	0.307	4.44E-05	0.208	0.001
ALIC	-0.337	0.007	0.126	0.038	0.768	0.312	9.83E-08	0.159	0.012
Average WM	-0.166	0.268	0.150	-0.494	1.52E-04	0.147	0.037	0.143	0.024
BCC	-0.765	6.66E-12	0.111	-0.477	2.531E-04	0.234	0.004	0.272	0.004
CGC	-0.663	3.45E-08	0.120	-0.492	1.642E-04	0.339	1.73E-05	0.177	0.005
EC	-0.383	1.32E-04	0.100	-0.109	0.394	0.329	5.83E-06	0.183	0.004
FXST	-0.447	0.002	0.144	-0.893	3.83E-11	0.278	2.72E-06	0.084	0.197
GCC	-1.039	2.69E-15	0.131	-0.125	0.329	0.205	0.001	0.140	0.028
UNC	-0.357	0.006	0.130	-0.204	0.114	0.236	0.001	0.243	0.003
PCR	-1.341	8.56E-26	0.128	-0.317	0.014	0.344	5.75E-06	0.218	3.71E-04
PLIC	-0.173	0.415	0.212	-0.455	4.676E-04	0.263	1.05E-05	0.200	0.027
PTR	-1.137	9.85E-19	0.129	-0.877	7.994E-11	0.232	1.55E-04	0.131	0.040
RLIC	-0.771	2.04E-10	0.121	-1.202	1.107E-17	0.263	0.001	0.134	0.036
SCC	-0.884	1.16E-13	0.119	-0.709	8.976E-08	0.280	2.61E-06	0.180	0.004
SCR	-0.724	2.11E-05	0.170	-0.319	0.014	0.309	1.47E-04	0.175	0.005
SFO	-0.462	2.23E-07	0.089	-0.279	0.031	0.251	3.65E-05	0.171	0.007
SLF	-0.907	3.67E-10	0.145	-0.723	5.22E-08	0.338	6.43E-05	0.212	0.001
SS	-0.912	6.65E-10	0.148	-1.328	1.44E-20	0.251	3.05E-05	0.123	0.054
TAP	-0.873	7.56E-09	0.151	-0.365	0.005	0.335	9.64E-06	0.151	0.017

JHU-ROI	AD								
	Diagnosis			Sex		Age		[Age-mean(Age)]²	
	d	p	st.error	d	p	r	p	r	p
ACR	-0.870	3.81E-21	0.092	0.494	1.52E-04	0.252	2.80E-05	0.079	0.220
ALIC	0.170	0.172	0.125	-0.030	0.812	0.124	0.052	0.107	0.096
Average WM	-0.293	0.148	0.202	-0.437	0.001	0.152	0.016	0.122	0.056

**BCC**	-0.512	1.06E-08	0.089	-0.899	2.99E-11	0.165	0.009	0.210	0.046
**CGC**	-0.544	1.25E-08	0.096	-0.174	0.177	0.156	0.013	0.136	0.034
**EC**	-0.926	1.20E-23	0.092	-0.586	8.07E-06	0.167	0.008	0.095	0.137
**FXST**	-0.677	2.88E-09	0.114	-0.248	0.054	0.146	0.021	0.097	0.134
**GCC**	-0.747	6.49E-16	0.092	0.226	0.079	0.241	7.08E-05	0.092	0.155
**UNC**	-0.317	3.42E-04	0.088	-0.431	0.001	0.156	0.014	0.110	0.097
**PCR**	-1.244	9.00E-29	0.112	0.315	0.015	0.226	1.92E-04	0.162	0.011
**PLIC**	0.572	0.001	0.166	-0.195	0.130	0.214	0.001	0.168	0.008
**PTR**	-1.336	3.41E-13	0.184	-0.822	8.95E-10	0.188	0.003	0.100	0.119
**RLIC**	-0.746	2.20E-16	0.091	-0.761	1.13E-08	0.186	0.003	0.118	0.064
**SCC**	-0.619	5.63E-12	0.090	-0.844	3.45E-10	0.228	1.95E-04	0.173	0.006
**SCR**	-0.344	4.89E-04	0.099	0.113	0.381	0.186	0.003	0.129	0.044
**SFO**	-0.503	6.96E-07	0.101	-0.270	0.036	0.193	0.002	0.157	0.013
**SLF**	-1.332	1.84E-15	0.168	-0.577	1.10E-05	0.152	0.016	0.134	0.036
**SS**	-0.983	1.20E-10	0.153	-0.887	4.98E-11	0.127	0.047	0.126	0.048
**TAP**	-0.060	0.557	0.103	0.309	0.017	0.218	4.09E-04	0.086	0.183

**JHU-ROI**	**RD**								
	**Diagnosis**			**Sex**		**Age**		**[Age-mean(Age)]^2**	
	**d**	**p**	**st.error**	**d**	**p**	**r**	**p**	**r**	**p**
**ACR**	-0.765	3.46E-11	0.115	0.238	0.065	0.256	3.51E-04	0.235	1.36E-04
**ALIC**	-0.628	3.19E-12	0.090	0.030	0.816	0.356	8.31E-09	0.174	0.006
**Average WM**	-0.193	0.038	0.093	-0.511	9.17E-05	0.318	2.21E-05	0.238	1.02E-04
**BCC**	-0.594	3.78E-11	0.090	-0.294	0.023	0.264	0.002	0.295	0.001
**CGC**	-0.268	0.002	0.088	-0.435	0.001	0.353	5.98E-05	0.227	2.23E-04
**EC**	0.142	0.211	0.113	0.199	0.122	0.348	8.33E-07	0.181	0.004
**FXST**	-0.094	0.388	0.109	-0.759	1.22E-08	0.268	2.65E-05	0.117	0.068
**GCC**	-0.812	7.06E-19	0.092	-0.282	0.029	0.120	0.059	0.154	0.015
**UNC**	-0.147	0.168	0.106	0.105	0.414	0.227	1.82E-04	0.241	0.004
**PCR**	-0.953	8.50E-25	0.093	-0.519	7.17E-05	0.339	2.30E-05	0.202	0.001

**PLIC**	-0.694	3.03E-07	0.136	-0.483	2.12E-04	0.173	0.006	0.160	0.107
**PTR**	-0.659	8.47E-08	0.123	-0.469	3.23E-04	0.295	2.45E-04	0.182	0.004
**RLIC**	-0.471	3.79E-06	0.102	-0.801	2.19E-09	0.294	2.95E-05	0.107	0.094
**SCC**	-0.691	1.55E-12	0.098	-0.234	0.069	0.228	4.29E-04	0.145	0.022
**SCR**	-0.613	1.02E-11	0.090	-0.536	4.25E-05	0.257	0.002	0.138	0.030
**SFO**	-0.254	0.037	0.122	-0.207	0.108	0.232	4.33E-04	0.155	0.014
**SLF**	-0.278	0.002	0.088	-0.475	2.68E-04	0.339	3.39E-04	0.200	0.001
**SS**	-0.560	2.54E-05	0.133	-0.963	1.54E-12	0.357	4.20E-07	0.145	0.022
**TAP**	-0.965	1.57E-10	0.151	-0.805	1.84E-09	0.313	1.40E-05	0.187	0.003

Supplementary Table S7. Mega-analysis results: 22q11DS vs. Controls. Cohen's d effect sizes, Student's t-values, and corresponding p-values for effects of Diagnosis, Sex, Age, and [Age-mean(Age)]2 for each ROI, by DTI measure. The model was tested on the entire ENIGMA-DTI 22q11DS case-control sample (excluding Utrecht) using the harmonized data. The model tested was: *DTI-ROI-measure*=$\beta_0+\beta_1 Diagnosis+\beta_2 Sex+\beta_3 Age+\beta_4 Age^2_{centered}$.
Blue-shadowed cells highlight the p-values passing the False Discovery Rate threshold at a q-value of 0.05.

**JHU-ROI**	**FA**								
	**Diagnosis**			**Sex**		**Age**		**[Age-mean(Age)]²**	
	**d**	**t**	**p**	**t**	**p**	**t**	**p**	**t**	**p**
**ACR**	0.212	2.450	0.015	0.340	0.731	5.430	8.46E-08	-5.080	5.16E-07
**ALIC**	0.614	7.100	3.93E-12	1.840	0.066	11.830	5.00E-16	-4.430	1.16E-05
**Average WM**	0.070	0.804	4.22E-01	2.538	0.011	12.365	5.00E-16	-7.410	5.07E-13
**BCC**	0.289	3.340	0.001	1.430	0.154	5.420	9.10E-08	-4.500	8.49E-06
**CGC**	0.176	2.040	0.042	2.420	0.016	10.820	5.00E-16	-6.220	9.75E-10
**EC**	-0.487	-5.640	2.80E-08	2.490	0.013	9.230	5.00E-16	-4.700	3.26E-06
**FXST**	-0.311	-3.600	3.51E-04	0.530	0.598	4.310	1.96E-05	-2.860	0.004
**GCC**	0.550	6.360	4.38E-10	2.480	0.013	2.310	0.021	-3.610	3.34E-04
**UNC**	0.041	0.470	0.638	2.490	0.013	3.730	2.10E-04	-2.470	0.014
**PCR**	0.477	5.520	5.32E-08	2.270	0.024	8.560	1.11E-16	-6.470	2.27E-10
**PLIC**	0.797	9.220	5.00E-16	2.750	0.006	4.880	1.42E-06	-3.210	0.001

**PTR**	-0.049	-0.560	0.575	0.760	0.446	5.370	1.19E-07	-5.240	2.26E-07
**RLIC**	0.151	1.750	0.080	1.330	0.184	6.730	4.25E-11	-5.070	5.48E-07
**SCC**	0.434	5.020	7.10E-07	-0.170	0.864	4.340	1.71E-05	-4.210	2.98E-05
**SCR**	0.264	3.050	0.002	1.640	0.101	6.530	1.57E-10	-4.500	8.45E-06
**SFO**	-0.079	-0.910	0.362	1.270	0.203	6.510	1.76E-10	-3.520	4.62E-04
**SLF**	-0.326	-3.770	1.79E-04	1.320	0.189	11.080	5.00E-16	-7.140	2.98E-12
**SS**	0.039	0.450	0.654	0.320	0.752	8.880	5.00E-16	-6.610	9.39E-11
**TAP**	0.837	9.680	5.00E-16	1.280	0.201	8.240	1.33E-15	-5.020	7.02E-07

**JHU-ROI**	**MD**								
	**Diagnosis**			**Sex**		**Age**		**[Age-mean(Age)]²**	
	**d**	**t**	**p**	**t**	**p**	**t**	**p**	**t**	**p**
**ACR**	-0.912	-10.540	5.00E-16	-2.060	0.040	-11.330	5.00E-16	5.550	4.54E-08
**ALIC**	-0.319	-3.690	2.47E-04	-1.690	0.092	-9.320	5.00E-16	2.460	0.014
**Average WM**	-0.641	-7.410	4.91E-13	-0.515	0.606	-0.057	9.55E-01	-0.124	9.01E-01
**BCC**	-0.524	-6.060	2.55E-09	-2.130	0.034	-6.180	1.27E-09	4.050	5.86E-05
**CGC**	-0.571	-6.600	9.70E-11	-2.820	0.005	-11.870	5.00E-16	6.760	3.54E-11
**EC**	-0.356	-4.110	4.53E-05	-1.830	0.068	-11.710	5.00E-16	5.750	1.47E-08
**FXST**	-0.433	-5.010	7.43E-07	-1.180	0.237	-9.310	5.00E-16	3.740	2.00E-04
**GCC**	-0.870	-10.060	5.00E-16	-2.680	0.008	-6.120	1.78E-09	3.160	1.67E-03
**UNC**	-0.294	-3.410	7.10E-04	-0.640	0.525	-6.420	2.98E-10	3.810	1.54E-04
**PCR**	-1.132	-13.090	5.00E-16	-1.360	0.176	-12.610	5.00E-16	7.170	2.47E-12
**PLIC**	-0.236	-2.730	0.01	-1.300	0.194	-9.120	5.00E-16	3.890	1.12E-04
**PTR**	-0.936	-10.830	5.00E-16	-0.170	0.866	-8.730	5.00E-16	5.440	8.24E-08
**RLIC**	-0.697	-8.060	4.88E-15	-1.040	0.297	-9.610	5.00E-16	5.580	3.91E-08
**SCC**	-0.737	-8.520	1.11E-16	-1.940	0.053	-9.040	5.00E-16	4.230	2.75E-05
**SCR**	-0.579	-6.690	5.60E-11	-1.570	0.117	-11.190	5.00E-16	6.550	1.37E-10
**SFO**	-0.354	-4.100	4.83E-05	-0.140	0.889	-6.790	3.05E-11	3.500	0.001
**SLF**	-0.722	-8.350	5.55E-16	-0.930	0.351	-13.100	5.00E-16	7.610	1.22E-13
**SS**	-0.812	-9.390	5.00E-16	0.650	0.515	-8.910	5.00E-16	5.450	7.59E-08

TAP	-0.731	-8.450	2.22E-16	-0.950	0.343	-11.970	5.00E-16	7.380	6.11E-13

JHU-ROI	AD								
	Diagnosis			Sex		Age		[Age-mean(Age)]^2	
	d	t	p	t	p	t	p	t	p
ACR	-0.849	-9.820	5.00E-16	-1.580	0.114	-8.220	1.55E-15	2.320	0.021
ALIC	0.127	1.470	0.142	0.280	0.781	-0.400	0.693	-1.220	0.223
Average WM	-0.543	-6.280	6.94E-10	-0.446	0.655	0.393	0.695	-0.529	0.597
BCC	-0.421	-4.870	1.51E-06	-0.600	0.550	-2.610	0.009	0.800	0.426
CGC	-0.528	-6.100	1.98E-09	0.620	0.535	2.180	0.030	-1.500	0.135
EC	-0.905	-10.470	5.00E-16	0.400	0.690	-3.760	1.87E-04	2.010	0.046
FXST	-0.655	-7.580	1.55E-13	-0.540	0.592	-4.710	3.23E-06	1.090	0.274
GCC	-0.672	-7.770	3.99E-14	-1.330	0.185	-5.970	4.40E-09	0.900	0.371
UNC	-0.258	-2.990	0.003	1.620	0.107	-3.030	0.003	2.030	0.042
PCR	-1.129	-13.060	5.00E-16	0.760	0.450	-7.950	1.13E-14	3.390	0.001
PLIC	0.455	5.260	2.04E-07	1.510	0.131	-7.620	1.19E-13	1.920	0.055
PTR	-0.924	-10.680	5.00E-16	-0.100	0.922	-4.840	1.73E-06	2.170	0.031
RLIC	-0.707	-8.180	2.11E-15	0.510	0.613	-4.340	1.71E-05	1.360	0.175
SCC	-0.531	-6.140	1.60E-09	-1.690	0.092	-5.760	1.46E-08	1.360	0.176
SCR	-0.329	-3.810	1.56E-04	0.080	0.934	-6.860	1.87E-11	3.050	0.002
SFO	-0.480	-5.550	4.53E-08	1.420	0.156	-3.250	0.001	1.340	0.182
SLF	-1.145	-13.240	5.00E-16	0.760	0.447	-5.190	2.93E-07	2.770	0.006
SS	-0.863	-9.980	5.00E-16	1.090	0.278	-1.340	0.182	0.180	0.861
TAP	-0.019	-0.220	0.824	0.090	0.928	-6.350	4.73E-10	4.770	2.36E-06

JHU-ROI	RD								
	Diagnosis			Sex		Age		[Age-mean(Age)]^2	
	d	t	p	t	p	t	p	t	p
ACR	-0.677	-7.820	2.75E-14	-1.600	0.111	-9.790	5.00E-16	5.940	5.26E-09
ALIC	-0.579	-6.700	5.30E-11	-2.380	0.018	-12.710	5.00E-16	4.490	8.83E-06

**Average WM**	-0.144	-1.661	9.73E-02	-1.895	0.059	-12.540	5.00E-16	7.080	4.44E-12
**BCC**	-0.391	-4.520	7.46E-06	-1.730	0.084	-6.060	2.58E-09	4.470	9.59E-06
**CGC**	-0.222	-2.570	0.010	-3.190	0.001	-13.420	5.00E-16	7.310	9.47E-13
**EC**	0.134	1.540	0.123	-2.350	0.019	-12.320	5.00E-16	6.040	2.87E-09
**FXST**	-0.061	-0.710	0.480	-1.390	0.164	-9.090	5.00E-16	4.370	1.48E-05
**GCC**	-0.712	-8.230	1.44E-15	-2.640	0.009	-3.700	2.39E-04	3.560	4.06E-04
**UNC**	-0.156	-1.810	0.072	-2.020	0.044	-5.040	6.32E-07	3.020	0.003
**PCR**	-0.828	-9.570	5.00E-16	-2.200	0.028	-11.930	5.00E-16	7.460	3.46E-13
**PLIC**	-0.581	-6.710	4.86E-11	-2.080	0.038	-6.380	3.75E-10	3.610	3.31E-04
**PTR**	-0.556	-6.430	2.88E-10	-0.700	0.486	-8.990	5.00E-16	6.710	5.00E-11
**RLIC**	-0.407	-4.710	3.13E-06	-1.310	0.192	-9.070	5.00E-16	6.060	2.54E-09
**SCC**	-0.568	-6.570	1.18E-10	-0.800	0.426	-7.930	1.29E-14	5.270	1.99E-07
**SCR**	-0.530	-6.130	1.69E-09	-1.740	0.082	-9.590	5.00E-16	6.070	2.45E-09
**SFO**	-0.221	-2.560	0.011	-1.040	0.299	-7.580	1.58E-13	4.060	5.68E-05
**SLF**	-0.220	-2.550	0.011	-1.550	0.122	-14.060	5.00E-16	8.180	2.00E-15
**SS**	-0.479	-5.540	4.87E-08	-0.250	0.802	-11.330	5.00E-16	7.410	4.98E-13
**TAP**	-0.851	-9.850	5.00E-16	-1.090	0.275	-10.470	5.00E-16	6.180	1.24E-09

Supplementary Table S8. Mega-analysis results: 22q11DS vs. Controls, including Age-by-Diagnosis interaction. Mega-analysis results when including the Age-by-Diagnosis interaction term comparing 22q11DS vs. Controls. Student's t and p-values are provided for the effects of Diagnosis and covariates on each ROI, by DTI measure. The model tested was: $DTI\text{-}ROI\text{-}measure=ß_0+ß_1 Diagnosis+ß_2 Sex+ß_3 Age+ß_4 Age^2_{centered}+ß_5(AgeXDiagnosis)$.
This model was tested on the entire ENIGMA-DTI 22q11DS case-control sample (excluding Utrecht) using the harmonized data. No p-values passed the FDR threshold at a q-value of 0.05 for the variable of interest Age-by-Diagnosis.

JHU-ROI	FA									
	Diagnosis		Sex		Age		[Age-mean(Age)]^2		Age-by-Diagnosis	
	t	p	t	p	t	p	t	p	t	p
ACR	0.760	0.450	4.090	4.88E-05	-4.950	9.80E-07	0.360	0.722	0.800	0.804
ALIC	3.060	0.002	9.400	5.00E-16	-4.390	1.34E-05	1.820	0.069	0.820	0.816
Average WM	0.590	0.557	9.850	5.00E-16	-7.330	8.33E-13	2.520	0.012	0.770	0.773
BCC	1.210	0.226	4.150	3.85E-05	-4.400	1.33E-05	1.430	0.153	0.890	0.888
CGC	1.740	0.083	9.100	5.00E-16	-6.300	6.07E-10	2.360	0.019	0.320	0.316
EC	-3.110	0.002	6.650	7.40E-11	-4.460	9.85E-06	2.530	0.012	0.350	0.353
FXST	-3.040	0.002	2.300	0.022	-2.520	0.012	0.620	0.533	0.080	0.083
GCC	2.910	0.004	2.050	0.040	-3.620	3.23E-04	2.460	0.014	0.690	0.695
UNC	0.030	0.979	2.810	0.005	-2.400	0.017	2.500	0.013	0.860	0.859
PCR	1.350	0.177	6.110	1.88E-09	-6.200	1.16E-09	2.320	0.021	0.350	0.347
PLIC	3.030	0.003	3.360	0.001	-3.030	0.003	2.790	0.005	0.470	0.466
PTR	0.310	0.754	4.570	6.18E-06	-5.260	2.06E-07	0.730	0.466	0.560	0.556
RLIC	-0.290	0.773	4.600	5.29E-06	-4.800	2.08E-06	1.390	0.165	0.280	0.279
SCC	4.830	1.81E-06	5.320	1.50E-07	-4.720	3.04E-06	-0.340	0.735	5.00E-16	0.002
SCR	0.630	0.527	4.710	3.24E-06	-4.310	1.95E-05	1.670	0.095	0.520	0.519
SFO	-0.120	0.907	5.260	2.10E-07	-3.510	4.81E-04	1.260	0.209	0.790	0.786
SLF	-2.260	0.024	8.170	2.22E-15	-6.880	1.64E-11	1.360	0.175	0.420	0.417
SS	-0.140	0.887	6.730	4.48E-11	-6.440	2.73E-10	0.330	0.738	0.730	0.726
TAP	4.030	6.43E-05	6.550	1.40E-10	-4.960	9.26E-07	1.270	0.206	0.870	0.873

JHU-ROI	MD									
	Diagnosis		Sex		Age		[Age-mean(Age)]²		Age-by-Diagnosis	
	t	p	t	p	t	p	t	p	t	p
ACR	-4.130	4.24E-05	-8.800	5.00E-16	5.440	8.31E-08	-2.070	0.039	0.910	0.913
ALIC	-0.830	0.405	-6.860	1.94E-11	2.300	0.022	-1.720	0.085	0.480	0.480
Average WM	-3.500	4.95E-04	-0.410	0.685	-0.020	0.985	-0.480	0.630	0.560	0.562
BCC	-1.250	0.212	-4.040	6.23E-05	3.760	1.89E-04	-2.200	0.028	0.200	0.196
CGC	-3.480	0.001	-9.850	5.00E-16	6.820	2.53E-11	-2.760	0.006	0.360	0.364
EC	-1.350	0.179	-8.960	5.00E-16	5.600	3.45E-08	-1.840	0.066	0.740	0.740
FXST	-0.940	0.350	-6.560	1.30E-10	3.480	0.001	-1.250	0.213	0.240	0.240
GCC	-4.370	1.51E-05	-5.010	7.25E-07	3.170	0.002	-2.660	0.008	0.720	0.717
UNC	-2.260	0.024	-5.630	2.87E-08	3.920	9.83E-05	-0.580	0.561	0.330	0.331
PCR	-5.170	3.32E-07	-9.810	5.00E-16	7.040	6.08E-12	-1.360	0.175	0.930	0.930
PLIC	-3.420	0.001	-8.750	5.00E-16	4.300	2.04E-05	-1.170	0.244	0.010	0.012
PTR	-3.610	3.36E-04	-6.330	5.12E-10	5.210	2.75E-07	-0.210	0.832	0.420	0.421
RLIC	-3.000	0.003	-7.360	7.05E-13	5.440	8.24E-08	-1.050	0.292	0.800	0.799
SCC	-4.820	1.89E-06	-8.030	6.33E-15	4.440	1.11E-05	-1.850	0.064	0.130	0.129
SCR	-1.450	0.147	-7.930	1.32E-14	6.210	1.07E-09	-1.640	0.101	0.180	0.178
SFO	0.340	0.731	-3.980	7.95E-05	3.070	0.002	-0.260	0.796	0.030	0.030
SLF	-2.630	0.009	-9.760	5.00E-16	7.350	7.54E-13	-0.970	0.330	0.430	0.429
SS	-2.290	0.022	-5.970	4.22E-09	5.090	5.07E-07	0.560	0.574	0.110	0.105
TAP	-2.920	0.004	-9.040	5.00E-16	7.170	2.60E-12	-0.970	0.330	0.600	0.604

JHU-ROI	AD									
	Diagnosis		Sex		Age		[Age-mean(Age)]²		Age-by-Diagnosis	
	t	p	t	p	t	p	t	p	t	p
ACR	-3.930	9.51E-05	-6.420	2.99E-10	2.280	0.023	-1.580	0.115	0.990	0.994
ALIC	1.520	0.129	0.320	0.747	-1.380	0.167	0.220	0.825	0.310	0.310
Average WM	-3.000	0.003	-0.020	0.986	-0.430	0.669	-0.420	0.677	0.600	0.602

**BCC**	-0.740	0.459	-1.220	0.223	0.550	0.583	-0.670	0.503	0.190	0.186
**CGC**	-2.570	0.011	1.630	0.105	-1.450	0.148	0.630	0.532	0.900	0.897
**EC**	-4.700	3.38E-06	-3.280	0.001	2.070	0.039	0.430	0.669	0.590	0.588
**FXST**	-2.590	0.010	-3.380	0.001	0.990	0.323	-0.560	0.574	0.620	0.623
**GCC**	-3.330	0.001	-4.810	1.98E-06	0.920	0.358	-1.310	0.190	0.820	0.821
**UNC**	-2.290	0.023	-3.110	0.002	2.210	0.027	1.680	0.094	0.240	0.236
**PCR**	-5.470	6.83E-08	-6.380	3.91E-10	3.380	0.001	0.770	0.443	0.800	0.797
**PLIC**	-1.320	0.187	-8.390	4.44E-16	2.580	0.010	1.730	0.084	0.000	1.75E-04
**PTR**	-2.520	0.012	-2.590	0.010	1.790	0.074	-0.200	0.839	0.050	0.053
**RLIC**	-3.900	1.07E-04	-3.820	1.50E-04	1.460	0.145	0.540	0.588	0.500	0.498
**SCC**	-1.810	0.070	-4.060	5.60E-05	1.210	0.228	-1.730	0.085	0.480	0.478
**SCR**	-0.460	0.649	-4.650	4.28E-06	2.790	0.005	0.020	0.985	0.240	0.242
**SFO**	0.240	0.807	-0.870	0.384	0.840	0.401	1.280	0.201	0.010	0.007
**SLF**	-5.020	7.17E-07	-3.860	1.26E-04	2.670	0.008	0.740	0.459	0.750	0.748
**SS**	-2.190	0.029	0.190	0.846	-0.180	0.856	0.980	0.329	0.050	0.046
**TAP**	0.200	0.838	-4.760	2.45E-06	4.640	4.49E-06	0.070	0.942	0.750	0.749

**JHU-ROI**	**RD**									
	**Diagnosis**		**Sex**		**Age**		**[Age-mean(Age)]^2**		**Age-by-Diagnosis**	
	**t**	**p**	**t**	**p**	**t**	**p**	**t**	**p**	**t**	**p**
**ACR**	-3.040	0.003	-7.590	1.46E-13	5.820	1.04E-08	-1.600	0.110	0.910	0.911
**ALIC**	-2.530	0.012	-9.840	5.00E-16	4.380	1.41E-05	-2.390	0.017	0.870	0.868
**Average WM**	-0.470	0.638	-9.680	5.00E-16	6.930	1.24E-11	-1.900	0.058	0.830	0.831
**BCC**	-1.200	0.229	-4.330	1.81E-05	4.280	2.24E-05	-1.770	0.078	0.500	0.504
**CGC**	-1.560	0.118	-10.860	5.00E-16	7.300	1.07E-12	-3.160	0.002	0.560	0.561
**EC**	1.100	0.272	-9.310	5.00E-16	5.850	8.69E-09	-2.380	0.018	0.600	0.600
**FXST**	0.900	0.368	-6.310	5.71E-10	4.080	5.27E-05	-1.460	0.144	0.200	0.196
**GCC**	-3.590	3.59E-04	-3.090	0.002	3.560	4.11E-04	-2.610	0.009	0.750	0.752
**UNC**	-0.810	0.416	-4.000	7.10E-05	2.990	2.95E-03	-2.010	0.045	0.920	0.922
**PCR**	-3.570	3.84E-04	-9.150	5.00E-16	7.290	1.15E-12	-2.220	0.027	0.770	0.771

**PLIC**	-3.010	0.003	-5.210	2.68E-07	3.610	3.29E-04	-2.050	0.041	0.730	0.727
**PTR**	-3.080	0.002	-7.370	6.45E-13	6.700	5.46E-11	-0.670	0.506	0.580	0.584
**RLIC**	-1.270	0.204	-6.680	6.02E-11	5.840	8.92E-09	-1.340	0.180	0.500	0.499
**SCC**	-4.660	3.94E-06	-7.600	1.34E-13	5.590	3.53E-08	-0.680	0.498	0.030	0.028
**SCR**	-1.570	0.116	-6.900	1.45E-11	5.800	1.14E-08	-1.790	0.074	0.330	0.333
**SFO**	0.320	0.751	-5.020	6.95E-07	3.740	2.06E-04	-1.120	0.264	0.140	0.142
**SLF**	-0.270	0.785	-10.490	5.00E-16	7.900	1.55E-14	-1.590	0.113	0.410	0.414
**SS**	-1.490	0.137	-8.370	5.55E-16	7.150	2.92E-12	-0.290	0.769	0.420	0.425
**TAP**	-3.620	3.28E-04	-7.960	1.03E-14	6.020	3.31E-09	-1.110	0.267	0.720	0.715

Supplementary Table S9. Mega-analysis results: 22q11DS vs. Controls age 30 and under, including Age-by-Diagnosis interaction. Mega-analysis results when including only subjects aged 30 or younger (N=245 22q11DS and 269 Controls), and including the Age-by-Diagnosis interaction term. Student's t and p-values are provided for the effects of Diagnosis and covariates on each ROI, by DTI measure. The model tested was: $DTI\text{-}ROI\text{-}measure=ß_0+ß_1Diagnosis+ß_2Sex+ß_3Age+ß_4Age^2_{centered}+ß_5(AgeXDiagnosis)$.
This model was tested on the case-control sample (excluding Utrecht) using the harmonized data. There was no p-value controlling the FDR at a q-value of 0.05 for the Age-by-Diagnosis term.

**JHU-ROI**	**FA**									
	**Diagnosis**		**Age**		**[Age-mean(Age)]²**		**Sex**		**Age-by-Diagnosis**	
	**t**	**p**	**t**	**p**	**t**	**p**	**t**	**p**	**t**	**p**
**ACR**	1.260	0.208	4.140	4.08E-05	-0.680	0.496	0.430	0.668	0.580	0.583
**ALIC**	2.180	0.030	7.920	1.53E-14	-2.760	0.006	1.680	0.094	0.910	0.910
**Average WM**	0.330	0.739	8.450	3.33E-16	-3.790	1.67E-04	2.300	0.022	0.950	0.945
**BCC**	0.450	0.650	3.320	0.001	-2.650	0.008	1.240	0.216	0.510	0.506
**CGC**	1.190	0.234	7.680	8.39E-14	-2.400	0.017	2.170	0.031	0.600	0.604
**EC**	-2.090	0.037	5.880	7.32E-09	-1.430	0.153	2.190	0.029	0.790	0.794
**FXST**	-2.200	0.028	2.260	0.024	-1.120	0.263	0.330	0.741	0.320	0.324
**GCC**	1.810	0.072	1.320	0.187	-1.090	0.277	2.220	0.027	0.750	0.752
**UNC**	1.090	0.276	3.110	0.002	-0.030	0.978	2.280	0.023	0.290	0.287
**PCR**	1.420	0.157	5.830	1.01E-08	-3.500	4.99E-04	2.380	0.018	0.790	0.792

**PLIC**	2.650	0.008	3.300	0.001	-0.470	0.638	2.930	0.004	0.810	0.810
**PTR**	0.760	0.448	4.640	4.52E-06	-2.680	0.008	0.580	0.562	0.290	0.291
**RLIC**	-0.260	0.793	4.350	1.66E-05	-2.350	0.019	1.460	0.145	0.430	0.431
**SCC**	4.190	3.33E-05	5.010	7.43E-07	-2.100	0.036	-0.080	0.936	0.010	0.010
**SCR**	1.000	0.317	4.590	5.57E-06	-3.040	0.002	1.850	0.065	0.910	0.908
**SFO**	0.970	0.333	5.630	3.06E-08	-1.400	0.161	1.370	0.170	0.150	0.154
**SLF**	-1.340	0.180	7.450	4.06E-13	-3.660	2.81E-04	1.360	0.175	0.950	0.949
**SS**	0.230	0.821	6.360	4.57E-10	-3.510	4.89E-04	0.390	0.700	0.870	0.866
**TAP**	3.120	0.002	5.490	6.23E-08	-2.210	0.027	1.050	0.293	0.980	0.983

**JHU-ROI**	**MD**									
	**Diagnosis**		**Age**		**[Age-mean(Age)]²**		**Sex**		**Age-by-Diagnosis**	
	**t**	**p**	**t**	**p**	**t**	**p**	**t**	**p**	**t**	**p**
**ACR**	-3.100	0.002	-7.310	1.03E-12	1.640	0.101	-2.070	0.039	0.770	0.770
**ALIC**	-0.250	0.800	-5.500	6.02E-08	1.840	0.067	-1.820	0.069	0.320	0.319
**Average WM**	-1.730	0.085	0.660	0.507	-1.560	0.119	-0.230	0.814	0.380	0.380
**BCC**	-0.530	0.596	-3.240	0.001	2.860	0.004	-2.310	0.022	0.140	0.145
**CGC**	-2.500	0.013	-8.310	8.88E-16	3.600	3.48E-04	-2.900	0.004	0.720	0.722
**EC**	-0.420	0.676	-7.290	1.16E-12	2.940	0.003	-1.910	0.056	0.320	0.320
**FXST**	-1.280	0.200	-6.210	1.09E-09	1.560	0.119	-1.230	0.219	0.840	0.840
**GCC**	-3.150	0.002	-4.000	7.38E-05	0.140	0.890	-2.540	0.011	0.880	0.881
**UNC**	-1.590	0.112	-4.600	5.46E-06	3.590	3.61E-04	-0.250	0.806	0.620	0.622
**PCR**	-3.750	0.000	-8.230	1.67E-15	3.120	0.002	-1.470	0.141	0.650	0.652
**PLIC**	-3.180	0.002	-7.950	1.20E-14	1.090	0.278	-1.200	0.232	0.020	0.018
**PTR**	-3.380	0.001	-6.060	2.62E-09	2.840	0.005	-0.430	0.667	0.990	0.989
**RLIC**	-2.190	0.029	-6.410	3.31E-10	2.530	0.012	-1.170	0.241	0.690	0.688
**SCC**	-4.150	3.8400E-05	-6.990	8.49E-12	0.790	0.428	-2.010	0.045	0.170	0.168
**SCR**	-0.970	0.331	-6.780	3.34E-11	3.040	0.002	-1.930	0.054	0.240	0.241
**SFO**	-0.310	0.758	-3.990	7.43E-05	2.270	0.024	-0.660	0.513	0.370	0.371
**SLF**	-2.030	0.043	-8.390	4.44E-16	3.710	2.27E-04	-1.150	0.249	0.540	0.535

**SS**	-1.760	0.079	-5.360	1.24E-07	2.560	0.011	0.400	0.690	0.220	0.221
**TAP**	-2.800	0.005	-8.300	9.99E-16	2.320	0.021	-0.980	0.329	0.830	0.831

**JHU-ROI**	**AD**									
	**Diagnosis**		**Age**		**[Age-mean(Age)]^2**		**Sex**		**Age-by-Diagnosis**	
	**t**	**p**	**t**	**p**	**t**	**p**	**t**	**p**	**t**	**p**
**ACR**	-2.210	0.027	-4.640	4.52E-06	1.200	0.232	-1.570	0.117	0.290	0.291
**ALIC**	1.550	0.123	0.750	0.451	-0.550	0.583	0.060	0.951	0.240	0.245
**Average WM**	-1.550	0.121	0.870	0.384	-1.950	0.051	-0.160	0.870	0.500	0.497
**BCC**	-0.870	0.383	-1.290	0.197	1.370	0.172	-1.070	0.285	0.510	0.514
**CGC**	-1.920	0.055	1.540	0.124	0.620	0.537	0.340	0.737	0.970	0.969
**EC**	-2.610	0.009	-2.160	0.031	2.010	0.045	0.080	0.937	0.350	0.346
**FXST**	-2.490	0.013	-3.240	0.001	0.620	0.534	-0.720	0.471	0.900	0.897
**GCC**	-2.450	0.015	-3.950	8.77E-05	-0.460	0.642	-1.360	0.176	0.930	0.927
**UNC**	-0.810	0.419	-1.890	0.060	2.890	0.004	1.830	0.068	0.790	0.787
**PCR**	-3.260	0.001	-4.460	1.00E-05	0.370	0.709	0.710	0.479	0.280	0.281
**PLIC**	-1.340	0.182	-7.280	1.25E-12	0.390	0.698	1.790	0.074	0.000	0.002
**PTR**	-2.320	0.021	-2.490	0.013	0.960	0.339	-0.640	0.524	0.260	0.263
**RLIC**	-2.960	0.003	-3.090	0.002	0.830	0.405	0.420	0.674	0.770	0.775
**SCC**	-1.540	0.124	-3.270	0.001	-1.320	0.189	-1.760	0.079	0.640	0.637
**SCR**	0.470	0.637	-3.320	0.001	0.830	0.408	-0.140	0.892	0.070	0.070
**SFO**	0.180	0.856	-0.690	0.492	1.840	0.067	0.890	0.375	0.050	0.053
**SLF**	-3.710	0.000	-3.060	0.002	1.030	0.304	0.500	0.620	0.560	0.559
**SS**	-1.670	0.096	0.220	0.826	-0.360	0.720	0.780	0.438	0.120	0.120
**TAP**	-0.900	0.370	-5.310	1.65E-07	0.480	0.631	-0.290	0.772	0.330	0.327

**JHU-ROI**	**RD**									
	**Diagnosis**		**Age**		**[Age-mean(Age)]^2**		**Sex**		**Age-by-Diagnosis**	
	**t**	**p**	**t**	**p**	**t**	**p**	**t**	**p**	**t**	**p**
**ACR**	-2.690	0.007	-6.690	5.75E-11	1.390	0.166	-1.620	0.107	0.820	0.824

**ALIC**	-1.630	0.103	-8.160	2.66E-15	2.900	0.004	-2.390	0.017	0.580	0.580
**Average WM**	-0.170	0.863	-8.210	1.89E-15	2.880	0.004	-1.930	0.054	0.700	0.704
**BCC**	-0.320	0.751	-3.370	0.001	2.980	0.003	-1.670	0.095	0.230	0.234
**CGC**	-0.930	0.350	-9.060	5.00E-16	3.030	0.003	-3.060	0.002	0.930	0.932
**EC**	1.010	0.314	-7.870	2.09E-14	2.530	0.012	-2.290	0.022	0.580	0.583
**FXST**	0.260	0.793	-5.990	3.99E-09	1.570	0.118	-1.320	0.188	0.700	0.701
**GCC**	-2.540	0.011	-2.380	0.018	0.640	0.524	-2.400	0.017	0.890	0.885
**UNC**	-1.150	0.250	-3.640	2.96E-04	2.110	0.035	-1.640	0.102	0.520	0.520
**PCR**	-2.930	0.004	-8.140	3.11E-15	3.840	1.40E-04	-2.340	0.020	0.930	0.927
**PLIC**	-2.850	0.004	-5.010	7.51E-07	0.620	0.537	-2.170	0.030	0.440	0.436
**PTR**	-3.040	0.003	-7.100	4.16E-12	3.800	1.60E-04	-0.660	0.507	0.270	0.271
**RLIC**	-0.780	0.433	-5.910	6.28E-09	2.690	0.007	-1.410	0.159	0.470	0.468
**SCC**	-4.360	0.000	-7.030	6.61E-12	2.110	0.035	-0.880	0.381	0.020	0.022
**SCR**	-1.480	0.141	-6.280	7.13E-10	3.320	0.001	-2.070	0.039	0.660	0.665
**SFO**	-0.690	0.492	-5.220	2.59E-07	1.760	0.078	-1.450	0.147	0.980	0.984
**SLF**	-0.270	0.789	-9.130	5.00E-16	4.240	2.64E-05	-1.700	0.091	0.610	0.605
**SS**	-1.290	0.197	-7.610	1.34E-13	3.980	7.99E-05	-0.410	0.684	0.680	0.679
**TAP**	-2.960	0.003	-6.920	1.40E-11	2.430	0.016	-0.920	0.358	0.880	0.885

Supplementary Table S10. Age analysis - linear model of age. Table shows the residual standard error (**RSE**) of the linear model of age for FA, MD, RD and AD : *DTI-ROI-measure* = $ß_0 + ß_1 * Age + ß_2 * Age^2_{centered}$.

JHU-ROI	22q11.2DS				Healthy Controls			
	RSE FA	RSE MD	RSE RD	RSE AD	RSE FA	RSE MD	RSE RD	RSE AD
ACR	0.028	3.16E-05	3.52E-05	4.75E-05	0.027	3.20E-05	3.45E-05	4.72E-05
ALIC	0.030	3.05E-05	3.16E-05	5.39E-05	0.026	2.79E-05	2.86E-05	4.98E-05
Average WM	0.022	2.54E-05	2.84E-05	3.48E-05	0.020	2.40E-05	2.65E-05	3.34E-05
BCC	0.039	5.09E-05	5.98E-05	7.62E-05	0.032	4.91E-05	5.23E-05	6.91E-05
CGC	0.040	3.18E-05	4.52E-05	6.77E-05	0.040	3.10E-05	4.47E-05	6.08E-05
EC	0.027	2.40E-05	2.92E-05	3.59E-05	0.023	2.24E-05	2.63E-05	3.42E-05
FXST	0.036	3.42E-05	3.46E-05	6.35E-05	0.033	3.02E-05	3.34E-05	5.33E-05
GCC	0.033	4.13E-05	4.43E-05	6.79E-05	0.032	4.05E-05	4.28E-05	6.89E-05
UNC	0.047	3.97E-05	5.26E-05	6.51E-05	0.039	3.60E-05	4.28E-05	6.61E-05
PCR	0.028	3.29E-05	3.61E-05	4.32E-05	0.029	3.14E-05	3.66E-05	4.51E-05
PLIC	0.030	3.24E-05	3.78E-05	5.07E-05	0.028	2.90E-05	3.63E-05	4.20E-05
PTR	0.031	3.92E-05	3.60E-05	8.19E-05	0.029	3.88E-05	3.78E-05	7.48E-05
RLIC	0.029	3.13E-05	3.52E-05	4.96E-05	0.029	2.86E-05	3.47E-05	4.56E-05
SCC	0.028	3.87E-05	3.64E-05	6.97E-05	0.027	3.52E-05	3.38E-05	6.56E-05
SCR	0.030	2.93E-05	3.51E-05	4.51E-05	0.025	2.67E-05	3.07E-05	3.88E-05
SFO	0.037	5.11E-05	5.28E-05	6.51E-05	0.031	3.47E-05	3.43E-05	5.83E-05
SLF	0.025	2.78E-05	3.11E-05	3.99E-05	0.026	2.68E-05	3.02E-05	4.13E-05
SS	0.033	3.55E-05	3.81E-05	6.21E-05	0.029	3.33E-05	3.64E-05	5.76E-05
TAP	0.051	5.37E-05	6.89E-05	7.20E-05	0.046	4.86E-05	6.17E-05	7.04E-05

Supplementary Table S11. Age analysis - non-linear exponential regression model (Poisson fit). This table shows residual standard error (**RSE**) for the non-linear model for FA, MD, RD and AD: *DTI-ROI-measure* $= ß_0 + ß_1 * Age * exp(-ß_2 * Age)$.

JHU-ROI	22q11.2DS				Healthy Controls			
	RSE FA	RSE MD	RSE RD	RSE AD	RSE FA	RSE MD	RSE RD	RSE AD
ACR	0.028	3.16E-05	3.53E-05	4.74E-05	0.027	3.21E-05	3.47E-05	4.72E-05
ALIC	0.030	3.05E-05	3.15E-05	5.40E-05	0.026	2.79E-05	2.86E-05	4.96E-05
Average WM	0.022	2.53E-05	2.84E-05	3.48E-05	0.020	2.40E-05	2.65E-05	3.34E-05
BCC	0.039	5.07E-05	5.97E-05	7.62E-05	0.032	4.91E-05	5.23E-05	6.91E-05
CGC	0.040	3.16E-05	4.52E-05	6.78E-05	0.040	3.11E-05	4.47E-05	6.08E-05
EC	0.027	2.39E-05	2.92E-05	3.56E-05	0.023	2.24E-05	2.63E-05	3.42E-05
FXST	0.036	3.41E-05	3.46E-05	6.35E-05	0.033	3.02E-05	3.33E-05	5.33E-05
GCC	0.033	4.13E-05	4.44E-05	6.79E-05	0.032	4.06E-05	4.28E-05	6.89E-05
UNC	0.047	3.96E-05	5.26E-05	6.48E-05	0.040	3.60E-05	4.28E-05	6.61E-05
PCR	0.028	3.28E-05	3.60E-05	4.32E-05	0.028	3.14E-05	3.65E-05	4.51E-05
PLIC	0.030	3.23E-05	3.78E-05	5.07E-05	0.028	2.90E-05	3.63E-05	4.20E-05
PTR	0.031	3.92E-05	3.60E-05	8.19E-05	0.029	3.88E-05	3.76E-05	7.49E-05
RLIC	0.029	3.12E-05	3.52E-05	4.96E-05	0.029	2.87E-05	3.47E-05	4.56E-05
SCC	0.028	3.87E-05	3.64E-05	6.97E-05	0.027	3.54E-05	3.40E-05	6.57E-05
SCR	0.030	2.92E-05	3.51E-05	4.51E-05	0.025	2.67E-05	3.07E-05	3.88E-05
SFO	0.037	5.11E-05	5.28E-05	6.51E-05	0.031	3.46E-05	3.42E-05	5.83E-05
SLF	0.025	2.78E-05	3.10E-05	3.99E-05	0.026	2.69E-05	3.02E-05	4.13E-05
SS	0.033	3.55E-05	3.80E-05	6.21E-05	0.029	3.33E-05	3.62E-05	5.76E-05
TAP	0.052	5.37E-05	6.90E-05	7.20E-05	0.046	4.86E-05	6.17E-05	7.03E-05

Supplementary Table S12. Linear vs. Non-linear age models. T-test results comparing the RSE of the linear model of age vs the non-linear Poisson model of age. Linear model: DTI-ROI-measure = $ß_0$ + $ß_1$*Age*exp(-$ß_2$*Age). Non-linear Poisson model: DTI-ROI-measure = $ß_0$ + $ß_1$*Age*exp(-$ß_2$*Age).

Group / DTI-measure	22q11.2 Deletion Syndrome p-value (α=0.05)	Healthy Controls p-value (α=0.05)
**FA**	0.132	0.850
**MD**	0.001	0.083
**RD**	0.029	0.359
**AD**	0.289	0.792

Supplementary Table S13. Age of FA peak and MD, RD and AD minima for healthy control group. The peak FA and minima of MD, RD, AD of the Poisson fits of age were calculated as described in Lebel et al. 2012, by calculating the derivative of the fit: FA_{peak} (or $MD_{minimum}$) = 1/$ß_2$. The full model is: *DTI-ROI-measure* = $ß_0 + ß_1 * Age * exp(-ß_2 * Age)$. P-values and standard errors (SE) of the fitted $ß_2$ coefficient are also shown. Colored cells indicate the significant p-values below the threshold that controls the false discovery rate (FDR).

JHU-ROI	Healthy Controls											
	FA			MD			RD			AD		
	peak	p	SE	min	p	SE	min	p	SE	min	p	SE
ACR	21.15	9.8E-26	4.0E-03	36.32	5.7E-09	4.6E-03	28.65	5.6E-16	4.0E-03	97.71	2.5E-01	8.8E-03
ALIC	43.96	3.0E-07	4.3E-03	70.85	5.3E-02	7.3E-03	47.51	3.4E-06	4.4E-03	15.98	3.6E-08	1.1E-02
Aver. WM	25.58	2.8E-30	3.0E-03	31.76	2.2E-18	3.3E-03	28.46	2.8E-27	2.9E-03	51.71	3.5E-02	9.1E-03
BCC	23.69	8.9E-14	5.4E-03	26.21	7.0E-07	7.5E-03	24.90	1.2E-10	6.0E-03	38.27	4.0E-01	3.1E-02
CGC	31.64	3.2E-15	3.8E-03	33.19	8.0E-14	3.8E-03	33.40	2.5E-16	3.4E-03	24.36	9.5E-04	1.2E-02
EC	29.67	5.6E-14	4.2E-03	36.28	1.7E-09	4.4E-03	33.30	1.3E-14	3.7E-03	76.20	5.3E-01	2.1E-02
FXST	21.02	1.7E-12	6.4E-03	38.38	5.1E-06	5.6E-03	31.84	5.3E-09	5.2E-03	67.95	2.7E-01	1.3E-02
GCC	18.79	6.2E-12	7.4E-03	39.06	3.8E-03	8.8E-03	22.99	2.9E-08	7.6E-03	169.94	6.2E-01	1.2E-02
UNC	24.16	1.0E-10	6.1E-03	31.15	2.7E-07	6.1E-03	26.91	6.1E-10	5.8E-03	50.53	3.6E-01	2.2E-02
PCR	22.83	9.4E-29	3.5E-03	31.06	1.4E-18	3.4E-03	27.26	9.5E-26	3.1E-03	54.34	2.5E-02	8.2E-03
PLIC	26.39	7.0E-06	8.3E-03	42.24	4.2E-07	4.6E-03	31.05	8.9E-07	6.4E-03	62.90	2.1E-03	5.1E-03
PTR	21.60	4.4E-28	3.7E-03	26.31	5.2E-19	3.9E-03	24.52	4.3E-30	3.1E-03	29.55	1.6E-03	1.1E-02
RLIC	22.93	3.0E-16	5.0E-03	30.92	5.3E-12	4.5E-03	26.69	1.5E-15	4.4E-03	52.94	8.5E-02	1.1E-02
SCC	25.85	6.1E-15	4.7E-03	38.27	9.8E-08	4.8E-03	28.84	6.1E-18	3.7E-03	85.67	3.7E-01	1.3E-02
SCR	23.60	8.8E-22	4.0E-03	29.39	5.6E-16	3.9E-03	26.26	8.2E-22	3.6E-03	48.75	4.2E-02	1.0E-02
SFO	26.90	1.5E-16	4.2E-03	26.11	1.3E-14	4.7E-03	26.19	1.5E-21	3.7E-03	25.11	5.0E-02	2.0E-02
SLF	25.96	4.4E-26	3.3E-03	29.91	2.1E-20	3.3E-03	29.04	1.9E-25	3.0E-03	36.58	1.3E-02	1.1E-02
SS	23.68	1.4E-33	3.0E-03	26.86	7.8E-15	4.5E-03	26.13	5.1E-28	3.1E-03	2.97	7.9E-01	1.3E+00
TAP	29.38	3.1E-13	4.4E-03	26.95	1.4E-30	2.8E-03	28.43	6.4E-21	3.4E-03	22.72	2.3E-22	4.1E-03

Supplementary Table S14. Age of FA peak and MD, RD and AD minima for 22q11DS. The peak FA and minima of MD, RD, AD of the Poisson fits of age were calculated as described in Lebel et al. 2012, by calculating the derivative of the fit: FA_{peak} (or $MD_{minimum}$) = $1/ß_2$. The full model is: *DTI-ROI-measure* = $ß_0 + ß_1*Age*exp(-ß_2*Age)$. P-values and standard errors (SE) of the fitted $ß_2$ coefficient are also shown. Colored cells indicate the significant p-values below the threshold that controls the false discovery rate (FDR), indicating that the $ß_2$ coefficient is significantly different from zero.

JHU-ROI	22q11.2DS											
	FA			MD			RD			AD		
	peak	p	SE	min	p	SE	min	p	SE	min	p	SE
ACR	38.03	1.0E-01	1.6E-02	38.80	2.9E-05	6.1E-03	39.52	1.2E-03	7.7E-03	37.45	3.4E-04	7.4E-03
ALIC	44.69	1.2E-03	6.8E-03	56.35	3.7E-02	8.5E-03	45.94	4.7E-04	6.1E-03	8.38	2.1E-01	9.5E-02
Aver. WM	33.00	2.5E-06	6.3E-03	36.04	3.8E-08	4.9E-03	34.44	1.4E-08	5.0E-03	49.71	5.0E-02	1.0E-02
BCC	23.86	4.2E-10	6.5E-03	28.50	1.1E-07	6.4E-03	25.46	2.2E-10	6.0E-03	-104.68	7.8E-01	3.4E-02
CGC	30.55	9.4E-09	5.5E-03	27.92	1.1E-15	4.2E-03	30.94	4.0E-13	4.2E-03	43.77	5.4E-01	3.7E-02
EC	55.63	6.7E-02	9.8E-03	32.70	1.5E-09	4.9E-03	40.13	4.1E-05	6.0E-03	20.95	2.2E-17	5.3E-03
FXST	58.49	3.0E-01	1.6E-02	57.37	4.8E-02	8.8E-03	48.75	1.2E-02	8.1E-03	327.09	8.9E-01	2.2E-02
GCC	18.85	4.2E-08	9.4E-03	34.08	6.3E-03	1.1E-02	24.17	4.8E-04	1.2E-02	73.88	3.6E-01	1.5E-02
UNC	-147.09	8.8E-01	4.6E-02	25.33	7.7E-08	7.1E-03	48.29	3.3E-01	2.1E-02	19.34	3.6E-19	5.4E-03
PCR	29.84	5.4E-08	6.0E-03	33.18	1.5E-09	4.8E-03	32.38	1.4E-09	4.9E-03	35.26	1.8E-04	7.5E-03
PLIC	30.83	3.2E-03	1.1E-02	31.62	2.2E-04	8.4E-03	33.37	4.8E-03	1.1E-02	40.11	1.2E-01	1.6E-02
PTR	23.48	2.9E-07	8.1E-03	43.54	8.7E-03	8.7E-03	29.01	1.2E-07	6.3E-03	1422.14	9.7E-01	1.9E-02
RLIC	28.74	3.6E-07	6.7E-03	31.22	7.4E-08	5.8E-03	29.02	2.0E-10	5.2E-03	54.64	4.1E-01	2.2E-02
SCC	17.23	3.4E-12	8.0E-03	42.11	2.5E-02	1.1E-02	29.06	6.4E-04	1.0E-02	131.68	6.4E-01	1.6E-02
SCR	37.48	2.0E-02	1.1E-02	34.48	6.1E-08	5.2E-03	34.79	2.1E-05	6.6E-03	36.28	8.3E-04	8.2E-03
SFO	75.71	4.8E-01	1.9E-02	83.98	3.7E-01	1.3E-02	85.05	4.0E-01	1.4E-02	277.85	8.5E-01	1.9E-02
SLF	34.69	3.6E-07	5.5E-03	33.57	1.1E-10	4.4E-03	34.23	7.8E-11	4.3E-03	33.05	3.3E-03	1.0E-02
SS	30.26	7.0E-07	6.5E-03	36.31	2.6E-05	6.4E-03	31.71	3.3E-10	4.8E-03	-36.34	5.1E-01	4.2E-02
TAP	35.71	1.9E-03	8.9E-03	41.02	2.9E-04	6.6E-03	40.22	9.0E-04	7.4E-03	44.50	1.2E-01	1.4E-02

Supplementary Table S15. Group Differences in Peak FA and Minimum Diffusivity Values- Non-linear Poisson fits. Top Panel: Differences in mean peak FA/minimum MD, RD, AD across all ROIs between 22q11DS participants and Healthy Controls. Bottom Panel: Differences in mean percent FA decrease (after peak age) and mean percent change of increase (after minimum age) of MD, AD and RD (alpha=0.05).

Peak FA / Minimum MD, RD, AD differences				
DTI Measure	FA	MD	RD	AD
22q11DS Mean Peak/Minimum	30.48	34.40	35.07	38.25
Healthy Controls Mean Peak/ Minimum	24.30	32.24	28.04	46.90
Differences in Mean	t = -3.032<br>p-value = 0.007	t = -1.230<br>p-value = 0.227	t = -3.784<br>p-value = 0.001	t = 1.079<br>p-value = 0.289
**Differences in percent changes of FA decrease; MD, RD, AD increase**				
DTI Measure	FA	MD	RD	AD
Mean percent change 22q11DS	-0.89	0.56	5.25	0.092
Mean percent change Healthy Controls	-3.94	1.66	1.37	0.039
Mean percent change differences	t = -6.05<br>CI = (-3.93, -2.16)	t = 2.96<br>CI = (0.39, 1.81)	t = 4.86<br>CI = (2.44, 5.31)	t = -0.59<br>CI = (-0.21, 0.10)

*p<.05

Supplementary Table S16. 22q11DS+Psychosis vs. 22q11DS-No Psychosis. Results of the local non-parametric ANCOVA comparing 22q11DS subjects with psychotic disorder (N=35) vs. those with no history of psychotic symptoms (N=191). Twenty-five design points (or age bands) were selected. The critical p-value is 0.0056. Tables show results for DTI indices that significantly differed between 22q-Psychosis vs. 22q-No Psychosis for at least one design point: **A)** Axial Diffusivity (AD) in the Anterior Limb of the Internal Capsule (ALIC); **B)** AD in the Cingulum of the Cingulate Gyrus (CGC); **C)** AD in the Posterior Thalamic Radiation (PTR); **D)** AD in the Superior Longitudinal Fasciculus (SLF); **E)** AD in the Sagittal Stratum (SS); **F)** Mean Diffusivity (MD) in the Genu of Corpus Callosum (GCC); **G)** MD in the Posterior Limb of the Internal Capsule (PLIC); **H)** Radial Diffusivity (RD) in the Genu of the Corpus Callosum (GCC). Color filled cells indicate the design points that are significantly different between groups. N1 and N2 refer to the sample sizes at each design point; DIF = estimated difference between the means; TEST = resulting test statistic; SE = standard error; ci.low = lower bound of the confidence interval; ci.hi = upper bound of the confidence interval.

A)

Age (Design Points)	N1	N2	DIF	TEST	SE	ci.low	ci.hi	p-value	Effect Size
12.83	87	12	-4.34E-05	1.90	2.28E-05	-1.98E-05	1.07E-04	0.0876	-0.34
13.47	101	12	-4.29E-05	1.90	2.26E-05	-1.97E-05	1.05E-04	0.0892	-0.36
14.11	111	12	-4.14E-05	1.85	2.24E-05	-2.07E-05	1.04E-04	0.0979	-0.32
14.75	117	15	-6.59E-05	2.98	2.21E-05	4.65E-06	1.27E-04	0.0134	-0.50
15.39	121	19	-4.36E-05	2.02	2.16E-05	-1.63E-05	1.04E-04	0.0618	-0.33
16.04	131	19	-4.43E-05	2.05	2.16E-05	-1.55E-05	1.04E-04	0.0579	-0.35
16.68	132	18	-5.13E-05	2.39	2.15E-05	-8.24E-06	1.11E-04	0.0318	-0.40
17.32	134	18	-5.03E-05	2.35	2.15E-05	-9.08E-06	1.10E-04	0.0344	-0.44
17.96	132	20	-5.50E-05	2.68	2.05E-05	-1.73E-06	1.12E-04	0.0177	-0.45
18.60	131	22	-5.94E-05	3.18	1.87E-05	7.64E-06	1.11E-04	0.0052	-0.47
19.24	126	23	-5.59E-05	3.14	1.78E-05	6.59E-06	1.05E-04	0.0052	-0.49
19.88	123	24	-5.61E-05	3.33	1.69E-05	9.46E-06	1.03E-04	0.0030	-0.48
20.52	112	25	-5.79E-05	3.44	1.68E-05	1.12E-05	1.04E-04	0.0024	-0.54
21.16	106	28	-4.94E-05	2.91	1.70E-05	2.36E-06	9.65E-05	0.0073	-0.47
21.81	106	27	-4.82E-05	2.71	1.78E-05	-1.07E-06	9.74E-05	0.0123	-0.41
22.45	96	27	-4.69E-05	2.61	1.80E-05	-2.88E-06	9.67E-05	0.0151	-0.43

Age (Design Points)	N1	N2	DIF	TEST	SE	ci.low	ci.hi	p-value	Effect Size
12.83	87	12	-4.34E-05	1.90	2.28E-05	-1.98E-05	1.07E-04	0.0876	-0.34
13.47	101	12	-4.29E-05	1.90	2.26E-05	-1.97E-05	1.05E-04	0.0892	-0.36
14.11	111	12	-4.14E-05	1.85	2.24E-05	-2.07E-05	1.04E-04	0.0979	-0.32
14.75	117	15	-6.59E-05	2.98	2.21E-05	4.65E-06	1.27E-04	0.0134	-0.50
15.39	121	19	-4.36E-05	2.02	2.16E-05	-1.63E-05	1.04E-04	0.0618	-0.33
16.04	131	19	-4.43E-05	2.05	2.16E-05	-1.55E-05	1.04E-04	0.0579	-0.35
16.68	132	18	-5.13E-05	2.39	2.15E-05	-8.24E-06	1.11E-04	0.0318	-0.40
17.32	134	18	-5.03E-05	2.35	2.15E-05	-9.08E-06	1.10E-04	0.0344	-0.44
17.96	132	20	-5.50E-05	2.68	2.05E-05	-1.73E-06	1.12E-04	0.0177	-0.45
18.60	131	22	-5.94E-05	3.18	1.87E-05	7.64E-06	1.11E-04	0.0052	-0.47
19.24	126	23	-5.59E-05	3.14	1.78E-05	6.59E-06	1.05E-04	0.0052	-0.49
19.88	123	24	-5.61E-05	3.33	1.69E-05	9.46E-06	1.03E-04	0.0030	-0.48
20.52	112	25	-5.79E-05	3.44	1.68E-05	1.12E-05	1.04E-04	0.0024	-0.54
21.16	106	28	-4.94E-05	2.91	1.70E-05	2.36E-06	9.65E-05	0.0073	-0.47
21.81	106	27	-4.82E-05	2.71	1.78E-05	-1.07E-06	9.74E-05	0.0123	-0.41
22.45	96	27	-4.69E-05	2.61	1.80E-05	-2.88E-06	9.67E-05	0.0151	-0.43
23.09	82	25	-5.30E-05	2.65	2.00E-05	-2.41E-06	1.08E-04	0.0151	-0.48
23.73	74	25	-5.62E-05	2.77	2.03E-05	-2.10E-09	1.12E-04	0.0113	-0.50
24.37	62	24	-5.73E-05	2.85	2.01E-05	1.68E-06	1.13E-04	0.0084	-0.46
25.01	56	24	-5.64E-05	2.78	2.02E-05	3.13E-07	1.12E-04	0.0098	-0.47
25.65	49	22	-5.54E-05	2.56	2.16E-05	-4.43E-06	1.15E-04	0.0178	-0.48
26.29	38	21	-6.29E-05	2.84	2.21E-05	1.59E-06	1.24E-04	0.0102	-0.64
26.93	35	21	-6.90E-05	3.25	2.12E-05	1.03E-05	1.28E-04	0.0035	-0.68
27.58	26	20	-6.30E-05	2.67	2.36E-05	-2.23E-06	1.28E-04	0.0137	-0.66
28.22	21	20	-6.07E-05	2.44	2.49E-05	-8.26E-06	1.30E-04	0.0232	-0.62

B)

Age (Design Points)	N1	N2	DIF	TEST	SE	ci.low	ci.hi	p-value	Effect Size
12.83	87	12	-3.34E-05	1.78	1.88E-05	-1.87E-05	8.54E-05	0.1012	-0.31
13.47	101	12	-3.43E-05	1.87	1.83E-05	-1.65E-05	8.50E-05	0.0886	-0.30
14.11	111	12	-3.44E-05	1.89	1.82E-05	-1.61E-05	8.48E-05	0.0869	-0.33
14.75	117	15	-4.71E-05	2.86	1.65E-05	1.45E-06	9.27E-05	0.0137	-0.48
15.39	121	19	-3.53E-05	2.04	1.73E-05	-1.26E-05	8.32E-05	0.0559	-0.34
16.04	131	19	-3.81E-05	2.20	1.73E-05	-9.91E-06	8.60E-05	0.0408	-0.38
16.68	132	18	-4.59E-05	2.69	1.71E-05	-1.31E-06	9.31E-05	0.0147	-0.41
17.32	134	18	-4.45E-05	2.59	1.72E-05	-3.12E-06	9.22E-05	0.0180	-0.45
17.96	132	20	-4.90E-05	2.71	1.81E-05	-9.66E-07	9.90E-05	0.0138	-0.45
18.60	131	22	-4.95E-05	2.91	1.70E-05	2.42E-06	9.65E-05	0.0072	-0.40
19.24	126	23	-4.76E-05	2.93	1.63E-05	2.57E-06	9.26E-05	0.0064	-0.41
19.88	123	24	-4.80E-05	3.09	1.55E-05	4.99E-06	9.10E-05	0.0039	-0.45
20.52	112	25	-4.72E-05	2.95	1.60E-05	2.93E-06	9.16E-05	0.0057	-0.42
21.16	106	28	-4.54E-05	3.02	1.50E-05	3.78E-06	8.71E-05	0.0042	-0.38
21.81	106	27	-4.53E-05	3.00	1.51E-05	3.43E-06	8.72E-05	0.0046	-0.37
22.45	96	27	-4.37E-05	2.76	1.58E-05	-1.06E-07	8.75E-05	0.0086	-0.39
23.09	82	25	-4.76E-05	2.78	1.71E-05	2.06E-07	9.50E-05	0.0090	-0.43
23.73	74	25	-5.19E-05	2.94	1.77E-05	3.06E-06	1.01E-04	0.0058	-0.42
24.37	62	24	-6.26E-05	3.50	1.79E-05	1.30E-05	1.12E-04	0.0012	-0.52
25.01	56	24	-6.69E-05	3.61	1.85E-05	1.57E-05	1.18E-04	0.0009	-0.54
25.65	49	22	-6.54E-05	3.20	2.04E-05	8.86E-06	1.22E-04	0.0031	-0.47
26.29	38	21	-6.60E-05	3.19	2.07E-05	8.75E-06	1.23E-04	0.0032	-0.54
26.93	35	21	-6.01E-05	2.69	2.23E-05	-1.64E-06	1.22E-04	0.0114	-0.46
27.58	26	20	-6.80E-05	3.15	2.16E-05	8.26E-06	1.28E-04	0.0042	-0.57
28.22	21	20	-6.21E-05	2.88	2.16E-05	2.38E-06	1.22E-04	0.0085	-0.51

C)

Age (Design Points)	N1	N2	DIF	TEST	SE	ci.low	ci.hi	p-value	Effect Size
12.83	87	12	-4.23E-05	1.85	2.29E-05	-2.10E-05	1.06E-04	0.0949	-0.33
13.47	101	12	-4.52E-05	1.96	2.31E-05	-1.87E-05	1.09E-04	0.0785	-0.35
14.11	111	12	-4.19E-05	1.86	2.25E-05	-2.04E-05	1.04E-04	0.0951	-0.34
14.75	117	15	-5.51E-05	2.92	1.89E-05	2.79E-06	1.07E-04	0.0136	-0.48
15.39	121	19	-4.83E-05	2.85	1.70E-05	1.38E-06	9.53E-05	0.0104	-0.47
16.04	131	19	-5.03E-05	2.97	1.70E-05	3.38E-06	9.73E-05	0.0081	-0.48
16.68	132	18	-5.32E-05	3.21	1.66E-05	7.25E-06	9.91E-05	0.0051	-0.56
17.32	134	18	-5.07E-05	3.07	1.65E-05	4.94E-06	9.64E-05	0.0070	-0.53
17.96	132	20	-4.59E-05	3.04	1.51E-05	4.16E-06	8.76E-05	0.0064	-0.51
18.60	131	22	-4.72E-05	3.39	1.39E-05	8.72E-06	8.58E-05	0.0021	-0.48
19.24	126	23	-4.17E-05	3.13	1.33E-05	4.86E-06	7.86E-05	0.0037	-0.46
19.88	123	24	-4.01E-05	2.87	1.40E-05	1.39E-06	7.88E-05	0.0073	-0.45
20.52	112	25	-4.32E-05	3.26	1.32E-05	6.56E-06	7.99E-05	0.0024	-0.46
21.16	106	28	-3.85E-05	2.84	1.36E-05	9.07E-07	7.62E-05	0.0069	-0.38
21.81	106	27	-3.53E-05	2.53	1.39E-05	-3.25E-06	7.38E-05	0.0154	-0.34
22.45	96	27	-3.06E-05	2.14	1.43E-05	-9.07E-06	7.04E-05	0.0392	-0.30
23.09	82	25	-3.05E-05	2.03	1.50E-05	-1.12E-05	7.22E-05	0.0514	0.32
23.73	74	25	-2.47E-05	1.63	1.51E-05	-1.71E-05	6.65E-05	0.1124	-0.28
24.37	62	24	-2.73E-05	1.72	1.58E-05	-1.66E-05	7.11E-05	0.0925	-0.31
25.01	56	24	-2.74E-05	1.67	1.64E-05	-1.80E-05	7.28E-05	0.1027	-0.30
25.65	49	22	-2.63E-05	1.41	1.86E-05	-2.52E-05	7.77E-05	0.1661	-0.26
26.29	38	21	-1.25E-05	0.69	1.83E-05	-3.80E-05	6.31E-05	0.4970	-0.14
26.93	35	21	-1.53E-05	0.78	1.95E-05	-3.87E-05	6.93E-05	0.4388	-0.15
27.58	26	20	-9.58E-06	0.43	2.21E-05	-5.16E-05	7.08E-05	0.6685	-0.12
28.22	21	20	-9.34E-06	0.41	2.28E-05	-5.37E-05	7.23E-05	0.6855	-0.10

D)

Age (Design Points)	N1	N2	DIF	TEST	SE	ci.low	ci.hi	p-value	Effect Size
12.83	87	12	-2.63E-05	1.49	1.77E-05	-2.27E-05	7.54E-05	0.1673	-0.32
13.47	101	12	-2.85E-05	1.63	1.75E-05	-2.00E-05	7.69E-05	0.1353	-0.33
14.11	111	12	-2.90E-05	1.68	1.73E-05	-1.87E-05	7.68E-05	0.1257	-0.37
14.75	117	15	-3.45E-05	2.46	1.40E-05	-4.33E-06	7.34E-05	0.0290	-0.44
15.39	121	19	-1.94E-05	1.45	1.34E-05	-1.76E-05	5.65E-05	0.1635	-0.26
16.04	131	19	-2.08E-05	1.55	1.34E-05	-1.62E-05	5.78E-05	0.1378	-0.26
16.68	132	18	-2.76E-05	2.09	1.32E-05	-8.93E-06	6.41E-05	0.0524	-0.38
17.32	134	18	-2.61E-05	1.99	1.31E-05	-1.03E-05	6.25E-05	0.0640	-0.36
17.96	132	20	-3.03E-05	2.56	1.18E-05	-2.49E-06	6.31E-05	0.0197	-0.44
18.60	131	22	-2.87E-05	2.67	1.08E-05	-1.11E-06	5.84E-05	0.0132	-0.40
19.24	126	23	-2.70E-05	2.58	1.05E-05	-1.94E-06	5.60E-05	0.0148	-0.36
19.88	123	24	-2.69E-05	2.71	9.95E-06	-6.23E-07	5.45E-05	0.0105	-0.43
20.52	112	25	-3.00E-05	3.12	9.60E-06	3.42E-06	5.66E-05	0.0032	-0.43
21.16	106	28	-2.39E-05	2.15	1.11E-05	-6.82E-06	5.47E-05	0.0379	-0.33
21.81	106	27	-2.23E-05	1.96	1.14E-05	-9.14E-06	5.38E-05	0.0574	-0.29
22.45	96	27	-2.23E-05	1.99	1.12E-05	-8.80E-06	5.34E-05	0.0559	-0.28
23.09	82	25	-2.41E-05	2.13	1.13E-05	-7.19E-06	5.54E-05	0.0419	-0.31
23.73	74	25	-2.16E-05	1.85	1.17E-05	-1.08E-05	5.40E-05	0.0743	-0.29
24.37	62	24	-2.34E-05	1.86	1.26E-05	-1.15E-05	5.82E-05	0.0726	-0.33
25.01	56	24	-2.09E-05	1.64	1.27E-05	-1.44E-05	5.62E-05	0.1102	-0.28
25.65	49	22	-2.27E-05	1.81	1.26E-05	-1.21E-05	5.75E-05	0.0805	-0.29
26.29	38	21	-2.02E-05	1.51	1.33E-05	-1.67E-05	5.71E-05	0.1413	-0.29
26.93	35	21	-1.77E-05	1.22	1.45E-05	-2.24E-05	5.78E-05	0.2326	-0.24
27.58	26	20	-1.04E-05	0.65	1.61E-05	-3.42E-05	5.50E-05	0.5231	-0.16
28.22	21	20	-6.67E-06	0.38	1.74E-05	-4.14E-05	5.48E-05	0.7045	-0.07

E)

Age (Design Points)	N1	N2	DIF	TEST	SE	ci.low	ci.hi	p-value	Effect Size
12.83	87	12	-1.86E-05	1.17	1.59E-05	-2.55E-05	6.27E-05	0.2668	-0.24
13.47	101	12	-2.21E-05	1.42	1.56E-05	-2.11E-05	6.54E-05	0.1855	-0.27
14.11	111	12	-2.19E-05	1.43	1.53E-05	-2.04E-05	6.42E-05	0.1831	-0.28
14.75	117	15	-3.54E-05	2.26	1.57E-05	-7.97E-06	7.87E-05	0.0455	-0.37
15.39	121	19	-3.66E-05	2.47	1.48E-05	-4.39E-06	7.76E-05	0.0238	-0.40
16.04	131	19	-3.90E-05	2.64	1.48E-05	-1.83E-06	7.99E-05	0.0168	-0.39
16.68	132	18	-4.47E-05	3.12	1.43E-05	5.09E-06	8.43E-05	0.0066	-0.49
17.32	134	18	-4.56E-05	3.15	1.45E-05	5.51E-06	8.58E-05	0.0059	-0.51
17.96	132	20	-4.94E-05	3.30	1.50E-05	8.00E-06	9.08E-05	0.0045	-0.57
18.60	131	22	-4.81E-05	3.31	1.46E-05	7.81E-06	8.84E-05	0.0033	-0.44
19.24	126	23	-4.55E-05	3.28	1.39E-05	7.05E-06	8.39E-05	0.0031	-0.45
19.88	123	24	-4.60E-05	3.48	1.32E-05	9.42E-06	8.25E-05	0.0017	-0.46
20.52	112	25	-4.76E-05	3.49	1.36E-05	9.88E-06	8.53E-05	0.0018	-0.51
21.16	106	28	-4.34E-05	3.27	1.33E-05	6.61E-06	8.03E-05	0.0027	-0.47
21.81	106	27	-4.15E-05	2.98	1.39E-05	2.99E-06	8.00E-05	0.0057	-0.40
22.45	96	27	-4.29E-05	3.03	1.42E-05	3.66E-06	8.21E-05	0.0050	-0.44
23.09	82	25	-4.82E-05	3.35	1.44E-05	8.33E-06	8.81E-05	0.0023	-0.49
23.73	74	25	-4.82E-05	3.24	1.49E-05	7.04E-06	8.93E-05	0.0028	-0.46
24.37	62	24	-5.56E-05	3.50	1.59E-05	1.16E-05	9.95E-05	0.0013	-0.53
25.01	56	24	-5.19E-05	3.17	1.64E-05	6.59E-06	9.73E-05	0.0031	-0.48
25.65	49	22	-5.49E-05	3.41	1.61E-05	1.04E-05	9.93E-05	0.0016	-0.51
26.29	38	21	-5.01E-05	2.90	1.73E-05	2.28E-06	9.80E-05	0.0068	-0.47
26.93	35	21	-4.69E-05	2.45	1.91E-05	-6.08E-06	1.00E-04	0.0210	-0.42
27.58	26	20	-2.96E-05	1.49	1.99E-05	-2.56E-05	8.48E-05	0.1507	-0.32
28.22	21	20	-2.81E-05	1.36	2.07E-05	-2.93E-05	8.55E-05	0.1881	-0.29

F)

Age (Design Points)	N1	N2	DIF	TEST	SE	ci.low	ci.hi	p-value	Effect Size
12.83	87	12	-4.08E-05	2.90	1.41E-05	1.79E-06	7.99E-05	0.0099	-0.45
13.47	101	12	-4.05E-05	2.94	1.38E-05	2.34E-06	7.86E-05	0.0096	-0.44
14.11	111	12	-3.99E-05	2.92	1.37E-05	2.05E-06	7.77E-05	0.0102	-0.44
14.75	117	15	-4.32E-05	2.80	1.55E-05	4.44E-07	8.60E-05	0.0145	-0.44
15.39	121	19	-3.11E-05	2.13	1.46E-05	-9.40E-06	7.17E-05	0.0459	-0.34
16.04	131	19	-3.10E-05	2.16	1.44E-05	-8.74E-06	7.07E-05	0.0438	-0.32
16.68	132	18	-3.37E-05	2.65	1.27E-05	-1.56E-06	6.89E-05	0.0154	-0.42
17.32	134	18	-3.26E-05	2.53	1.29E-05	-3.05E-06	6.83E-05	0.0193	-0.44
17.96	132	20	-3.45E-05	2.62	1.32E-05	-1.94E-06	7.09E-05	0.0163	-0.39
18.60	131	22	-4.13E-05	2.72	1.52E-05	-8.06E-07	8.35E-05	0.0132	-0.40
19.24	126	23	-3.91E-05	2.68	1.46E-05	-1.24E-06	7.94E-05	0.0133	-0.43
19.88	123	24	-4.23E-05	3.01	1.41E-05	3.35E-06	8.13E-05	0.0060	-0.46
20.52	112	25	-4.38E-05	3.13	1.40E-05	5.02E-06	8.26E-05	0.0043	-0.49
21.16	106	28	-4.26E-05	3.23	1.32E-05	6.12E-06	7.90E-05	0.0027	-0.47
21.81	106	27	-4.40E-05	3.20	1.37E-05	5.99E-06	8.20E-05	0.0030	-0.43
22.45	96	27	-4.34E-05	3.25	1.33E-05	6.47E-06	8.04E-05	0.0027	-0.47
23.09	82	25	-4.49E-05	3.24	1.39E-05	6.56E-06	8.33E-05	0.0030	-0.49
23.73	74	25	-4.61E-05	3.13	1.47E-05	5.29E-06	8.68E-05	0.0036	-0.48
24.37	62	24	-4.78E-05	3.24	1.48E-05	6.96E-06	8.87E-05	0.0027	-0.51
25.01	56	24	-5.07E-05	3.38	1.50E-05	9.15E-06	9.23E-05	0.0018	-0.49
25.65	49	22	-5.08E-05	3.21	1.58E-05	7.00E-06	9.46E-05	0.0030	-0.49
26.29	38	21	-5.28E-05	2.99	1.77E-05	3.90E-06	1.02E-04	0.0054	-0.50
26.93	35	21	-5.46E-05	3.01	1.82E-05	4.32E-06	1.05E-04	0.0055	-0.52
27.58	26	20	-6.35E-05	3.21	1.98E-05	8.78E-06	1.18E-04	0.0037	-0.60
28.22	21	20	-4.85E-05	2.49	1.95E-05	-5.35E-06	1.02E-04	0.0205	-0.52

G)

Age (Design Points)	N1	N2	DIF	TEST	SE	ci.low	ci.hi	p-value	Effect Size
12.83	87	12	-3.49E-05	2.23	1.56E-05	-8.35E-06	7.81E-05	0.0486	-0.43
13.47	101	12	-3.10E-05	2.02	1.54E-05	-1.15E-05	7.35E-05	0.0721	-0.38
14.11	111	12	-3.00E-05	1.99	1.51E-05	-1.18E-05	7.18E-05	0.0779	-0.42
14.75	117	15	-3.20E-05	2.22	1.44E-05	-7.85E-06	7.18E-05	0.0490	-0.43
15.39	121	19	-2.10E-05	1.71	1.22E-05	-1.29E-05	5.48E-05	0.1045	-0.30
16.04	131	19	-2.26E-05	1.85	1.23E-05	-1.13E-05	5.66E-05	0.0819	-0.32
16.68	132	18	-2.74E-05	2.27	1.20E-05	-5.97E-06	6.07E-05	0.0370	-0.37
17.32	134	18	-2.85E-05	2.38	1.20E-05	-4.67E-06	6.16E-05	0.0304	-0.41
17.96	132	20	-3.26E-05	2.72	1.20E-05	-6.30E-07	6.58E-05	0.0153	-0.45
18.60	131	22	-3.18E-05	2.81	1.13E-05	4.42E-07	6.31E-05	0.0106	-0.41
19.24	126	23	-2.69E-05	2.50	1.08E-05	-2.94E-06	5.67E-05	0.0197	-0.39
19.88	123	24	-2.75E-05	2.71	1.01E-05	-5.93E-07	5.56E-05	0.0119	-0.44
20.52	112	25	-3.35E-05	3.24	1.03E-05	4.91E-06	6.20E-05	0.0033	-0.49
21.16	106	28	-2.83E-05	2.88	9.85E-06	1.05E-06	5.56E-05	0.0070	-0.41
21.81	106	27	-2.67E-05	2.71	9.84E-06	-6.00E-07	5.39E-05	0.0110	-0.39
22.45	96	27	-2.31E-05	2.35	9.83E-06	-4.10E-06	5.03E-05	0.0245	-0.32
23.09	82	25	-2.62E-05	2.46	1.06E-05	-3.28E-06	5.56E-05	0.0201	-0.40
23.73	74	25	-2.87E-05	2.67	1.08E-05	-1.08E-06	5.85E-05	0.0122	-0.43
24.37	62	24	-3.48E-05	3.04	1.15E-05	3.09E-06	6.65E-05	0.0044	-0.47
25.01	56	24	-3.26E-05	2.83	1.15E-05	6.86E-07	6.46E-05	0.0076	-0.48
25.65	49	22	-3.23E-05	2.74	1.18E-05	-2.91E-07	6.49E-05	0.0097	-0.46
26.29	38	21	-2.73E-05	2.27	1.20E-05	-5.96E-06	6.06E-05	0.0313	-0.41
26.93	35	21	-2.74E-05	2.15	1.27E-05	-7.88E-06	6.26E-05	0.0402	-0.38
27.58	26	20	-1.64E-05	1.03	1.58E-05	-2.75E-05	6.02E-05	0.3116	-0.22
28.22	21	20	-1.45E-05	0.93	1.56E-05	-2.86E-05	5.76E-05	0.3617	-0.21

H)

Age (Design Points)	N1	N2	DIF	TEST	SE	ci.low	ci.hi	p-value	Effect Size
12.83	87	12	-3.25E-05	2.28	1.43E-05	-7.04E-06	7.21E-05	0.0290	-0.37
13.47	101	12	-3.67E-05	2.70	1.36E-05	-9.15E-07	7.44E-05	0.0109	-0.44
14.11	111	12	-3.57E-05	2.71	1.32E-05	-7.88E-07	7.21E-05	0.0111	-0.42
14.75	117	15	-3.77E-05	2.65	1.42E-05	-1.64E-06	7.70E-05	0.0138	-0.37
15.39	121	19	-2.20E-05	1.62	1.36E-05	-1.56E-05	5.95E-05	0.1152	-0.31
16.04	131	19	-2.41E-05	1.79	1.34E-05	-1.31E-05	6.13E-05	0.0828	-0.25
16.68	132	18	-2.46E-05	1.92	1.28E-05	-1.08E-05	6.01E-05	0.0645	-0.34
17.32	134	18	-2.65E-05	2.04	1.30E-05	-9.41E-06	6.23E-05	0.0502	-0.40
17.96	132	20	-2.84E-05	2.30	1.23E-05	-5.75E-06	6.25E-05	0.0275	-0.36
18.60	131	22	-3.45E-05	2.48	1.39E-05	-4.08E-06	7.30E-05	0.0189	-0.31
19.24	126	23	-3.53E-05	2.65	1.33E-05	-1.56E-06	7.22E-05	0.0119	-0.39
19.88	123	24	-3.78E-05	2.88	1.31E-05	1.43E-06	7.41E-05	0.0063	-0.45
20.52	112	25	-3.99E-05	3.10	1.29E-05	4.25E-06	7.56E-05	0.0033	-0.48
21.16	106	28	-4.01E-05	3.08	1.30E-05	4.06E-06	7.61E-05	0.0033	-0.50
21.81	106	27	-4.13E-05	3.07	1.34E-05	4.07E-06	7.85E-05	0.0035	-0.43
22.45	96	27	-3.96E-05	3.01	1.31E-05	3.21E-06	7.60E-05	0.0043	-0.43
23.09	82	25	-3.69E-05	2.60	1.42E-05	-2.35E-06	7.62E-05	0.0136	-0.40
23.73	74	25	-3.55E-05	2.35	1.51E-05	-6.24E-06	7.72E-05	0.0236	-0.41
24.37	62	24	-3.76E-05	2.47	1.52E-05	-4.46E-06	7.96E-05	0.0175	-0.42
25.01	56	24	-4.09E-05	2.63	1.56E-05	-2.20E-06	8.39E-05	0.0120	-0.44
25.65	49	22	-4.17E-05	2.31	1.81E-05	-8.38E-06	9.18E-05	0.0265	-0.43
26.29	38	21	-3.72E-05	2.22	1.68E-05	-9.24E-06	8.36E-05	0.0336	-0.39
26.93	35	21	-4.14E-05	2.06	2.01E-05	-1.42E-05	9.69E-05	0.0482	-0.39
27.58	26	20	-4.22E-05	1.79	2.36E-05	-2.31E-05	1.08E-04	0.0853	-0.44
28.22	21	20	-3.58E-05	1.52	2.35E-05	-2.93E-05	1.01E-04	0.1416	-0.36

Supplementary Table S17. Modulating effects of deletion type. Cohen's d, Student's t and p-values for the effects of deletion type on each ROI-by-DTI measure. The model was tested on all samples (Utrecht included) using the harmonized data as a mega-analysis. There was no p-value controlling the FDR at a q-value of 0.05. Orange-shadowed cells correspond to uncorrected significant results ($p < 0.05$). The model included age, $[\text{age-mean(age)}]^2$ and sex as covariates.

JHU-ROI	FA		
	Deletion_type		
	d	t	p
ACR	-0.139	-1.010	0.312
ALIC	0.054	0.390	0.695
Average WM	0.145	1.056	0.292
BCC	0.079	0.580	0.566
CGC	0.180	1.310	0.192
EC	-0.114	-0.830	0.407
FXST	0.081	0.590	0.556
GCC	0.023	0.170	0.868
UNC	-0.035	-0.260	0.797
PCR	0.139	1.010	0.313
PLIC	0.203	1.480	0.140
PTR	-0.065	-0.470	0.638
RLIC	0.186	1.350	0.178
SCC	0.064	0.460	0.643
SCR	0.157	1.150	0.253
SFO	-0.090	-0.650	0.514
SLF	0.040	0.290	0.770
SS	-0.007	-0.050	0.958
TAP	0.278	2.020	0.044

JHU-ROI	MD		
	Deletion_type		

	d	t	p
**ACR**	-0.154	-1.120	0.264
**ALIC**	-0.006	-0.040	0.968
**Average WM**	0.130	0.947	0.345
**BCC**	0.032	0.230	0.817
**CGC**	-0.037	-0.270	0.790
**EC**	-0.069	-0.500	0.615
**FXST**	-0.078	-0.570	0.571
**GCC**	-0.126	-0.910	0.362
**UNC**	0.042	0.310	0.760
**PCR**	-0.232	-1.690	0.093
**PLIC**	-0.043	-0.320	0.753
**PTR**	-0.083	-0.600	0.548
**RLIC**	-0.111	-0.810	0.420
**SCC**	0.049	0.360	0.720
**SCR**	-0.095	-0.690	0.492
**SFO**	-0.042	-0.300	0.761
**SLF**	0.020	0.150	0.883
**SS**	-0.029	-0.210	0.831
**TAP**	-0.190	-1.380	0.169

**JHU-ROI**	**AD**		
	**Deletion_type**		
	**d**	**t**	**p**
**ACR**	-0.336	-2.440	0.015
**ALIC**	-0.119	-0.860	0.388
**Average WM**	0.136	0.988	0.324
**BCC**	-0.053	-0.380	0.702
**CGC**	0.027	0.190	0.846
**EC**	-0.300	-2.180	0.030

**FXST**	-0.088	-0.640	0.523
**GCC**	-0.265	-1.930	0.055
**UNC**	-0.114	-0.830	0.407
**PCR**	-0.236	-1.720	0.087
**PLIC**	-0.070	-0.510	0.610
**PTR**	-0.137	-1.000	0.318
**RLIC**	-0.073	-0.530	0.596
**SCC**	-0.048	-0.350	0.726
**SCR**	-0.003	-0.020	0.984
**SFO**	-0.154	-1.120	0.263
**SLF**	0.012	0.090	0.930
**SS**	-0.079	-0.570	0.568
**TAP**	0.049	0.350	0.724

**JHU-ROI**	**RD**		
	**Deletion_type**		
	**d**	**t**	**p**
**ACR**	-0.022	-0.160	0.874
**ALIC**	0.021	0.160	0.877
**Average WM**	0.012	0.087	0.931
**BCC**	-0.032	-0.230	0.817
**CGC**	-0.118	-0.860	0.391
**EC**	0.080	0.580	0.561
**FXST**	-0.079	-0.580	0.564
**GCC**	-0.012	-0.080	0.933
**UNC**	0.070	0.510	0.611
**PCR**	-0.197	-1.430	0.154
**PLIC**	-0.150	-1.090	0.275
**PTR**	-0.056	-0.410	0.682
**RLIC**	-0.183	-1.330	0.184

**SCC**	-0.008	-0.060	0.952
**SCR**	-0.163	-1.190	0.236
**SFO**	0.041	0.300	0.764
**SLF**	-0.033	-0.240	0.811
**SS**	-0.018	-0.130	0.898
**TAP**	-0.267	-1.950	0.053

Supplementary Table S18. Correlation between DTI measures and IQ for each ROI. Partial correlations, Student's *t* and p-values for the effects of IQ on each ROI-by-DTI measure for 22q11DS and healthy controls. Regressions included age, $[age-mean(age)]^2$ and sex as covariates. Blue-shadowed cells indicate a statistically significant result that passed the False Discovery Rate threshold at a q-value of 0.05. Orange-shadowed cells correspond to uncorrected significant results ($p < 0.05$).

**JHU-ROI**	**FA**					
	**IQ - 22q11.2DS**			**IQ - Healthy Controls**		
	**r**	**t**	**p** (FDR p < 0.000014)	**r**	**t**	**p**
**ACR**	-0.059	-0.980	0.327	0.071	0.720	0.475
**ALIC**	0.053	0.890	0.376	-0.060	-0.610	0.544
**Average WM**	0.036	0.600	0.549	-0.110	-1.120	0.265
**BCC**	-0.005	-0.090	0.926	-0.010	-0.100	0.920
**CGC**	-0.031	-0.520	0.602	-0.047	-0.480	0.632
**EC**	0.087	1.460	0.145	0.091	0.920	0.360
**FXST**	0.080	1.330	0.186	0.020	0.200	0.842
**GCC**	-0.051	-0.850	0.396	-0.037	-0.370	0.710
**UNC**	0.007	0.120	0.908	0.124	1.260	0.208
**PCR**	-0.057	-0.950	0.341	-0.122	-1.240	0.216
**PLIC**	-0.025	-0.410	0.684	-0.118	-1.200	0.232
**PTR**	0.142	2.390	0.017	-0.095	-0.960	0.338
**RLIC**	0.023	0.380	0.702	-0.060	-0.610	0.545
**SCC**	-0.017	-0.280	0.782	-0.100	-1.020	0.311
**SCR**	-0.063	-1.050	0.293	-0.092	-0.930	0.352

**SFO**	0.012	0.200	0.838	0.009	0.090	0.928
**SLF**	0.089	1.480	0.141	0.043	0.430	0.671
**SS**	0.061	1.020	0.309	-0.004	-0.040	0.965
**TAP**	-0.077	-1.290	0.198	-0.256	-2.680	0.008

**JHU-ROI**	**MD**					
	**IQ - 22q11.2DS**			**IQ - Healthy Controls**		
	**r**	**t**	**p**	**r**	**t**	**p**
**ACR**	0.067	1.120	0.263	0.170	1.740	0.083
**ALIC**	-0.018	-0.300	0.763	0.221	2.290	0.023
**Average WM**	0.109	1.830	0.068	-0.017	-0.170	0.864
**BCC**	-0.049	-0.820	0.415	0.100	1.020	0.309
**CGC**	0.040	0.660	0.508	0.070	0.710	0.480
**EC**	0.005	0.090	0.927	0.126	1.280	0.203
**FXST**	0.035	0.580	0.561	0.058	0.590	0.559
**GCC**	0.116	1.940	0.049	0.172	1.760	0.080
**UNC**	-0.068	-1.130	0.258	0.006	0.060	0.953
**PCR**	-0.001	-0.010	0.988	0.250	2.610	0.010
**PLIC**	-0.034	-0.570	0.572	0.062	0.630	0.527
**PTR**	0.102	1.710	0.088	0.232	2.410	0.017
**RLIC**	-0.054	-0.900	0.370	0.189	1.940	0.054
**SCC**	-0.011	-0.180	0.854	0.199	2.050	0.042
**SCR**	-0.033	-0.550	0.583	0.173	1.770	0.079
**SFO**	0.034	0.560	0.573	0.268	2.810	0.006
**SLF**	-0.061	-1.010	0.316	0.209	2.160	0.032
**SS**	0.052	0.860	0.390	0.204	2.100	0.037
**TAP**	-0.019	-0.320	0.751	0.243	2.530	0.012

**JHU-ROI**	**AD**					
	**IQ - 22q11.2DS**			**IQ - Healthy Controls**		
	**r**	**t**	**p**	**r**	**t**	**p**

**ACR**	0.064	1.060	0.292	0.220	2.280	0.024
**ALIC**	0.075	1.250	0.211	0.203	2.090	0.039
**Average WM**	0.125	2.100	0.037	-0.100	-1.010	0.316
**BCC**	-0.003	-0.050	0.963	0.089	0.900	0.372
**CGC**	0.026	0.440	0.657	0.037	0.370	0.714
**EC**	0.090	1.500	0.135	0.270	2.830	0.005
**FXST**	0.112	1.870	0.062	0.057	0.580	0.560
**GCC**	0.142	2.380	0.018	0.175	1.790	0.075
**UNC**	-0.018	-0.300	0.766	0.156	1.590	0.114
**PCR**	0.000	0.000	0.999	0.256	2.670	0.008
**PLIC**	-0.004	-0.070	0.944	-0.009	-0.090	0.926
**PTR**	0.257	4.420	1.37E-05	0.132	1.350	0.177
**RLIC**	0.011	0.180	0.856	0.243	2.530	0.012
**SCC**	0.009	0.150	0.884	0.159	1.630	0.105
**SCR**	-0.025	-0.410	0.681	0.073	0.740	0.458
**SFO**	0.052	0.860	0.391	0.247	2.570	0.011
**SLF**	0.072	1.200	0.230	0.205	2.110	0.036
**SS**	0.162	2.730	0.006	0.287	3.030	0.003
**TAP**	-0.064	-1.070	0.288	-0.135	-1.380	0.170

**JHU-ROI**	**RD**					
	**IQ - 22q11.2DS**			**IQ - Healthy Controls**		
	**r**	**t**	**p**	**r**	**t**	**p**
**ACR**	0.076	1.270	0.205	0.093	0.940	0.347
**ALIC**	-0.064	-1.060	0.291	0.132	1.340	0.181
**Average WM**	-0.031	-0.510	0.611	0.176	1.810	0.072
**BCC**	-0.038	-0.630	0.530	0.023	0.230	0.818
**CGC**	0.024	0.400	0.688	0.048	0.490	0.622
**EC**	-0.043	-0.710	0.480	-0.035	-0.350	0.725
**FXST**	-0.031	-0.520	0.604	0.034	0.340	0.736

**GCC**	0.078	1.300	0.195	0.089	0.900	0.368
**UNC**	-0.048	-0.800	0.426	-0.096	-0.970	0.332
**PCR**	-0.004	-0.060	0.954	0.185	1.900	0.060
**PLIC**	-0.021	-0.350	0.728	0.054	0.550	0.585
**PTR**	-0.061	-1.010	0.312	0.210	2.170	0.031
**RLIC**	-0.062	-1.030	0.304	0.126	1.280	0.203
**SCC**	0.001	0.010	0.992	0.149	1.520	0.129
**SCR**	0.002	0.040	0.971	0.171	1.750	0.082
**SFO**	0.021	0.350	0.724	0.175	1.800	0.074
**SLF**	-0.106	-1.770	0.079	0.091	0.920	0.359
**SS**	-0.032	-0.530	0.598	0.106	1.080	0.284
**TAP**	0.022	0.360	0.723	0.314	3.340	0.001

Supplementary tables references

[1] Structured Clinical Interview for DSM-IV-TR Axis I Disorders, Research Version, Patient Edition. (SCID-I/P).
First MB, Spitzer RL, Gibbon M, Williams JBW. New York: Biometrics Research, New York State Psychiatric Institute; 2002.

[2] Diagnostic Interview Schedule for Children. Shaffer D, Schwab-Stone M, Fisher P, Cohen P, Piacentini J, Davies M et al. The Diagnostic Interview Schedule for Children–Revised Version (DISC-R), I:
preparation, field testing, interrater reliability, and acceptability. J *Am Acad Child Adolesc Psychiatry* 1993; **32**:643–650.

[3] Structured Interview for Prodromal Syndromes.
McGlashan TH. *Structured Interview for Prodromal Syndromes (SIPS)*. Yale University: New Haven, 2001.

[4] Brief Psychiatric Rating Scale.
Ventura J, Green MF, Shaner A, Liberman RP. Training and quality assurance with the Brief Psychiatric Rating Scale: The drift busters. Int J Methods
Psychiatr Res 1993;**3**: 221–244.

[5] Kiddie Schedule for Affective Disorders and Schizophrenia- Present and Lifetime Version.
Kaufman J, Birmaher B, Brent D, Rao U, Flynn C, Moreci P et al. Schedule for affective disorders and schizophrenia for school-age children-present and lifetime version (K-SADS-PL): initial reliability and validity data. *J Am Acad Child Adolesc Psychiatry* 1997; **36**:980–988.

[6] Positive and Negative Syndrome Scale.
Kay SR, Fiszbein A, Opler LA. The positive and negative syndrome scale (PANSS) for schizophrenia. *Schizophr Bull* 1987;**13**:261–276.

[7] Mini International Neuropsychiatric Interview.
Sheehan DV, Lecrubier Y, Harnett-Sheehan K, Janavs J, Weiller E, Keskiner A et al. The validity of the Mini International Neuropsychiatric Interview (MINI)
according to the SCID-P and its reliability. *Eur Psychiatry* 1997; **12**:232–241.

[8] Psychiatric Assessment Schedules for Adults with Developmental Disabilities.
Moss S, Patel P, Prosser H, Goldberg D, Simpson N, Rowe S et al. Psychiatric morbidity in older people with moderate and severe learning disability. I: Development and reliability of the patient interview (PAS-ADD). *Br J Psychiatry* 1993; **163**:471–480.

[9] Child and Adolescent Psychiatric Assessment (CAPA) version 4.2.
Angold A, Prendergast M, Cox A, Harrington R, Simonoff E, Rutter M. The Child and Adolescent Psychiatric Assessment (CAPA). *Psychol Med* 1995; **25**:739–
753.

[10] Structured Clinical Interview for DSM_IV Axis II Personality Disorders. First
MB, Spitzer RL, Gibbon M, Williams JBW. *Structured Clinical Interview
for DSM-IV Axis I Disorders: Patient Edition Biometrics Research.* Biometrics Research, New York State Psychiatric Institute: New York, 2002.

[11] Scale for the Assessment of Positive Symptoms.
Andreasen NC*. Scale for the assessment of positive symptoms.* University of Iowa; Iowa City, 1984.

[12] Scale for the Assessment of Negative Symptoms.
Andreasen NC. *Scale for the assessment of negative symptoms.* University of Iowa; Iowa City, 1984.

[13] Schizophrenia Proneness Instrument-Adult.
Schultze-Lutter F, Addington J, Ruhrmann S, Klosterkötter J. *Schizophrenia Proneness Instrument (SPI-A).* Giovanni Fioriti: Rome, Italy, 2007.

[14] Ho JS, Radoeva PD, Jalbrzikowski M, Chow C, Hopkins J, Tran W et al. Deficits in mental state attributions in individuals with 22q11.2 deletion syndrome
(velo-cardio-facial syndrome). *Autism* 2012; **5**: 407-418.

[15] Jalbrzikowski M, Carter C, Senturk D, Chow C, Hopkins JM, Green MF et al. Social cognition in 22q11.2 microdeletion syndrome: Relevance to psychosis. *Schizophr Res* 2012; **142**(0)**:** 99-107.

[16] Jalbrzikowski M, Jonas R, Senturk D, Patel A, Chow C, Green MF et al. Structural abnormalities in cortical volume, thickness, and surface area in 22q11.2 microdeletion syndrome: Relationship with psychotic symptoms. *NeuroImage: Clin* 2013; **3:** 405–415.

[17] Jalbrzikowski M, Villalon-Reina JE, Karlsgodt KH, Senturk D, Chow C, Thompson PM et al. Altered white matter microstructure is associated with social cognition and psychotic symptoms in 22q11.2 microdeletion syndrome. *Front Behav Neurosci* 2014; **8:** 393.

[18] Jonas RK, Jalbrzikowski M, Montojo CA, Patel A, Kushan L, Chow CC et al. Altered brain structure-function relationships underlie executive dysfunction in
22q11.2 deletion syndrome. *Mol Neuropsychiatry* 2015; **1**: 235–246.

[19] Schreiner MJ, Lazaro MT, Jalbrzikowski M, Bearden CE. Converging levels of analysis on a genomic hotspot for psychosis: Insights from 22q11.2 Deletion Syndrome. *Neuropharmacology* 2013; **68:** 157-173.

[20] Schreiner M, Forsyth JK, Karlsgodt KH, Anderson AE, Hirsh N, Kushan L et al. Intrinsic connectivity network-based classification and detection of psychotic symptoms in youth with 22q11.2 deletions. *Cereb Cortex* 2017; **27:** 3294-3306.

[21] Radoeva PD, Coman IL, Salazar CA, Gentile KL, Higgins AM, Middleton FA et al. Association between autism spectrum disorder in individuals with velocardiofacial (22q11.2 deletion) syndrome and PRODH and COMT genotypes. *Psychiatr Genet* 2014; **24:**269-272.

[22] Antshel KM, Hendricks K, Shprintzen R, Fremont W, Higgins AM, Faraone SV et al. The longitudinal course of attention deficit/hyperactivity disorder in velo-cardio-facial syndrome. *J Pediatr* 2013;**163**:187-193.

[23] Radoeva PD, Coman IL, Antshel KM, Fremont W, McCarthy CS, Kotkar A et al. Atlas-based white matter analysis in individuals with velo-cardio-facial syndrome (22q11.2 deletion syndrome) and unaffected siblings. *Behav Brain Funct* 2012;**8**:38.

[24] Kunwar A, Ramanathan S, Nelson J, Antshel KM, Fremont W, Higgins AM, et al. Cortical gyrification in velo-cardio-facial (22q11.2 deletion) syndrome: a longitudinal study. *Schizophr Res* 2012;**137**:20-25.

[25] Kates WR, Antshel KM, Faraone SV, Fremont WP, Higgins AM, Shprintzen RJ et al. Neuroanatomic predictors to prodromal psychosis in velocardiofacial syndrome (22q11.2 deletion syndrome): a longitudinal study. *Biol Psychiatry* 2011;**69**:945-952.

[26] Kates WR, Bansal R, Fremont W, Antshel KM, Hao X, Higgins AM et al. Mapping cortical morphology in youth with velocardiofacial (22q11.2 deletion) syndrome. *J Am Acad Child Adolesc Psychiatry* 2011;**50**:272-282.

[27] Coman IL, Gnirke MH, Middleton FA, Antshel KM, Fremont W, Higgins AM et al. The effects of gender and catechol O-methyltransferase (COMT) Val108/158Met polymorphism on emotion regulation in velo-cardio-facial syndrome (22q11.2 deletion syndrome): An fMRI study. *Neuroimage* 2010;**53**:1043-1050.

[28] Roizen NJ, Higgins AM, Antshel KM, Fremont W, Shprintzen R, Kates WR. 22q11.2 deletion syndrome: are motor deficits more than expected for IQ level? *J Pediatr* 2010;**157**:658-661.

[29] Antshel KM, Shprintzen R, Fremont W, Higgins AM, Faraone SV, Kates WR. Cognitive and psychiatric predictors to psychosis in velocardiofacial syndrome: a3-year follow-up study. *J Am Acad Child Adolesc Psychiatry* 2010;**49**:333-344.

[30] Antshel KM, Peebles J, AbdulSabur N, Higgins AM, Roizen N, Shprintzen R et al. Associations between performance on the Rey-Osterrieth Complex Figure and regional brain volumes in children with and without velocardiofacial syndrome. *Dev Neuropsychol* 2008;**33**:601-622.

[31] Kates WR, Antshel KM, Fremont WP, Shprintzen RJ, Strunge LA, Burnette CP et al. Comparing phenotypes in patients with idiopathic autism to patients with velocardiofacial syndrome (22q11 DS) with and without autism. *Am J Med Genet A* 2007;**143A**:2642-2650.

[32] Antshel KM, Aneja A, Strunge L, Peebles J, Fremont WP, Stallone K et al. Autistic spectrum disorders in velo-cardio facial syndrome (22q11.2 deletion). *J Autism Dev Disord* 2007;**37**:1776-1786..

[33] Kates WR, Krauss BR, Abdulsabur N, Colgan D, Antshel KM, Higgins AM et al. The neural correlates of non-spatial working memory in velocardiofacial syndrome (22q11.2 deletion syndrome). *Neuropsychologia* 2007;**45**:2863-2873.

[34] Antshel KM, Stallone K, Abdulsabur N, Shprintzen R, Roizen N, Higgins AM et al. Temperament in velocardiofacial syndrome. *J Intellect Disabil Res* 2007;**51**:218-227.

[35] Aneja A, Fremont WP, Antshel KM, Faraone SV, AbdulSabur N, Higgins AM et al. Manic symptoms and behavioral dysregulation in youth with velocardiofacial syndrome (22q11.2 deletion syndrome). *J Child Adolesc Psychopharmacol* 2007;**17**:105-114.

[36] Antshel KM, Fremont W, Roizen NJ, Shprintzen R, Higgins AM, Dhamoon A et al. ADHD, major depressive disorder, and simple phobias are prevalent psychiatric conditions in youth with velocardiofacial syndrome. *J Am Acad Child Adolesc Psychiatry* 2006;**45**:596-603.

[37] Kates WR, Antshel KM, Abdulsabur N, Colgan D, Funke B, Fremont W et al. A gender-moderated effect of a functional COMT polymorphism on prefrontal brain morphology and function in velo-cardio-facial syndrome (22q11.2 deletion syndrome). *Am J Med Genet B Neuropsychiatr Genet* 2006;**141B**:274-280.

[38] Scott JA, Goodrich-Hunsaker N, Kalish K, Lee A, Hunsaker MR, Schumann CM et al. The hippocampi of children with chromosome 22q11.2 deletion syndrome have localized anterior alterations that predict severity of anxiety. *J Psychiatry Neurosci* 2016;**41**:203-213.

[39] Deng Y, Goodrich-Hunsaker NJ, Cabaral M, Amaral DG, Buonocore MH, Harvey D et al. Disrupted fornix integrity in children with chromosome 22q11.2 deletion syndrome. *Psychiatry Res* 2015;**232**:106-114.

[40] Stephenson DD, Beaton EA, Weems CF, Angkustsiri K, Simon TJ. Identifying patterns of anxiety and depression in children with chromosome 22q11.2 deletion syndrome: comorbidity predicts behavioral difficulties and impaired functional communications. *Behav Brain Res* 2015;**276**:190-198.

[41] Yin JJ, Tang SX, McDonald-McGinn DM, Calkins ME, Whinna DA, Souders MC *et al*. Contribution of congenital heart disease to neuropsychiatric outcome in school-age children with 22q11.2 deletion syndrome. *Am J Med Genet B Neuropsychiatr Genet* 2014; **0**:137–147.

[42] Niarchou M, Moore TM, Tang SX, Calkins ME, McDonald-McGuinn DM, Zackai EH et al.The dimensional structure of psychopathology in 22q11.2 Deletion Syndrome. *J Psychiatr Res* 2017;**92**:124-131.

[43] Tang SX, Moore TM, Calkins ME, Yi JJ, McDonald-McGinn DM, Zackai EH et al. Emergent, remitted and persistent psychosis-spectrum symptoms in 22q11.2 deletion syndrome. *Transl Psychiatry* 2017;**7**:e1180.

[44] Tang SX, Moore TM, Calkins ME, Yi JJ, Savitt A, Kohler CG et al. The Psychosis Spectrum in 22q11.2 Deletion Syndrome Is Comparable to That of Nondeleted Youths. *Biol Psychiatry* 2017;**82**:17-25.

[45] Fiksinski AM, Breetvelt EJ, Duijff SN, Bassett AS, Kahn AS, Vorstman JA. Autism spectrum and psychosis risk in the 22q11.2 deletion syndrome. Findings from a prospective longitudinal study. *Schizophr Res* 2017; e-pub ahead of print 21 January 2017; doi: 10.1016/j.schres.2017.01.032.

[46] Bakker G, Caan MWA, Vingerhoets WAM, da Silva-Alves F, de Koning M, Boot E, *et al.* Cortical morphology differences in subjects at increased vulnerability for developing a psychotic disorder: A comparison between subjects with ultra-high risk and 22q11.2 deletion syndrome. *PLoS ONE* 2016; **11:** e0159928.

[47] Da Silva-Alves F, Schmitz N, Bloemen O, can der Meer J, Meijer J, Boot E et al. White matter abnormalities in adults with 22q11 deletion syndrome with and without schizophrenia. *Schizophr Res* 2011;**132**: 75-83.

[48] Monks S, Niarchou M, Davies AR, Walters JT, Williams N, Owen MJ *et al.* Further evidence for high rates of schizophrenia in 22q11.2 deletion syndrome.
Schizophr Res 2014;**153**: 231-236.

[49] Niarchou M, Zammit S, van Goozen SHM, Thapar A, Tierling HM, Owen MJ et al. Psychopathology and cognition in children with 22q11.2 deletion syndrome. *Br J Psychiatry* 2014; **204**: 46–54.

[50] Niarchou M, Martin J, Thapar A, Owen MJ, van den Bree MBM. The clinical presentation of attention deficit-hyperactivity disorder (ADHD) in children with
22q11.2 deletion syndrome. *Am J Med Genet* 2015; **168**: 730–738.

[51] Chawner S, Doherty JL, Moss H, Niarchou M, Walters J, Owen MJ et al. Case-control study finds no evidence that 22q11.2 Deletion Syndrome is associated with cognitive deterioration. *Br J Psychiatry* (in press)

[52] Campbell LE, Azuma R, Ambery F, Stevens A, Smith A, Morris RG et al. Executive functions and memory abilities in children with 22q11.2 deletion syndrome. *Aust N Z J Psychiatry* 2010; **44:** 364-371.

[53] Campbell LE, McCabe KL, Melville JL, Strutt PA, Schall U. Social cognition dysfunction in adolescents with 22q11.2 deletion syndrome (velo-cardio-facial syndrome): relationship with executive functioning and social competence/functioning*. J Intellect Disabil Res* 2015; **59:** 845-859.

[54] McCabe KL & Carr VJ. Relationship between childhood adversity and clinical and cognitive features in schizophrenia. *J Psychiatr Res* 2012; **46**: 600-607.

[55] McCabe KL, Melville JL, Rich D, Strutt PA, Cooper G, Loughland CM et al*.* Divergent patterns of social cognition performance in autism and 22q11.2 deletion syndrome (22q11DS). *J Autism Dev Disord* 2013; **43**: 1926-1934.

[56] McCabe KL, Atkinson RJ, Cooper G, Melville JL, Harris J, Schall U et al. Pre-pulse inhibition and antisaccade performance indicate impaired attention modulation of cognitive inhibition in 22q11.2 deletion syndrome. *J Neurodev Disord* 2014; **6**:38.

[57] Wechsler Abbreviated Scale of Intelligence
Wechsler D. Wechsler abbreviated scale of intelligence. Harcourt Brace & Company. New York, NY: The Psychological Corporation; 1999.

[58] Wechsler Intelligence Scale for Children – Third Edition
Wechsler D. *Wechsler Intelligence Scale for Children.*3rd ed. San Antonio, TX: Psychological Corp; 1991.

[59] Wechsler Intelligence Scale for Children –Fourth Edition
Wechsler D. *Wechsler Intelligence Scale for Children.* 4th ed. San Antonio, TX: Psychological Corp; 2003.

[60] Wechsler Adult Intelligence Scale – Third Edition
Wechsler D. Wechsler adult intelligence scale - 3rd ed. San Antonio,TX: Psychological Corp; 1997.

[61] Wechsler Intelligence Scale for Children—Revised
Wechsler D. Manual of the Wechsler intelligence scale for children-revised. New York: Psychological Corp; 1974.
