# Supplementary material for: Altered white matter microstructure in 22q11.2 deletion syndrome: a multisite diffusion tensor imaging study
Source: Mol Psychiatry. 2019 Jul 29;25(11):2818–31. doi: 10.1038/s41380-019-0450-0 (PMC6986984; doi:10.1038/s41380-019-0450-0)

Supplementary Figures

Meta-analysis results. Results of the meta-analysis, including nine independent datasets from the ENIGMA-22q11DS working group. Effect sizes for each dataset are shown separately as colored dots. The model tested was:

DTI-ROI-measure$=\beta_0 + \beta_1 Diagnosis + \beta_2 Sex + \beta_3 Age + \beta_4 Age^2_{centered.}$

WM = Average of all white matter JHU-ICBM ROIs.

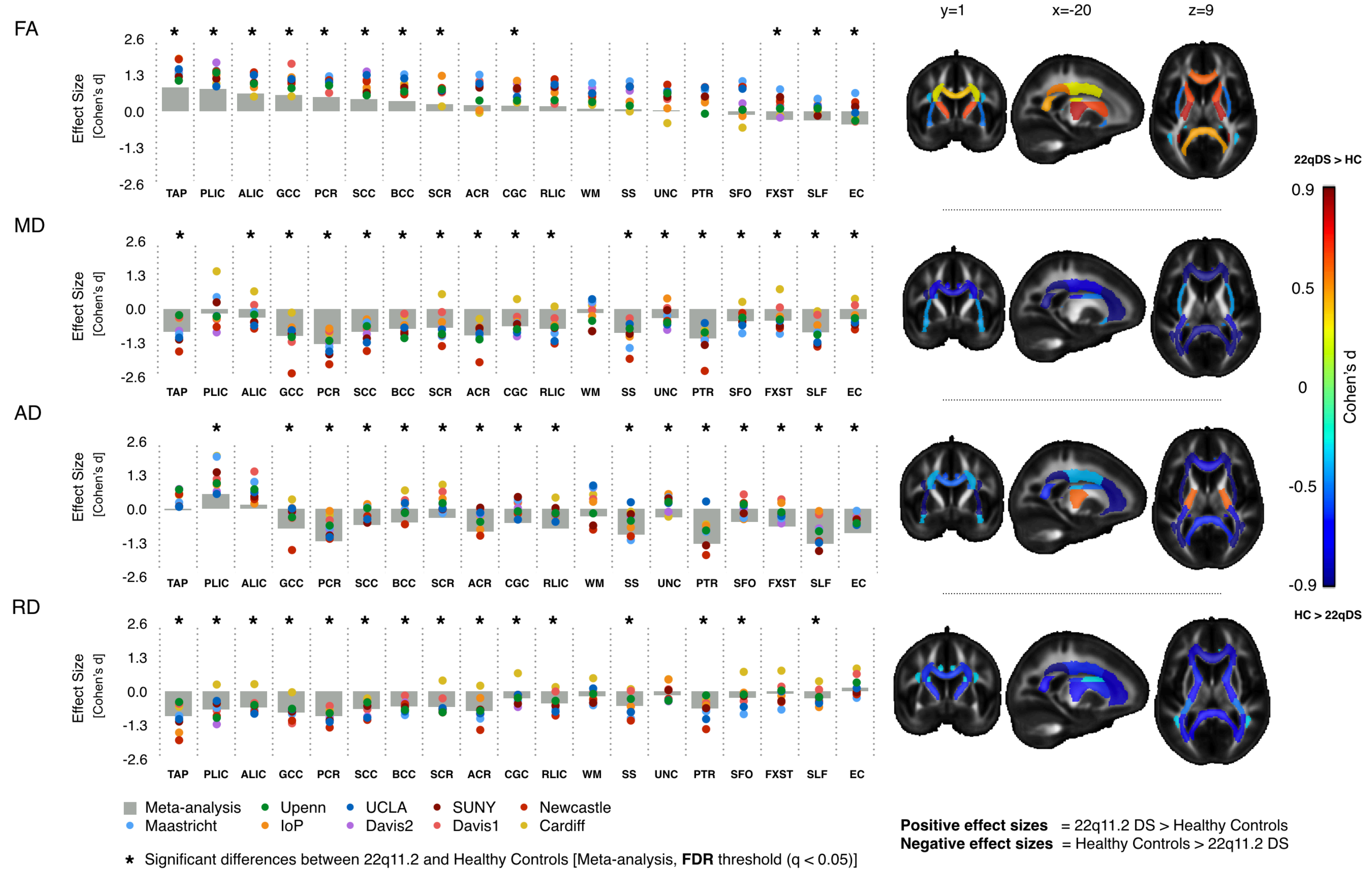

Supplementary Figure 2. Nonlinear fits of age for Fractional Anisotropy (FA). Nonlinear Poisson fits of age for FA indices, for typically developing healthy controls (HC; blue) and 22q11.2 Deletion Syndrome (22q11DS; red).

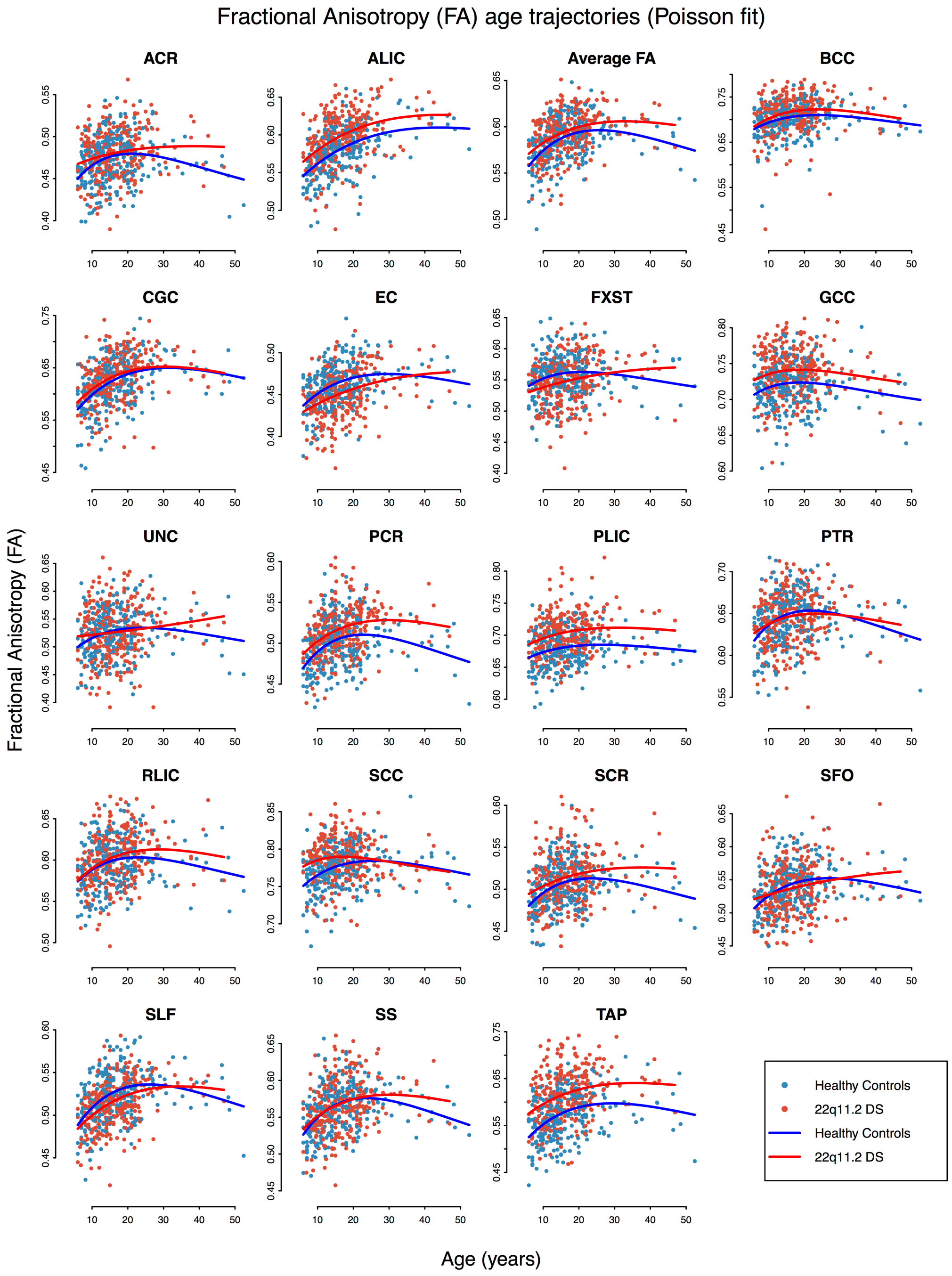

Supplementary Figure 3. Nonlinear fits of age for Mean Diffusivity (MD). Nonlinear Poisson fits of age for MD indices for HC (blue) and (22q11DS (red).

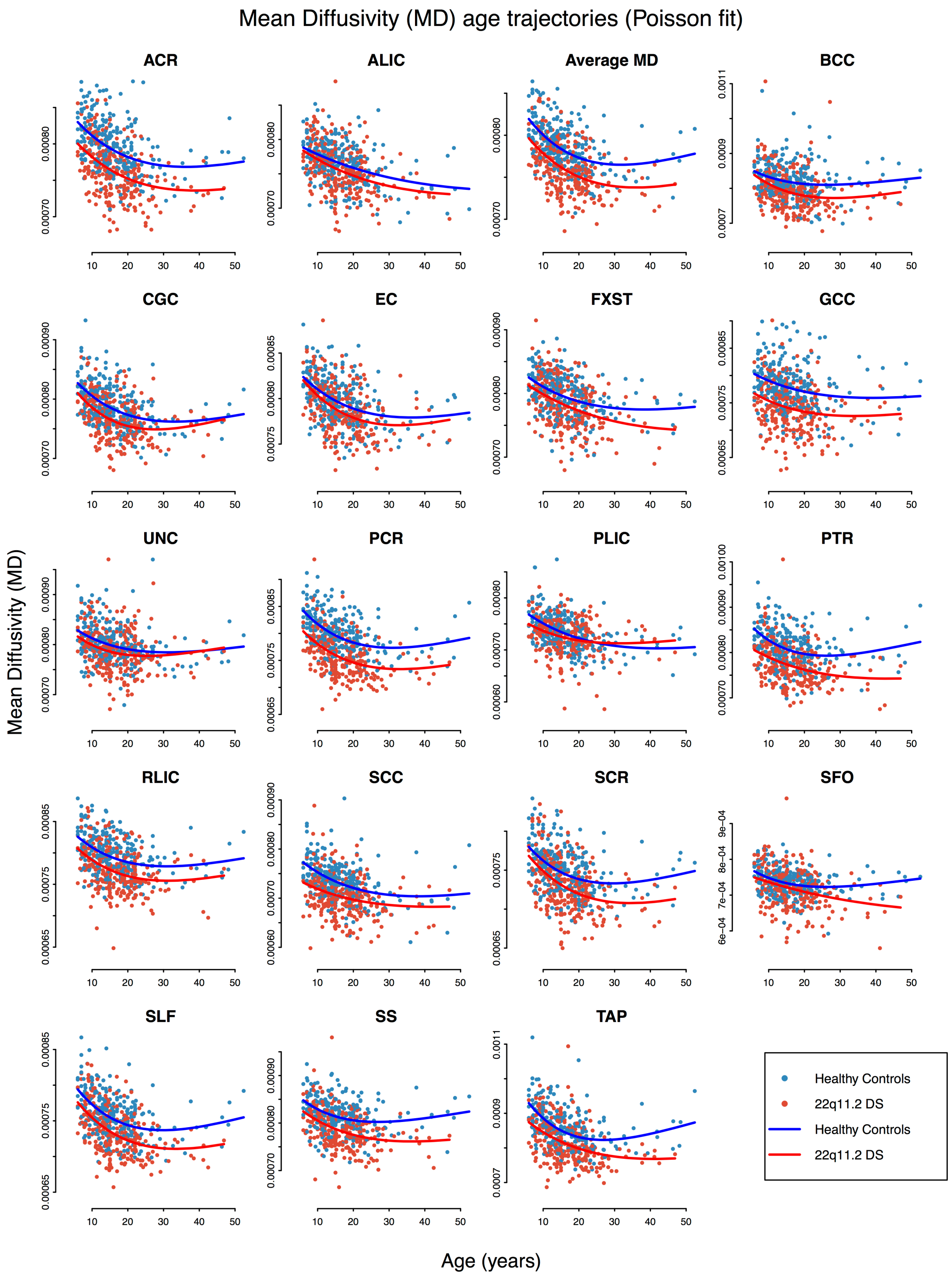

Supplementary Figure 4. Nonlinear fits of age for Radial Diffusivity (RD). Nonlinear Poisson fits of age for AD for HC(blue) and 22q11DS (red).

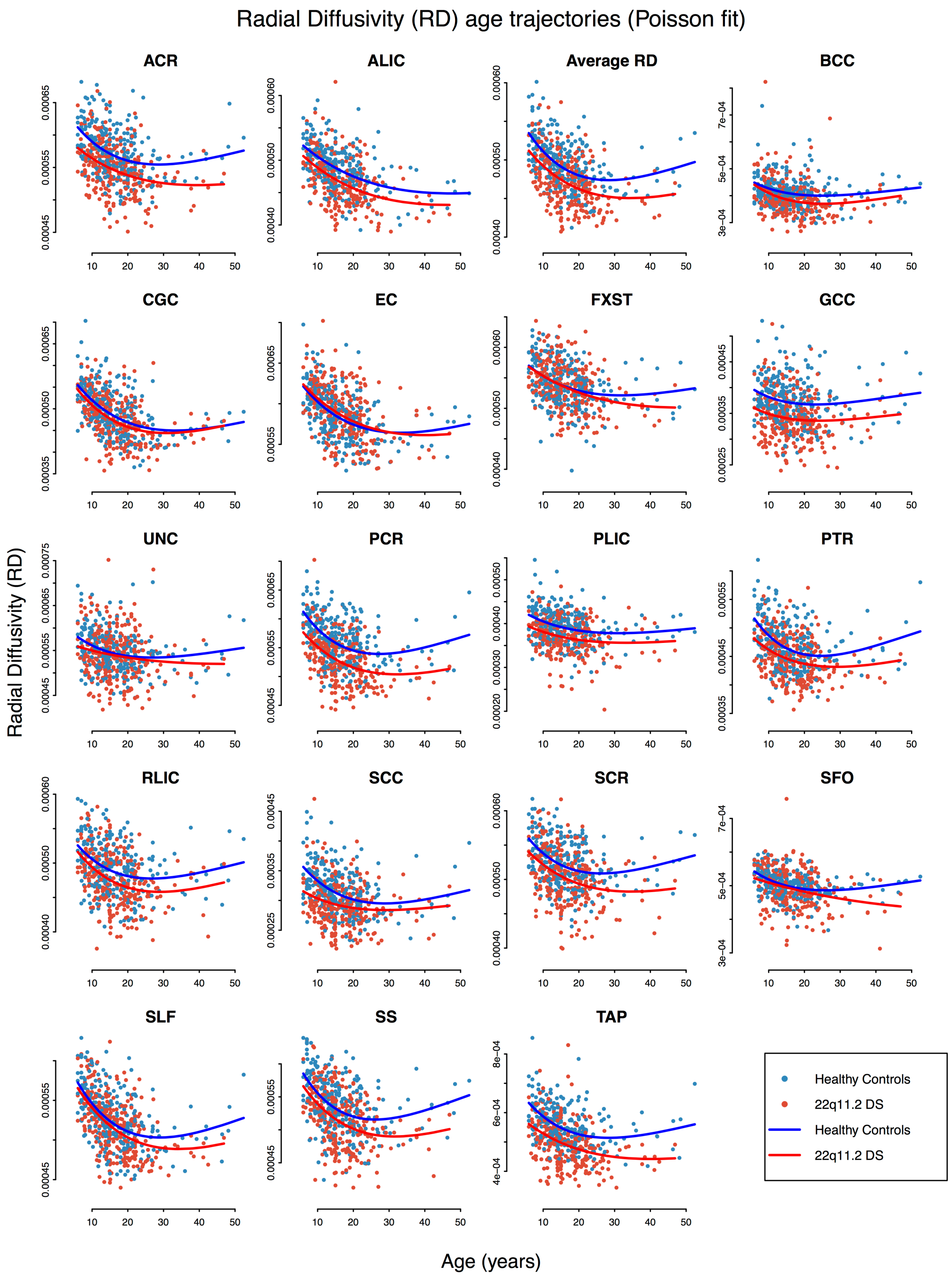

Supplementary Figure 5. Nonlinear fits of age for Axial Diffusivity (AD). Nonlinear Poisson fits of age for RD for HC (blue) and 22q11DS (red).

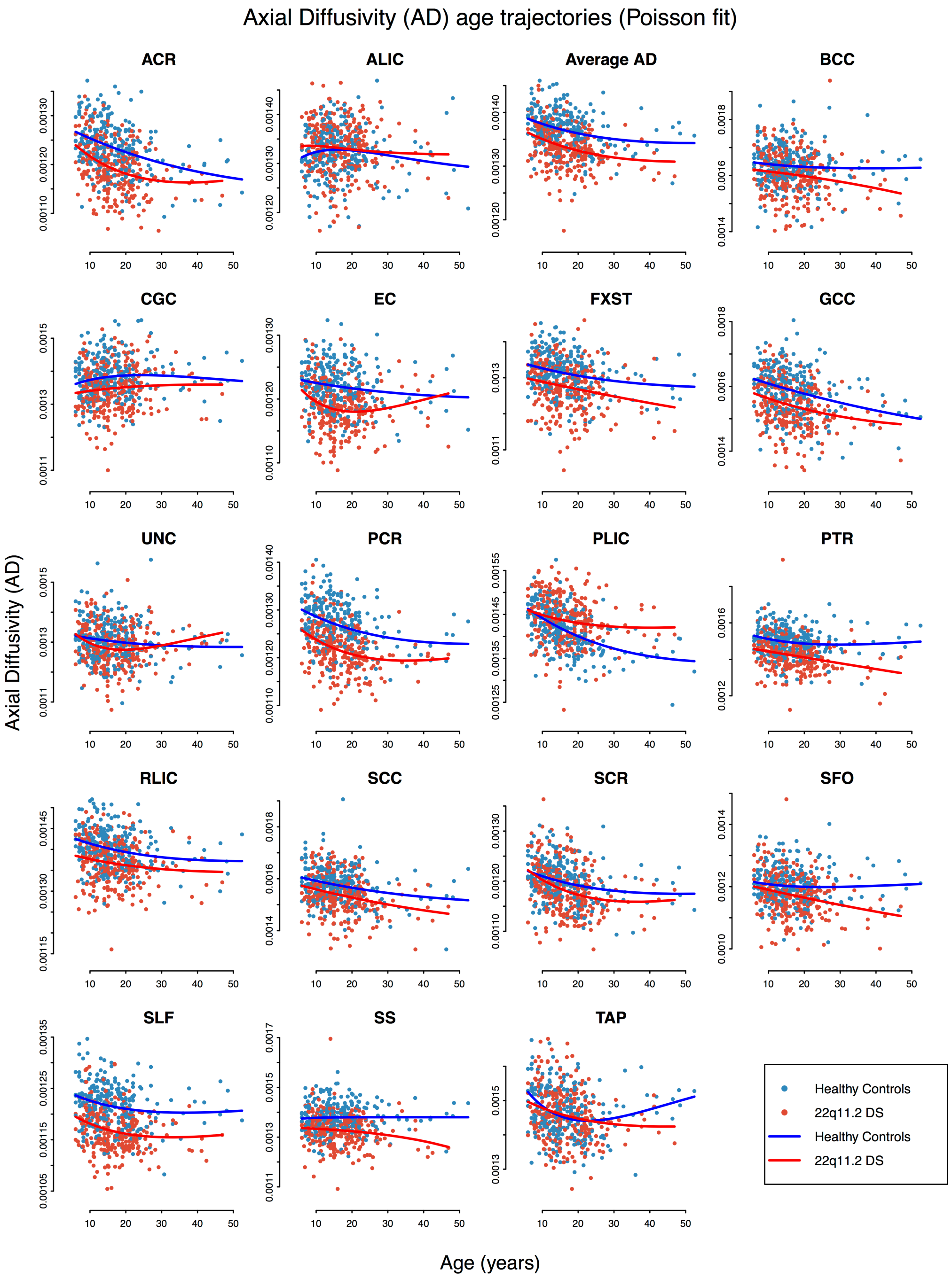

Supplementary Figure 6. Modulating effects of deletion type. Effect of deletion type on DTI measures for each ROI. Analyses compared 22q11DS subjects with A-D deletion type (N=206) against subjects with A-B deletion type (N=15).

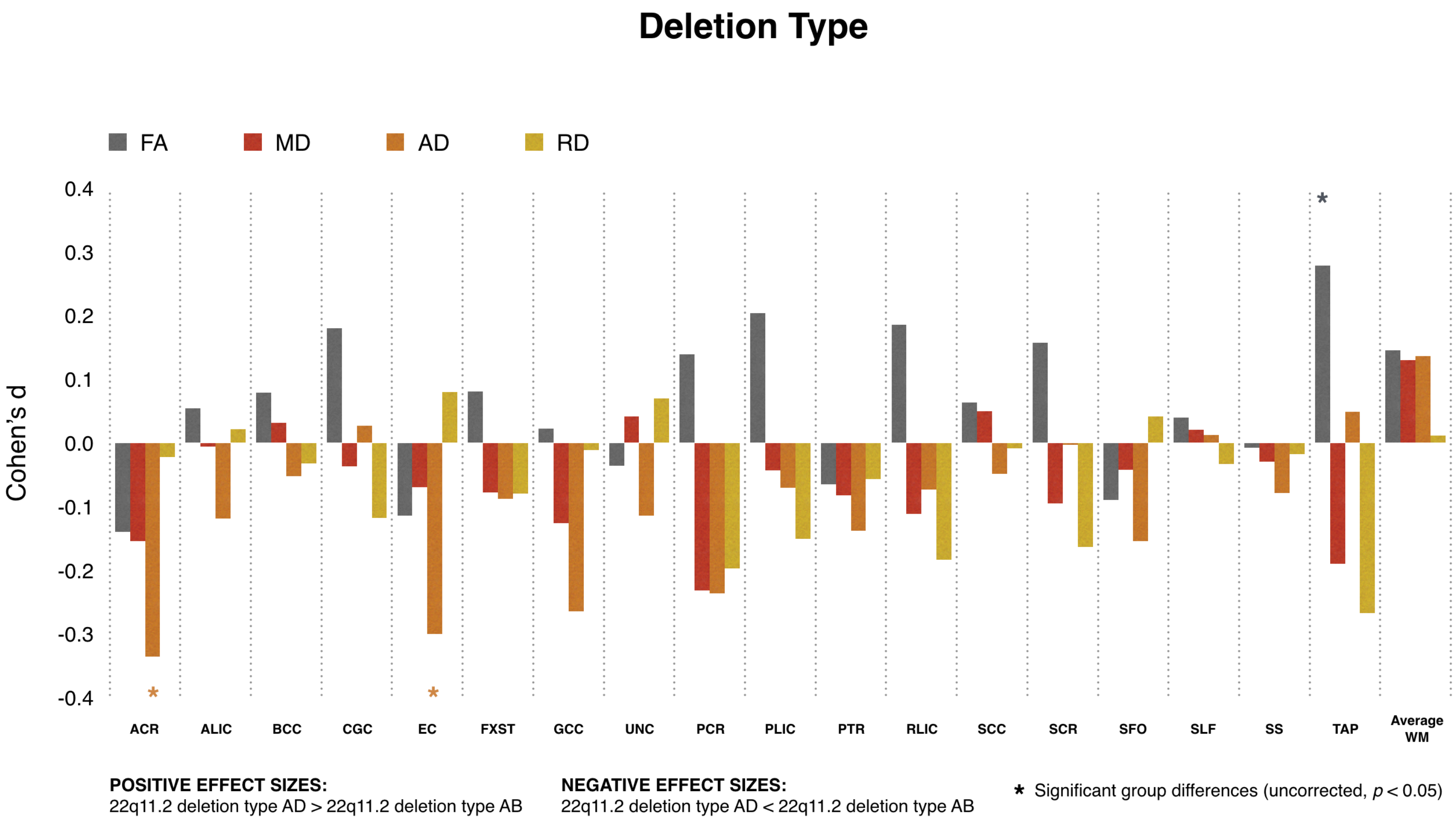

Supplementary Figure 7. Correlation between DTI measures and IQ for each ROI. Analyses were run separately for 22q11DS and Healthy Controls. Age, [Age-mean(Age)]^2 , and sex were included as covariates.

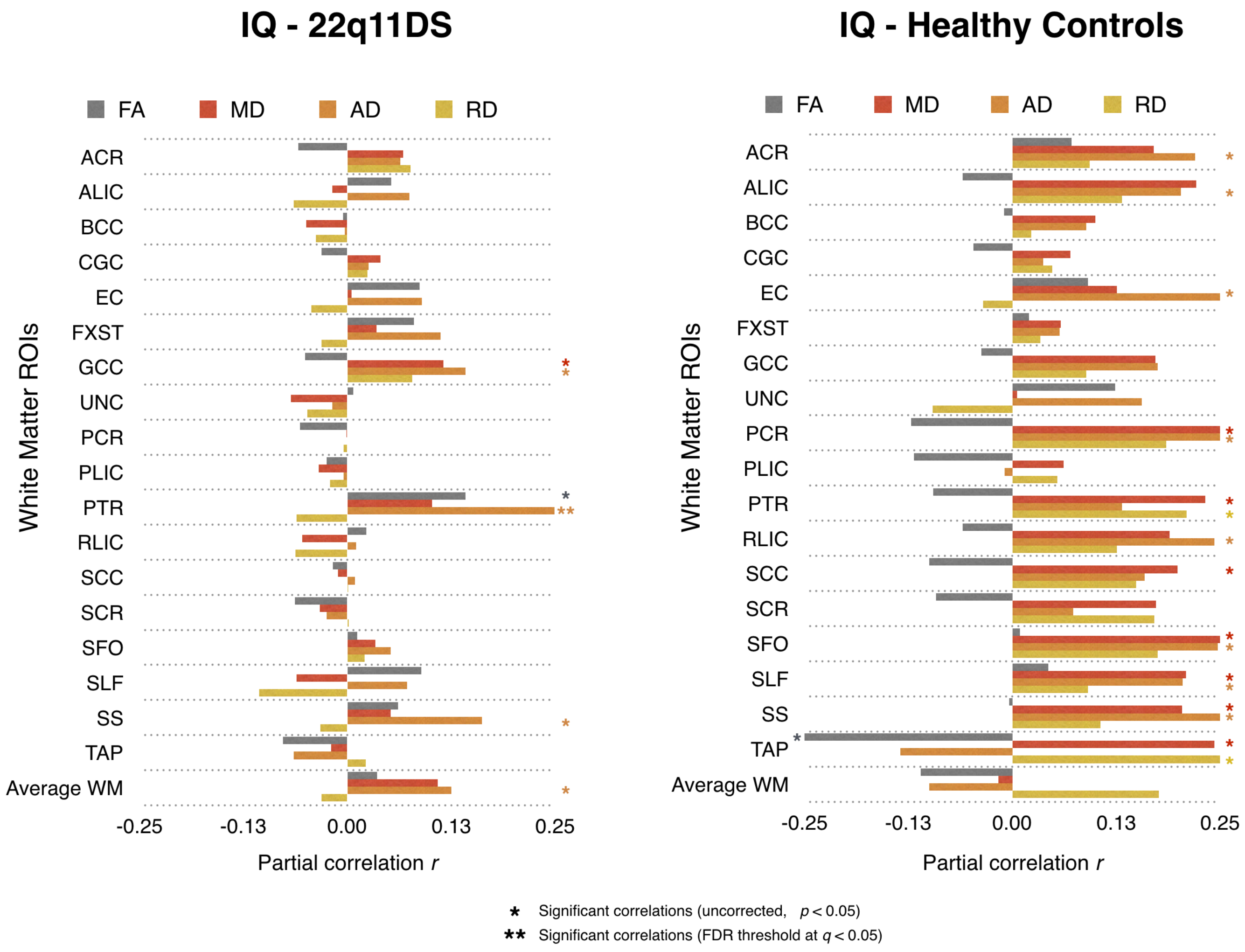

Supplement: Supplementary file 3 — Supplementary Figures [file 41380_2019_450_MOESM3_ESM.pdf]
